# Classification of dynamical Lie algebras of 2-local spin systems on linear, circular and fully connected topologies

**DOI:** 10.1038/s41534-024-00900-2

**Published:** 2024-11-06

**Authors:** Roeland Wiersema, Efekan Kökcü, Alexander F. Kemper, Bojko N. Bakalov

**Affiliations:** 1 Vector Institute, MaRS Centre, Toronto, ON M5G 1M1 Canada; 2 Department of Physics and Astronomy, University of Waterloo, Ontario, N2L 3G1 Canada; 3 Xanadu, Toronto, ON M5G 2C8 Canada; 4 Department of Physics, North Carolina State University, Raleigh, NC 27695 USA; 5 Department of Mathematics, North Carolina State University, Raleigh, NC 27695 USA

**Keywords:** Quantum mechanics, Qubits

## Abstract

Much is understood about 1-dimensional spin chains in terms of entanglement properties, physical phases, and integrability. However, the Lie algebraic properties of the Hamiltonians describing these systems remain largely unexplored. In this work, we provide a classification of all Lie algebras generated by the terms of 2-local spin chain Hamiltonians, or so-called dynamical Lie algebras, on 1-dimensional linear and circular lattice structures. We find 17 unique dynamical Lie algebras. Our classification includes some well-known models such as the transverse-field Ising model and the Heisenberg chain, and we also find more exotic classes of Hamiltonians that appear new. In addition to the closed and open spin chains, we consider systems with a fully connected topology, which may be relevant for quantum machine learning approaches. We discuss the practical implications of our work in the context of variational quantum computing, quantum control and the spin chain literature.

## Introduction

Mathematical classifications of the fundamental symmetries of physical systems date back to the work of Wigner, who proposed three symmetry classes of non-interacting fermionic Hamiltonians depending on their time-reversal and spin-rotation properties^1^. Three decades later, Dyson would mathematically solidify this theory and connect the spectral properties of these different types of Hamiltonians with random matrix theory^2^ (see ref.^3^ for a modern treatment). It would take another thirty years before Altland and Zinbauer extended these results to ten symmetry classes^4^, each of which correspond to a symmetric space in Cartan’s original classification of these spaces^5,6^. Further extensions of these results were made in recent years with regards to topological phases of matter^7–9^.

The above mathematical classifications in quantum physics rest on the powerful theory of Lie groups, which provides a framework for describing the continuous symmetries and transformations that characterize the behavior of quantum systems. The study of Lie groups, and by extension physical symmetries, can often be simplified by considering the corresponding Lie algebra of the group. The commutation relations of operators in the Lie algebra capture the essential features of the underlying symmetries and can be used to analyze the spectrum, eigenstates, and dynamics of quantum systems.

A Hamiltonian of a finite-dimensional system can be understood as (*i* times) an element of some Lie algebra ${\mathfrak{g}}\subseteq {\mathfrak{u}}(N)$. Here, ${\mathfrak{u}}(N)$ is the vector space of all skew-Hermitian *N* × *N* matrices equipped with the standard commutator. Typically, a Hamiltonian is described by a linear combination of terms that correspond to a certain physical interaction. The algebra formed by taking all (finite) products and linear combinations of the terms in a Hamiltonian is called the *bond algebra* of the Hamiltonian^10–12^. Bond algebras have been studied extensively to understand the symmetries and spectra of different classes of Hamiltonians. More recently, they have been used to study thermalization phenomena in quantum many-body systems^13,14^. Instead of studying the algebra formed by taking products of individual interactions in the Hamiltonian, we can study the Lie algebra generated by these terms under commutation. The result is called the Hamiltonian algebra or *dynamical Lie algebra* (DLA)^15–19^, which is intricately linked to the unitary dynamics of a quantum system.

Since each DLA is a subalgebra of ${\mathfrak{u}}(N)$, a classification of DLAs can be phrased as a classification of all subalgebras of ${\mathfrak{u}}(N)$. Such a classification is intractable, except when specific constraints are placed on the subalgebras one considers. For example, in the original works of Killing and Cartan, all *simple* Lie algebras were classified^20^; similarly, Dynkin provided a classification of the *maximal* subalgebras of simple Lie algebras^21,22^. We follow a different approach: instead of adding algebraic constraints such as simplicity or maximality, we make use of the fact that any Lie algebra can be described by a set of generators, and we consider the Lie algebras that arise by using the terms of specific Hamiltonians as the generators. In contrast with the previously-mentioned classifications of^1,2,4^, this approach covers interacting quantum many-body systems.

Specifically, we consider the class of Hamiltonians that correspond to 1-dimensional 2-local spin chains, and provide a classification of the Lie algebras that are generated under commutation by the individual Pauli terms of the Hamiltonian. Much about these systems is well-understood, from their entanglement properties^23^, their phases^24^ and their integrability^25,26^. Additionally, they show up in the study of bond algebras^10,14^. However, to the best of our knowledge, the Lie algebraic properties of these Hamiltonians have not yet been explored in full. It is thus reasonable to ask, given our comprehensive knowledge of the physics governing these systems, what more can be learned from the Lie algebra? We provide an answer to this question by classifying all unique dynamical Lie algebras of linear, circular and permutation invariant spin systems generated by 2-local Pauli operators. We discuss how our classification has bearing on areas of quantum control, variational quantum computing, and quantum dynamics and thermodynamics.

For *variational quantum computing*, one is not interested in representing the whole unitary group, but in using a parameterized subgroup in order to generate a state that minimizes a given objective function. Understanding what subgroup a particular quantum circuit parameterizes can give insight into its representational power. For example, one can connect the dimension of the DLA to a phenomenon called overparameterization^27–30^. Additionally, DLAs can be used to understand barren plateaus^31^ — flat areas in the cost landscape of a variational quantum algorithm that hinder optimization^32,33^. Finally, a recent work uses knowledge of the DLA to perform efficient classical simulations of several quantum algorithms^34^. We discuss these results by showing that barren plateau and overparmaeterization phenomena show for several examples found in our classification as expected, which highlights the usefulness of classifying sets of quantum circuits by their Lie algebraic properties.

In *quantum control*, the DLA of a dynamical quantum system can be related to the set of reachable states of that system. In particular, DLAs can be used to define a notion of controllability of a quantum system^35–37^, which is highly relevant when it comes to designing unitary operations for quantum simulators and quantum computers. One is typically interested in Hamiltonians that can generate an arbitrary unitary operator, while the existence of symmetries can inhibit the control of a physical system^38^.

Finally, one can use the knowledge of the DLA to provide insights into the *dynamics* of physical systems. Here, it should be noted that the physical properties of models described by the Hamiltonian whose terms generate the DLA strongly depend on the coefficients of said terms. Our classification, which neglects the coefficients, is thus limited to the properties that belong to the entire class of models described by the same terms. While this is a coarse classification, we can nevertheless make quite general observations just from the study of the DLA. For example, one can construct constant-depth quantum circuits for the dynamical simulation of a specific quantum system^39–42^, or state preparation via adiabatic state preparation^40,42^, or implement Hartree–Fock^43^. The dimension of the DLA is directly related to the quantum circuit depth needed to capture the full dynamics^39^. Additionally, non-Abelian commutants describe non-Abelian symmetries.

## Results

### Summary of the main results

Here, we give a brief summary of our main results, which include the classification of all DLAs generated by the Pauli terms of 2-local spin Hamiltonians of length *n* in one dimension. We emphasize that our method is not limited to one-dimensional topologies, but can be extend to other graphs, which we will explore in a follow-up work^44^.

Recall that a Lie algebra can be constructed by a set of generators so that it is closed under linear combinations and under the Lie bracket. In our case, the Lie bracket is the standard commutator [*A*, *B*] = *AB* − *BA*. We now choose the generators of our Lie algebra to be (*i* times) the Pauli strings that appear as terms of a 2-local spin chain Hamiltonian. Since a Hamiltonian is always a Hermitian operator, we can understand it as (*i* times) an element of the Lie algebra ${\mathfrak{u}}({2}^{n})$. Therefore, we can limit ourselves to the study of DLAs that are subalgebras of ${\mathfrak{u}}({2}^{n})$, for which we have the following useful fact. Although this result is known (see e.g.^38,45^), for completeness, we provide its proof and review the necessary definitions in SM A II.

#### Proposition 1.1

Any DLA must be either Abelian, isomorphic to ${\mathfrak{su}}({N}^{{\prime} })$, ${\mathfrak{so}}({N}^{{\prime} })$, ${\mathfrak{sp}}({N}^{\prime\prime})$ (with ${N}^{{\prime} }\le {2}^{n}$, *N*^*″*^≤2^*n*−1^), an exceptional compact simple Lie algebra, or a direct sum of such Lie algebras. Indeed, any subalgebra of ${\mathfrak{u}}(N)$ is either Abelian or a direct sum of compact simple Lie algebras and a center.

Note that all simple Lie algebras over the complex and real numbers have been classified by Killing and Cartan^46^. The above proposition forms the backbone of our classification, as we know that any DLA generated by our class of Hamiltonians must be of the described form.

To obtain our classification, we first calculate all DLAs that can be generated by Pauli strings of length 2. Then we identify the orbits under the symmetries of the Pauli group and the swap of the two sites, thus reducing the number of unique Lie algebras to 27. Next, we find several isomorphisms between some of the sets of generators, reducing the set of unique structures even further. Finally, we determine how these Lie algebras scale with system size as the number of spins grows beyond 2 sites. In this final step, we take the boundaries of the spin chain into account, since the Lie algebra will behave differently for open or periodic boundary conditions of the chain. The following is our main result.

#### Result 1.1

(Classification of spin chain DLAs). We provide a classification of all dynamical Lie algebras generated by the Pauli terms of 2-local spin Hamiltonians in one dimension. For both open and closed spin chains, there are 17 unique Lie algebras that can be generated by a spin chain Hamiltonian.

The formal statement of this result is presented in the main text with Theorems I.1 and I.2 along with a sketch of the proof.

The dimension of a DLA can be related to the trainability of variational quantum circuits, and may therefore be of high interest. Since we know the dimensions of all simple Lie algebras, a direct corollary of our result is the following.

#### Result 1.2

(Dimension scaling of DLAs). The dimension of any dynamical Lie algebra generated by the Pauli terms of a 2-local spin chain Hamiltonian of length *n* will scale as either *O*(4^*n*^), *O*(*n*^2^) or *O*(*n*).

**Fig. 1:**
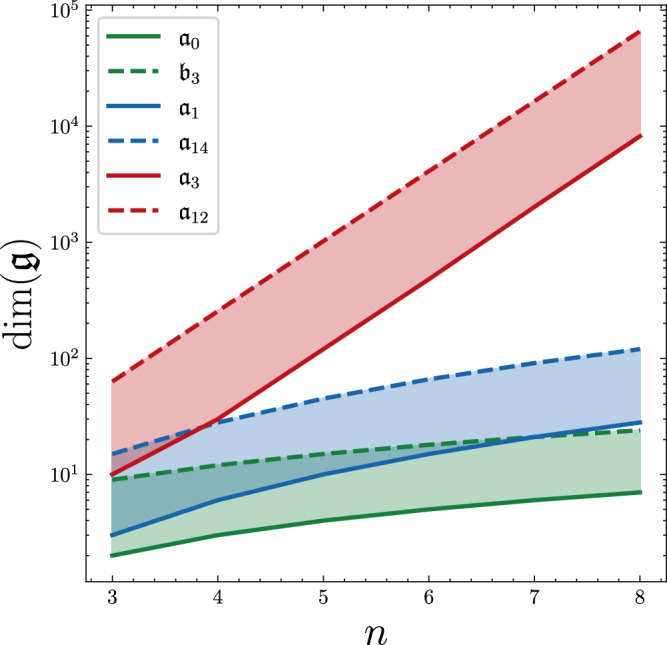
Scaling of the DLAs of spin chains with open boundary conditions. The exponentially scaling DLAs are denoted in red, the quadratically scaling ones in blue, and the linearly scaling algebras are denoted in green. The full and dashed line denote the smallest and largest scaling Lie algebra in our classification, respectively.

To illustrate this, we plot the dimensions of the open DLAs in our classification in Fig. 1.

Our proof technique also applies to the case of a complete graph, where each site is interacting with every other site via 2-local interactions; in other words, all-to-all connected. We therefore also include this topology in our classification.

#### Result 1.3

(Classification of permutation invariant DLAs). We provide a classification of all dynamical Lie algebras generated by the Pauli terms of 2-local permutation-invariant spin Hamiltonians. There are 8 unique Lie algebras that can be generated by such a spin chain Hamiltonian.

We present the formal statement of this result in Theorem I.3. Similarly to Result I.2, we find DLAs with linear, quadratic and exponentially scaling dimensions.

In addition to the classification of unique (up to isomorphism) Lie algebraic structures, we also provide an explicit list of examples of Hamiltonians that generate them in Table S.I. Some of the generators in our classification show up in well-known models such as the transverse-field Ising model or the Heisenberg model, whereas other Hamiltonians we find are perhaps not realizable in nature. However, some of these more exotic models may be of interest due to their properties. For instance, we find a class of Hamiltonians with globally non-commuting charges.

### Glossary

We summarize the main choices of notation and nomenclature used throughout the paper in the following list.

**Table:** 

${\mathfrak{a}}$, ${\mathfrak{g}}$	Lie algebras.
${\mathcal{A}}$, ${\mathcal{G}}$	Sets, usually generating sets for Lie algebras.
${\langle {\mathcal{G}}\rangle }_{{\rm{Lie}}}$	The Lie algebra generated by a set ${\mathcal{G}}$.
${\mathfrak{a}}\cong {\mathfrak{b}}$	${\mathfrak{a}}$ and ${\mathfrak{b}}$ are isomorphic as Lie algebras.
${\mathcal{A}}\equiv {\mathcal{B}}$	${\mathcal{A}}$ and ${\mathcal{B}}$ are equivalent under the symmetry group ${S}_{3}\times {{\mathbb{Z}}}_{2}$.
${\mathcal{A}}={\mathcal{B}}$	${\mathcal{A}}$ and ${\mathcal{B}}$ are equal as sets.
${{\mathcal{P}}}_{n}$	The set of *n*-qubit Pauli strings {*I*, *X*, *Y*, *Z*}^⊗*n*^.
*X*_*j*_, *Y*_*j*_, *Z*_*j*_	The action of the corresponding Pauli matrix on the *j*-th qubit in the spin chain.
${{\rm{span}}}_{{\mathbb{R}}}({\mathcal{A}})$	The set of all linear combinations of elements of ${\mathcal{A}}$ with real coefficients.
${\rm{span}}=i\,{{\rm{span}}}_{{\mathbb{R}}}$	A shortcut for taking the real span and multiplying by the imaginary unit *i*. Takes Hermitian matrices to skew-Hermitian.

### Lie algebras

We assume knowledge of finite-dimensional Lie algebras (for a formal treatment, see e.g. refs. ^6,46^), but will review some essential concepts here. A Lie algebra ${\mathfrak{g}}$ is a vector space equipped with a Lie bracket $[\cdot ,\cdot ]:{\mathfrak{g}}\times {\mathfrak{g}}\to {\mathfrak{g}}$ satisfying certain axioms (which are reviewed in SM A II). The Lie bracket defines the adjoint endomorphism ${{\rm{ad}}}_{a}:{\mathfrak{g}}\to {\mathfrak{g}}$ where ad_*a*_(*b*) = [*a*, *b*]. For our purposes, the Lie bracket is the standard commutator of linear operators on a vector space: [*a*, *b*] = *ab* − *ba*. When ${\mathfrak{g}}$ is a compact simple Lie algebra (cf. SM A II and Proposition I.1), one can associate to it a compact Lie group *G* via the exponential map $G={e}^{{\mathfrak{g}}}$.

Consider a set of generators ${\mathcal{A}}=\{{a}_{1},{a}_{2},\ldots ,{a}_{M}\}$ with ${a}_{k}\in {\mathfrak{g}}$. We first define the nested commutator,

1 $${{\rm{ad}}}_{{a}_{{i}_{1}}}\cdots {{\rm{ad}}}_{{a}_{{i}_{r}}}({a}_{j})=[{a}_{{i}_{1}},[{a}_{{i}_{2}},[\cdots [{a}_{{i}_{r}},{a}_{j}]\cdots \,]]],$$

which is just *a*_*j*_ in the special case *r* = 0. The linear span of all nested commutators

$${\langle {\mathcal{A}}\rangle }_{{\rm{Lie}}}:= {{\rm{span}}}_{{\mathbb{R}}}\{{{\rm{ad}}}_{{a}_{{i}_{1}}}\cdots {{\rm{ad}}}_{{a}_{{i}_{r}}}({a}_{j})\,\left\vert \right.\,{a}_{{i}_{1}},\ldots ,{a}_{{i}_{r}},{a}_{j}\in {\mathcal{A}}\}$$

is then called a *dynamical Lie algebra* (DLA)^15,45^. This is the minimal (under inclusion) subalgebra of ${\mathfrak{g}}$ that contains the set ${\mathcal{A}}$. The depth *r* of the nested commutator is finite and will depend on the size of the DLA, which we typically do not know beforehand. In practice, the DLA of a given set of generators ${\mathcal{A}}$ can be obtained recursively with Algorithm 1.

#### Algorithm 1

Calculating the DLA


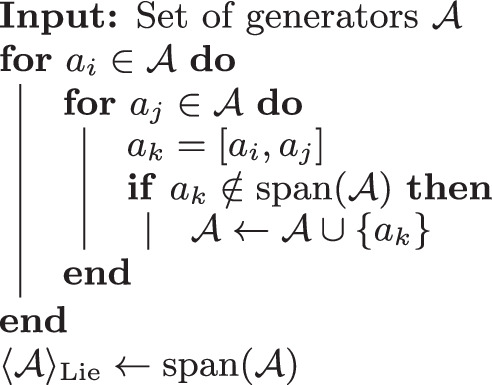


#### Remark 1.1

In this paper, we are interested in the case where the generators ${\mathcal{A}}$ are Pauli strings. Then, since the commutator of two Pauli strings is up to a scalar again a Pauli string, Algorithm 1 can be simplified by replacing the condition ${a}_{k}\,\notin\, {\rm{span}}({\mathcal{A}})$ with ${a}_{k}\,\notin\, {\mathcal{A}}\cup \{0\}$. Moreover, as different Pauli strings are linearly independent, the final set ${\mathcal{A}}$ will be a basis for the DLA ${\langle {\mathcal{A}}\rangle }_{{\rm{Lie}}}$.

### 2-local spin chains

Due to Proposition I.1, we know what form the subalgebras of ${\mathfrak{u}}(N)$ must take. Our goal is to find which of these direct sums of simple or Abelian Lie algebras can be generated by a physically inspired set of generators.

In particular, we are interested in the subalgebras of ${\mathfrak{u}}({2}^{n})$ that are generated by the terms of 1-dimensional 2-local Hamiltonians. By this we mean that we consider operators that couple up to two neighboring sites and where the operator interactions between neighboring sites is the same, but the interaction strength may vary per interaction, and could potentially depend on time due to an external field allowing control over the system.

In more details, we consider a spin system with a complex Hilbert space ${({{\mathbb{C}}}^{2})}^{\otimes n}$. On each copy of ${{\mathbb{C}}}^{2}$, representing a *qubit*, we have an action of the set of Pauli matrices ${{\mathcal{P}}}_{1}:= \{I,X,Y,Z\}$, while the 2-qubit interactions will be determined by some subset ${\mathcal{A}}\subseteq {{\mathcal{P}}}_{2}:= {{\mathcal{P}}}_{1}^{\otimes 2}$. We assume that ${\mathcal{A}}$ does not contain *I* ⊗ *I*, which corresponds to no interaction. A corresponding Hamiltonian that is built out of these interactions has the form

2 $$H=\mathop{\sum }\limits_{k=1}^{n-1}\sum _{a\otimes {a}^{{\prime} }\in {\mathcal{A}}}{J}_{k,a,{a}^{{\prime} }}(t){a}_{k}\otimes {a}_{k+1}^{{\prime} },$$

where ${J}_{k,a,{a}^{{\prime} }}$ are arbitrary real coefficients which are possibly time-dependent, and

3 $${a}_{k}\otimes {a}_{k+1}^{{\prime} }:= {I}^{\otimes (k-1)}\otimes a\otimes {a}^{{\prime} }\otimes {I}^{\otimes (n-k-1)}.$$

In the parlance of quantum computing and physics, *H* is a 2-local Hamiltonian corresponding to a *spin chain* with open boundary conditions. Closed or periodic boundary conditions are also of interest; in this case the Hamiltonian has the form

4 $$H=\mathop{\sum }\limits_{k=1}^{n}\sum _{a\otimes {a}^{{\prime} }\in {\mathcal{A}}}{J}_{k,a,{a}^{{\prime} }}(t){a}_{k}\otimes {a}_{k+1}^{{\prime} },$$

where the sites are viewed mod *n* so that ${a}_{n+1}^{{\prime} }:= {a}_{1}^{{\prime} }$ and

5 $${a}_{n}\otimes {a}_{n+1}^{{\prime} }:= {a}^{{\prime} }\otimes {I}^{\otimes (n-2)}\otimes a.$$

Note that physical models come with coefficients ${J}_{k,a,{a}^{{\prime} }}(t)$ in front of each ${a}_{k}\otimes {a}_{k+1}^{{\prime} }$ term that determine the equilibrium and non-equilibrium physics of the model. Here, we are only concerned with the Lie algebraic properties of the Hamiltonian *H* and we will not consider any spectral properties of *H*. Additionally, we note that we are considering a *directed* interaction graph, since we allow terms like *X* ⊗ *Y*, which do not act symmetrically between neighboring qubits.

Continuing, we note that the Pauli matrices $i{{\mathcal{P}}}_{1}\setminus \{iI\}$ form a basis of ${\mathfrak{su}}(2)$, and the tensor products $i{{\mathcal{P}}}_{2}\setminus \{i{I}^{\otimes 2}\}$ form a basis of ${\mathfrak{su}}(4)$. Hence, ${\rm{span}}({\mathcal{A}})\subseteq {\mathfrak{su}}(4)$ (recall that ${\rm{span}}=i\,{{\rm{span}}}_{{\mathbb{R}}}$). In the following, we will suppress the tensor product between Pauli operators and identities for clarity, and we denote $a\otimes {a}^{{\prime} }=a{a}^{{\prime} }$. We now give some examples to illustrate how several well-known spin chains can be written in this notation.

#### Example I.1

*Random field Ising model*. The Hamiltonian of the transverse-field Ising model (TFIM) in one dimension with open boundary conditions and random transverse field is given by

$${H}_{{\rm{TFIM}}}=J\mathop{\sum }\limits_{i=1}^{n-1}{Z}_{i}{Z}_{i+1}+\mathop{\sum }\limits_{i=1}^{n}{g}_{i}{X}_{i},$$

where $J,{g}_{i}\in {\mathbb{R}}$. Its generating set of Pauli strings is

$${{\mathcal{A}}}_{{\rm{TFIM}}}:= \{ZZ,XI,IX\}.$$

#### Example I.2

*XXZ chain*. For the 1-dimensional XXZ chain with open boundary conditions, the Hamiltonian is given by

$${H}_{{\rm{XXZ}}}=\mathop{\sum }\limits_{i=1}^{n-1}({X}_{i}{X}_{i+1}+{Y}_{i}{Y}_{i+1}+{{\Delta }}{Z}_{i}{Z}_{i+1}),$$

which has generators

$${{\mathcal{A}}}_{{\rm{XXZ}}}:= \{XX,YY,ZZ\}.$$

#### Example I.3

*Spinless fermionic Gaussian state*. A free fermion Hamiltonian chain in one dimension with periodic boundary conditions can be built from the generators on two sites:

$${c}_{1}^{\dagger }{c}_{2}^{\dagger },{c}_{1}^{\dagger }{c}_{1},{c}_{2}^{\dagger }{c}_{2},{c}_{1}^{\dagger }{c}_{2},{c}_{2}^{\dagger }{c}_{1},{c}_{1}{c}_{2},$$

where *c*^†^ and *c* are fermionic raising and lowering operators, respectively. The corresponding Hamiltonian is

$${H}_{{\rm{FF}}}=\mathop{\sum }\limits_{i=1}^{n}({c}_{i}^{\dagger }{c}_{i+1}^{\dagger }+{c}_{i}^{\dagger }{c}_{i+1}+{c}_{i}{c}_{i+1}^{\dagger }+{c}_{i}{c}_{i+1}),$$

where *c*_*n*+1_ ≔ *c*_1_ and ${c}_{n+1}^{\dagger }:= {c}_{1}^{\dagger }$. The fermionic raising and lowering operators may be translated to Pauli string form via a number of transformations. If we use the common Jordan–Wigner transformation, the resulting set of Pauli generators is

$${{\mathcal{A}}}_{{\rm{FF}}}:= \left\{XX,ZI,IZ,YY,XY,YX\right\}.$$

We can now state the central question of our work. We seek to classify all DLAs generated by the terms (3) (respectively, (3) and (5)) of a Hamiltonian (2) (respectively, (4)) of a spin chain with both open and periodic boundary conditions. We first determine (up to symmetry) all subalgebras of ${\mathfrak{su}}(4)$ that are generated by all possible interaction sets ${\mathcal{A}}\subseteq {{\mathcal{P}}}_{2}$, and then we discuss how such subalgebras extend to *n* sites to give subalgebras of ${\mathfrak{su}}({2}^{n})$.

### Growing the dynamical Lie algebras

On a 2-qubit system, the Pauli interaction terms ${\mathcal{A}}\subseteq {{\mathcal{P}}}_{2}$ generate a Lie algebra ${\langle {\mathcal{A}}\rangle }_{{\rm{Lie}}}={\mathfrak{a}}$, which is a subalgebra of ${\mathfrak{su}}(4)$. We now investigate the structure of the DLA as we add terms that have been translated by one site. In the case of an *open* spin chain, we define the DLA ${\mathfrak{a}}(n)$ as the subalgebra of ${\mathfrak{su}}({2}^{n})$ generated by the terms ${a}_{k}\otimes {a}_{k+1}^{{\prime} }$ for $a\otimes {a}^{{\prime} }\in {\mathcal{A}}$, *k* = 1, …, *n* − 1 (see (3)). Equivalently, we note that ${\mathfrak{a}}(n)$ is generated by the set

$$\bigcup _{1\le k\le n-1}{I}^{\otimes (k-1)}\otimes {\mathfrak{a}}\otimes {I}^{\otimes (n-k-1)}.$$

**Fig. 2:**
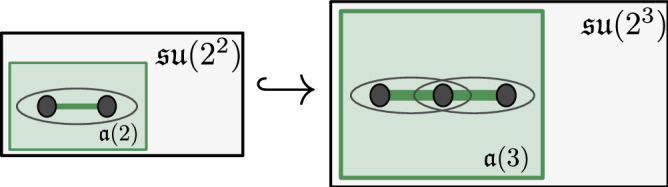
Growing a Lie algebra by adding a site to the chain. Increasing the system size from 2 sites to 3 sites changes the DLA from ${\mathfrak{a}}(2)$ to ${\mathfrak{a}}(3)$ within ${\mathfrak{su}}(4)$ and ${\mathfrak{su}}(8)$, respectively. The goal of our classification is to describe the behavior for any *n* ≥ 3.

In particular, ${\mathfrak{a}}(2)={\mathfrak{a}}$. By construction, we have two Lie algebra embeddings ${\mathfrak{a}}(n)\hookrightarrow {\mathfrak{a}}(n+1)$, given by appending *I* to the first or last qubit (see Fig. 2).

For a *closed* spin chain with periodic boundary conditions, the DLA is generated by the terms ${a}_{k}\otimes {a}_{k+1}^{{\prime} }$ for $a\otimes {a}^{{\prime} }\in {\mathcal{A}}$, *k* = 1, …, *n*, where ${a}_{n+1}^{{\prime} }:= {a}_{1}^{{\prime} }$ (see (3), (5)). We denote the resulting Lie algebra as ${{\mathfrak{a}}}^{\circ }(n)$.

#### Example I.4

Consider the generating set ${\mathcal{A}}=\{XY\}$ on 2 qubits. The DLA is given by

$${\mathfrak{a}}={\langle {\mathcal{A}}\rangle }_{{\rm{Lie}}}={\rm{span}}\{XY\},$$

which is an Abelian Lie algebra isomorphic to ${\mathfrak{u}}(1)$. Constructing the generators of ${\mathfrak{a}}(3)$ according to the procedure above gives

$${\mathfrak{a}}(3)={\langle XYI,IXY\rangle }_{{\rm{Lie}}}={\rm{span}}\{XYI,IXY,XZY\}.$$

It is easy to confirm that ${\mathfrak{a}}(3)\cong {\mathfrak{so}}(3)$. We see that going from *n* = 2 to *n* = 3, the DLA changes from ${\mathfrak{u}}(1)$ to ${\mathfrak{so}}(3)$.

#### Example I.5

For the generating set ${\mathcal{A}}=\{XY\}$ with ${\mathfrak{a}}={\rm{span}}\{XY\}$, the DLA of the 3-site closed spin chain is

$$\begin{array}{ll}{{\mathfrak{a}}}^{\circ }(3)\,={\langle XYI,IXY,YIX\rangle }_{{\rm{Lie}}}\\ \qquad\quad={\rm{span}}\{XYI,IXY,XZY,YIX,YXZ,ZYX\}.\end{array}$$

One can show that ${{\mathfrak{a}}}^{\circ }(3)\cong {\mathfrak{so}}(3)\oplus {\mathfrak{so}}(3)$.

The above examples illustrate that the algebraic structure of a DLA can change as we increase the system size, and moreover it depends on the boundary conditions.

### Main theorem

We state the main theorem of our work below, and tabulate the generators of the Lie algebras of our classification in Table 1.

**Table 1:** List of generators of the DLAs in Theorem I.1 and examples of conventional spin models that have the equivalent DLA

Label	Generating set	Example Model
${{\mathfrak{a}}}_{0}$	*XX*	Ising model
${{\mathfrak{a}}}_{1}$	*XY*	Kitaev chain
${{\mathfrak{a}}}_{2}$	*XY*, *YX*	Massless free fermion + magnetic field
${{\mathfrak{a}}}_{3}$	*XX*, *YZ*	Kitaev chain + Coulomb
${{\mathfrak{a}}}_{4}$	*XX*, *YY*	XY-model
${{\mathfrak{a}}}_{5}$	*XY*, *YZ*	
${{\mathfrak{a}}}_{6}$	*XX*, *YZ*, *ZY*	Massless free fermion + magnetic field + Coulomb
${{\mathfrak{a}}}_{7}$	*XX*, *YY*, *ZZ*	Heisenberg chain
${{\mathfrak{a}}}_{8}$	*XX*, *XZ*	Ising model + transverse field
${{\mathfrak{a}}}_{9}$	*XY*, *XZ*	Kitaev chain + longitudinal field
${{\mathfrak{a}}}_{10}$	*XY*, *YZ*, *ZX*	Heisenberg
${{\mathfrak{a}}}_{11}$	*XY*, *YX*, *YZ*	XY-model + longitudinal field
${{\mathfrak{a}}}_{12}$	*XX*, *XY*, *YZ*	
${{\mathfrak{a}}}_{13}$	*XX*, *YY*, *YZ*	XY-model + longitudinal field
${{\mathfrak{a}}}_{14}$	*XX*, *YY*, *XY*	XY-model + transverse field
${{\mathfrak{a}}}_{15}$	*XX*, *XY*, *XZ*	Ising model + arbitrary field
${{\mathfrak{a}}}_{16}$	*XY*, *YX*, *YZ*, *ZY*	Kitaev chain + longitudinal field
${{\mathfrak{a}}}_{17}$	*XX*, *XY*, *ZX*	Ising model + arbitrary field
${{\mathfrak{a}}}_{18}$	*XX*, *XZ*, *YY*, *ZY*	XY-model + arbitrary field
${{\mathfrak{a}}}_{19}$	*XX*, *XY*, *ZX*, *YZ*	
${{\mathfrak{a}}}_{20}$	*XX*, *YY*, *ZZ*, *ZY*	Heisenberg chain + magnetic field
${{\mathfrak{a}}}_{21}$	*XX*, *YY*, *XY*, *ZX*	XY-model + arbitrary field
${{\mathfrak{a}}}_{22}$	*XX*, *XY*, *XZ*, *YX*	Ising model + arbitrary field
${{\mathfrak{b}}}_{0}$	*XI*, *IX*	Uncoupled spins
${{\mathfrak{b}}}_{1}$	*XX*, *XI*, *IX*	Ising model
${{\mathfrak{b}}}_{2}$	*XY*, *XI*, *IX*	Kitaev chain + longitudinal field
${{\mathfrak{b}}}_{3}$	*XI*, *YI*, *IX*, *IY*	Uncoupled spins
${{\mathfrak{b}}}_{4}$	*XX*, *XY*, *XI*, *IX*	Ising model + arbitrary field

Considering our construction of DLAs, the example Hamiltonians can be generalized by making all coefficients of Pauli strings independent from each other.

We separate the dynamical Lie algebras in our classification into two types. A *DLA of* ${\mathfrak{a}}$-*type* on an open spin chain of length *n* is a Lie algebra of the form ${\mathfrak{a}}(n)$, where ${\mathfrak{a}}={\langle {\mathcal{A}}\rangle }_{{\rm{Lie}}}$ for some generating set of 2-site Pauli strings ${\mathcal{A}}\subseteq {\{X,Y,Z\}}^{\otimes 2}$. Explicitly, ${\mathfrak{a}}(n)={\langle {\mathcal{A}}(n)\rangle }_{{\rm{Lie}}}$ where

6 $${\mathcal{A}}(n)=\{{A}_{i}{B}_{i+1}\,| \,AB\in {\mathcal{A}},\,1\le i\le n-1\},$$

with *AB* denoting 2-site Pauli strings, *A*, *B* ∈ {*X*, *Y*, *Z*}.

A ${\mathfrak{b}}$-*type DLA* on an open spin chain of length *n* is a DLA that cannot be expressed as ${\mathfrak{a}}$-type and has the form ${\mathfrak{b}}(n)={\langle {\mathcal{A}}(n)\cup {\mathcal{B}}(n)\rangle }_{{\rm{Lie}}}$ for some ${\mathcal{A}}\subseteq {\{X,Y,Z\}}^{\otimes 2}$, a non-empty set of Paulis ${\mathcal{B}}\subseteq \{X,Y,Z\}$, and

7 $${\mathcal{B}}(n)=\{{B}_{i}\,| \,B\in {\mathcal{B}},\,1\le i\le n\}.$$

We have generated all DLAs ${{\mathfrak{a}}}_{k}(n)$, ${{\mathfrak{b}}}_{k}(n)$, ${{\mathfrak{a}}}_{k}^{\circ }(n)$ and ${{\mathfrak{b}}}_{k}^{\circ }(n)$ up to *n* = 8 with Algorithm 1 and Remark I.1. For *n* = 3, we present linear bases of all these Lie algebras in SM B V. Inspired by this list of Lie algebras, we construct formal proofs to determine them for all *n* ≥ 3.

#### Theorem 1.1

(Classification of Open DLAs). Every dynamical Lie algebra of type ${\mathfrak{a}}$ or ${\mathfrak{b}}$ on an open spin chain of length *n* ≥ 3 is isomorphic to one of the following:

$$\begin{array}{ll}{{\mathfrak{a}}}_{0}(n)\cong {\mathfrak{u}}{(1)}^{\oplus (n-1)},\\ {{\mathfrak{a}}}_{1}(n)\cong {\mathfrak{so}}(n),\\ {{\mathfrak{a}}}_{2}(n)\cong {{\mathfrak{a}}}_{4}(n)\cong {\mathfrak{so}}(n)\oplus {\mathfrak{so}}(n),\\ {{\mathfrak{a}}}_{3}(n)\cong \left\{\begin{array}{ll}{\mathfrak{so}}{({2}^{n-2})}^{\oplus 4},\quad n\equiv 0\,{\rm{mod}}\,8,\\ {\mathfrak{so}}({2}^{n-1}),\qquad n\equiv \pm 1\,{\rm{mod}}\,8,\\ {\mathfrak{su}}{({2}^{n-2})}^{\oplus 2},\quad n\equiv \pm 2\,{\rm{mod}}\,8,\\ {\mathfrak{sp}}({2}^{n-2}),\qquad n\equiv \pm 3\,{\rm{mod}}\,8,\\ {\mathfrak{sp}}{({2}^{n-3})}^{\oplus 4},\quad n\equiv 4\,{\rm{mod}}\,8,\end{array}\right.\\ {{\mathfrak{a}}}_{5}(n)\cong \left\{\begin{array}{ll}{\mathfrak{so}}{({2}^{n-2})}^{\oplus 4},\quad n\equiv 0\,{\rm{mod}}\,6,\\ {\mathfrak{so}}({2}^{n-1}),\qquad n\equiv \pm 1\,{\rm{mod}}\,6,\\ {\mathfrak{su}}{({2}^{n-2})}^{\oplus 2},\quad n\equiv \pm 2\,{\rm{mod}}\,6,\\ {\mathfrak{sp}}({2}^{n-2}),\qquad n\equiv 3\,{\rm{mod}}\,6,\end{array}\right.\\ {{\mathfrak{a}}}_{6}(n)\cong {{\mathfrak{a}}}_{7}(n)\cong {{\mathfrak{a}}}_{10}(n)\\ \qquad\quad\cong \left\{\begin{array}{ll}{\mathfrak{su}}({2}^{n-1}),\qquad n\,\,{\rm{odd}},\\ {\mathfrak{su}}{({2}^{n-2})}^{\oplus 4},\quad n\ge 4\,\,{\rm{even}},\end{array}\right.\\ {{\mathfrak{a}}}_{8}(n)\cong {\mathfrak{so}}(2n-1),\\ {{\mathfrak{a}}}_{9}(n)\cong {\mathfrak{sp}}({2}^{n-2}),\\ {{\mathfrak{a}}}_{11}(n)={{\mathfrak{a}}}_{16}(n)={\mathfrak{so}}({2}^{n}),\quad n\ge 4,\\ {{\mathfrak{a}}}_{k}(n)={\mathfrak{su}}({2}^{n}),\,\,k=12,17,18,19,21,22,\,n\ge 4,\\ {{\mathfrak{a}}}_{13}(n)={{\mathfrak{a}}}_{20}(n)\cong {{\mathfrak{a}}}_{15}(n)\cong {\mathfrak{su}}{({2}^{n-1})}^{\oplus 2},\\ {{\mathfrak{a}}}_{14}(n)\cong {\mathfrak{so}}(2n),\\ {{\mathfrak{b}}}_{0}(n)\cong {\mathfrak{u}}{(1)}^{\oplus n},\\ {{\mathfrak{b}}}_{1}(n)\cong {\mathfrak{u}}{(1)}^{\oplus (2n-1)},\\ {{\mathfrak{b}}}_{2}(n)\cong {\mathfrak{sp}}({2}^{n-2})\oplus {\mathfrak{u}}(1),\\ {{\mathfrak{b}}}_{3}(n)\cong {\mathfrak{su}}{(2)}^{\oplus n},\\ {{\mathfrak{b}}}_{4}(n)\cong {\mathfrak{su}}({2}^{n-1})\oplus {\mathfrak{su}}({2}^{n-1})\oplus {\mathfrak{u}}(1).\end{array}$$

We present a sketch of the proof of Theorem I.1 in the Methods section, and the full proof can be found in the Supplemental Material. The following corollary is an immediate consequence of Theorem I.1 and knowledge of the dimensions of the Lie algebras ${\mathfrak{su}}$, ${\mathfrak{so}}$ and ${\mathfrak{sp}}$ (see SM (A9)). As can be seen from Theorem I.2 below, it holds for both open and closed spin chains.

#### Corollary 1.1

(Dimension scaling of DLAs). The dimensions of all dynamical Lie algebras of 2-local spin chains of length *n* scale as either *O*(4^*n*^), *O*(*n*^2^) or *O*(*n*).

We thus see that the DLAs can be separated in three classes based on the scaling of their dimensions.

### Periodic boundary conditions

In the periodic case, we define a *DLA of* ${{\mathfrak{a}}}^{\circ }$-*type* on a closed spin chain of length *n* as a Lie algebra of the form ${{\mathfrak{a}}}^{\circ }(n)={\langle {{\mathcal{A}}}^{\circ }(n)\rangle }_{{\rm{Lie}}}$ for some generating set of 2-site Pauli strings ${\mathcal{A}}\subseteq {\{X,Y,Z\}}^{\otimes 2}$, where

8 $${{\mathcal{A}}}^{\circ }(n)=\{{A}_{i}{B}_{i+1},{A}_{n}{B}_{1}\,| \,AB\in {\mathcal{A}},\,1\le i\le n-1\}.$$

A ${{\mathfrak{b}}}^{\circ }$-*type DLA* on a closed spin chain of length *n* is one that cannot be expressed as ${{\mathfrak{a}}}^{\circ }$-type and has the form ${{\mathfrak{b}}}^{\circ }(n)={\langle {{\mathcal{A}}}^{\circ }(n)\cup {\mathcal{B}}(n)\rangle }_{{\rm{Lie}}}$ for some ${\mathcal{A}}\subseteq {\{X,Y,Z\}}^{\otimes 2}$, a non-empty set of Paulis ${\mathcal{B}}\subseteq \{X,Y,Z\}$, and ${\mathcal{B}}(n)$ given by (7).

#### Theorem 1.2

(Classification of Periodic DLAs). Every dynamical Lie algebra of type ${{\mathfrak{a}}}^{\circ }$ or ${{\mathfrak{b}}}^{\circ }$ on a closed spin chain of length *n* ≥ 3 is isomorphic to one of the following:

$$\begin{array}{ll}{{\mathfrak{a}}}_{0}^{\circ }(n)\cong {\mathfrak{u}}{(1)}^{\oplus n},\\ {{\mathfrak{a}}}_{1}^{\circ }(n)\cong {\mathfrak{so}}{(n)}^{\oplus 2},\\ {{\mathfrak{a}}}_{2}^{\circ }(n)\cong {\mathfrak{so}}{(n)}^{\oplus 4},\\ {{\mathfrak{a}}}_{3}^{\circ }(n)=\left\{\begin{array}{ll}{{\mathfrak{a}}}_{13}(n),\quad\,\,\,\, n\,\,{\rm{odd}},\\ {{\mathfrak{a}}}_{3}(n),\qquad n\equiv 0\,{\mathrm{mod}}\,\,4,\\ {{\mathfrak{a}}}_{6}(n),\qquad n\equiv 2\,{\mathrm{mod}}\,\,4,\end{array}\right.\\ \qquad\quad\cong \left\{\begin{array}{ll}{\mathfrak{su}}{({2}^{n-1})}^{\oplus 2},\quad\,\,\,\, n\,\,{\rm{odd}},\\ {\mathfrak{so}}{({2}^{n-2})}^{\oplus 4},\qquad n\equiv 0\,{\mathrm{mod}}\,\,8,\\ {\mathfrak{sp}}{({2}^{n-3})}^{\oplus 4},\qquad n\equiv 4\,{\mathrm{mod}}\,\,8,\\ {\mathfrak{su}}{({2}^{n-2})}^{\oplus 4},\qquad n\equiv 2\,{\mathrm{mod}}\,\,4,\end{array}\right.\\ {{\mathfrak{a}}}_{4}^{\circ }(n)\cong \left\{\begin{array}{l}{\mathfrak{so}}(2n),\quad \,n\,\,{\rm{odd}},\qquad \\ {\mathfrak{so}}{(n)}^{\oplus 4},\quad\, n\,{\rm{even}},\qquad \end{array}\right.\\ {{\mathfrak{a}}}_{5}^{\circ}(n)=\left\{\begin{array}{ll}{{\mathfrak{a}}}_{16}(n),\quad\,\,\, n\equiv \pm 1\,{\mathrm{mod}}\,\,3,\\ {{\mathfrak{a}}}_{5}(n),\qquad n\equiv 0\,{\mathrm{mod}}\,\,3,\end{array}\right.\\ \qquad\quad\cong \left\{\begin{array}{ll}{\mathfrak{so}}({2}^{n}),\qquad n\equiv \pm 1\,{\mathrm{mod}}\,\,3,\\ {\mathfrak{so}}{({2}^{n-2})}^{\oplus 4}, n\equiv 0\,{\mathrm{mod}}\,\,6,\\ {\mathfrak{sp}}({2}^{n-2}),\quad{n}\equiv 3\,{\mathrm{mod}}\,\,6,\end{array}\right.\\ {{\mathfrak{a}}}_{6}^{\circ }(n)=\left\{\begin{array}{ll}{{\mathfrak{a}}}_{13}(n)\cong {\mathfrak{su}}{({2}^{n-1})}^{\oplus 2},\qquad {n}\,\,{\rm{odd}},\\ {{\mathfrak{a}}}_{6}(n)\cong {\mathfrak{su}}{({2}^{n-2})}^{\oplus 4},\qquad n\,\,{\rm{even}},\end{array}\right.\\ {{\mathfrak{a}}}_{k}^{\circ }(n)={{\mathfrak{a}}}_{k}(n),\qquad k=7,13,16,20,\\ {{\mathfrak{a}}}_{8}^{\circ }(n)\cong {\mathfrak{so}}{(2n)}^{\oplus 2},\\ {{\mathfrak{a}}}_{9}^{\circ }(n)\cong {\mathfrak{so}}({2}^{n}),\qquad n\ge 4,\\ {{\mathfrak{a}}}_{10}^{\circ }(n)=\left\{\begin{array}{ll}{\mathfrak{su}}({2}^{n}),\qquad n\equiv \pm 1\,{\mathrm{mod}}\,\,3,\\ {{\mathfrak{a}}}_{10}(n),\qquad n\equiv 0\,{\mathrm{mod}}\,\,3,\end{array}\right.\\ \qquad\quad\cong \left\{\begin{array}{ll}{\mathfrak{su}}({2}^{n}),\qquad n\equiv \pm 1\,{\mathrm{mod}}\,\,3,\\ {\mathfrak{su}}{({2}^{n-2})}^{\oplus 4}, n\equiv 0\,{\mathrm{mod}}\,\,6,\\ {\mathfrak{su}}({2}^{n-1}),\quad n\equiv 3\,{\mathrm{mod}}\,\,6,\end{array}\right.\\ {{\mathfrak{a}}}_{11}^{\circ }(n)={\mathfrak{so}}({2}^{n}),\qquad n\ge 4,\\ {{\mathfrak{a}}}_{k}^{\circ }(n)={\mathfrak{su}}({2}^{n}),\,\,k=12,15,17,18,19,21,22,\\ {{\mathfrak{a}}}_{14}^{\circ }(n)\cong {\mathfrak{so}}{(2n)}^{\oplus 2},\\ {{\mathfrak{b}}}_{0}^{\circ }(n)={{\mathfrak{b}}}_{0}(n)\cong {\mathfrak{u}}{(1)}^{\oplus n},\\ {{\mathfrak{b}}}_{1}^{\circ }(n)\cong {\mathfrak{u}}{(1)}^{\oplus 2n},\\ {{\mathfrak{b}}}_{2}^{\circ }(n)\cong {\mathfrak{so}}({2}^{n}),\qquad n\ge 4,\\ {{\mathfrak{b}}}_{3}^{\circ }(n)={{\mathfrak{b}}}_{3}(n)\cong {\mathfrak{su}}{(2)}^{\oplus n},\\ {{\mathfrak{b}}}_{4}^{\circ }(n)={\mathfrak{su}}({2}^{n}).\end{array}$$

The proof of this theorem is given in SM C VIII. The proof strategy is different from that of Theorem I.1 because, unlike the open case, the periodic Lie algebras ${{\mathfrak{a}}}_{k}^{\circ }(n)$ are not generated inductively from ${{\mathfrak{a}}}_{k}^{\circ }(n-1)$. Instead, we use that ${{\mathfrak{a}}}_{k}^{\circ }(n)$ is generated from ${{\mathfrak{a}}}_{k}(n)$ and its cyclic shifts, and we utilize the explicit description ${{\mathfrak{a}}}_{k}(n)={{\mathfrak{g}}}_{k}{(n)}^{{\theta }_{k}}$ (see SM C VI for more details).

### Permutation-invariant subalgebras

The strategies employed for periodic boundary conditions can also be used to classify the DLAs in the case where the Hamiltonian is defined on a complete graph, so that each spin is connected to each other spin via 2-local interactions given by Pauli strings.

In more detail, we define a *DLA of* ${{\mathfrak{a}}}^{\pi }$-*type* as a Lie algebra of the form ${{\mathfrak{a}}}^{\pi }(n)={\langle {{\mathcal{A}}}^{\pi }(n)\rangle }_{{\rm{Lie}}}$ for some generating set of 2-site Pauli strings ${\mathcal{A}}\subseteq {\{X,Y,Z\}}^{\otimes 2}$, where

9 $${{\mathcal{A}}}^{\pi }(n)=\{{A}_{i}{B}_{j}\,| \,AB\in {\mathcal{A}},\,1\le i\,\ne\, j\le n\}.$$

A ${{\mathfrak{b}}}^{\pi }$-*type DLA* is one that cannot be expressed as ${{\mathfrak{a}}}^{\pi }$-type and has the form ${{\mathfrak{b}}}^{\pi }(n)={\langle {{\mathcal{A}}}^{\pi }(n)\cup {\mathcal{B}}(n)\rangle }_{{\rm{Lie}}}$ for some ${\mathcal{A}}\subseteq {\{X,Y,Z\}}^{\otimes 2}$, a non-empty set of Paulis ${\mathcal{B}}\subseteq \{X,Y,Z\}$, and ${\mathcal{B}}(n)$ given by (7).

#### Theorem 1.3

(Classification of Permutation Invariant DLAs). For *n* ≥ 3, every dynamical Lie algebra of type ${{\mathfrak{a}}}^{\pi }$ or ${{\mathfrak{b}}}^{\pi }$ is isomorphic to one of the following:

$$\begin{array}{ll}{{\mathfrak{a}}}_{k}^{\pi }(n)={{\mathfrak{a}}}_{k}(n),\qquad k=7,16,20,22,\\ {{\mathfrak{a}}}_{0}^{\pi }(n)\cong {\mathfrak{u}}{(1)}^{\oplus n(n-1)/2},\\ {{\mathfrak{a}}}_{2}^{\pi }(n)={\mathfrak{so}}{({2}^{n})}^{Z\cdots Z}\cong {\mathfrak{so}}{({2}^{n-1})}^{\oplus 2},\\ {{\mathfrak{a}}}_{4}^{\pi }(n)={{\mathfrak{a}}}_{7}(n)\cong \left\{\begin{array}{ll}{\mathfrak{su}}({2}^{n-1}),\qquad n\,\,{\rm{odd}},\\ {\mathfrak{su}}{({2}^{n-2})}^{\oplus 4},\quad n\ge 4\,\,{\rm{even}},\end{array}\right.\\ {{\mathfrak{a}}}_{6}^{\pi }(n)={{\mathfrak{a}}}_{20}(n)\cong {{\mathfrak{a}}}_{14}^{\pi }(n)\cong {\mathfrak{su}}{({2}^{n-1})}^{\oplus 2},\\ {{\mathfrak{b}}}_{0}^{\pi }(n)={{\mathfrak{b}}}_{0}(n)\cong {\mathfrak{u}}{(1)}^{\oplus n},\\ {{\mathfrak{b}}}_{1}^{\pi }(n)\cong {\mathfrak{u}}{(1)}^{\oplus n(n+1)/2},\\ {{\mathfrak{b}}}_{3}^{\pi }(n)={{\mathfrak{b}}}_{3}(n)\cong {\mathfrak{su}}{(2)}^{\oplus n}.\end{array}$$

The proof of this theorem is given in SM C IX.

## Discussion

In the previous section, we have classified the dynamical Lie algebras generated by the Pauli terms of 2-local spin chain Hamiltonians, both for open and closed boundary conditions and on complete graphs. To set some context for the reader, we present a few particular situations that come from variational quantum computing, quantum control, and physical systems, where dynamical Lie algebras arise and play an important role.

A quantum circuit can be described as a product of unitaries *U* = ∏_*k*_*U*_*k*_. Typically, the quantum circuit *U* acts on a multi-qubit state, whereas the gates *U*_*k*_ only act on single or two qubit subsystems, i.e., we can write ${U}_{k}={e}^{{a}_{k}}$ where *a*_*k*_ is a 1- or 2-local operator. For a set of generators ${\mathcal{A}}=\{{a}_{k}\}$ with a corresponding DLA ${\mathfrak{g}}={\langle {\mathcal{A}}\rangle }_{{\rm{Lie}}}$, we have the Lie group

10 $${e}^{{\mathfrak{g}}}=\{{e}^{{a}_{{k}_{1}}{t}_{1}}{e}^{{a}_{{k}_{2}}{t}_{2}}\cdots {e}^{{a}_{{k}_{r}}{t}_{r}}\,\left\vert \right.\,{t}_{i}\in {\mathbb{R}},\,{a}_{{k}_{i}}\in {\mathcal{A}}\}.$$

In other words, any element in the Lie group ${e}^{{\mathfrak{g}}}$ generated by the DLA can be reached by a finite product of unitaries in that group (see^45^, Corollary 3.2.6). In quantum computing, if ${e}^{{\mathfrak{g}}}={\rm{SU}}({2}^{n})$, then the gate set $\{{e}^{{a}_{k}}\}$ is called *universal*^47^. It is known that almost any combination of unitaries is universal^48,49^. However, we can make specific choices for the generators {*a*_*k*_} that correspond to a non-universal gate set, which instead will generate a proper subgroup of SU(2^*n*^). This is especially relevant for a class of quantum algorithms called variational quantum algorithms^50,51^.

If limited to 1-dimensional topology, the generators in our classification will produce a circuit that is an element of the Lie group ${e}^{{\mathfrak{g}}}$. This notion can be used to construct specific quantum algorithms that always act within a subgroup of SU(2^*n*^). Here, one considers a circuit that consists of parameterized gates,

$$U({\boldsymbol{\theta }})={U}_{1}({\theta }_{1}){U}_{2}({\theta }_{2})\cdots {U}_{K}({\theta }_{K}),\quad {U}_{k}({\theta }_{k})={e}^{{\theta }_{k}{a}_{k}}.$$

The gate parameters ***θ*** = (*θ*_1_, …, *θ*_*K*_) are real parameters that are optimized with a classical optimization routine to minimize a scalar cost function. A widely used example of such an optimization is the Variational Quantum Eigensolver algorithm (VQE)^52^, which has a cost function given by

11 $$C({\boldsymbol{\theta }})={\rm{Tr}}[U({\boldsymbol{\theta }}){\rho }_{0}{U}^{\dagger }({\boldsymbol{\theta }}){H}_{c}],$$

where *H*_*c*_ is a Hermitian operator and ${\rho }_{0}=\vert {\psi }_{0}\rangle \langle {\psi }_{0}\vert$ is the initial state of the system. Crucial to the success of this algorithms is the choice of a circuit ansatz *U*(***θ***) and the properties of the cost function (11).

A large class of variational circuits consist of *L* repeating layers of unitary blocks^39,53–60^, each with its own set of parameters:

$$U({\boldsymbol{\theta }})=\mathop{\prod }\limits_{l=1}^{L}\left(\mathop{\prod }\limits_{k=1}^{K}{U}_{k}({\theta }_{k}^{(l)})\right).$$

Below, we give some examples of these circuits and how our classification relates to them.

### Example II.1

*Hamiltonian Variational Ansatz*. The Hamiltonian Variational Ansatz circuit is obtained by Trotterizing the exponential of a Hamiltonian^55,56^. Consider the Hamiltonian

$${H}_{XY}=\mathop{\sum }\limits_{i=1}^{n-1}{X}_{i}{Y}_{i+1},$$

which has ${{\mathfrak{a}}}_{1}(n)$ as its DLA. Exponentiation of *H* and the application of the Trotter–Suziki formula then gives:

$$U({\boldsymbol{\theta }})=\mathop{\prod }\limits_{l=1}^{L}\left(\prod _{{\rm{even}}\,k}{e}^{i{\theta }_{k}^{(l)}{X}_{k}{Y}_{k+1}}\prod _{{\rm{odd}}\,k}{e}^{i{\theta }_{k}^{(l)}{X}_{k}{Y}_{k+1}}\right),$$

where we grouped the odd and even terms together due to the structure imposed by the 1- and 2-qubit gates available on the quantum computer. Due to (10) and the knowledge that ${{\mathfrak{a}}}_{1}(n)\cong {\mathfrak{so}}(n)$, we know that the above circuit must be a parameterization of a unitary operator *U*(***θ***) ∈ SO(*n*).

Similarly, we can take the DLA ${{\mathfrak{a}}}_{9}(n)$ with generators {*XY*, *XZ*}, which gives a circuit within Sp(2^*n*−2^):

$$\begin{array}{ll}U({\boldsymbol{\theta }},{\boldsymbol{\phi }})=\mathop{\prod }\limits_{l=1}^{L}\left(\mathop{\prod }\limits_{{\rm{even}}\,k}{e}^{i{\theta }_{k}^{(l)}{X}_{k}{Y}_{k+1}}\mathop{\prod }\limits_{{\rm{odd}}\,k}{e}^{i{\theta }_{k}^{(l)}{X}_{k}{Y}_{k+1}}\right.\\ \qquad\quad\left.\times \mathop{\prod }\limits_{{\rm{even}}\,k}{e}^{i{\phi }_{k}^{(l)}{X}_{k}{Z}_{k+1}}\mathop{\prod }\limits_{{\rm{odd}}\,k}{e}^{i{\phi }_{k}^{(l)}{X}_{k}{Z}_{k+1}}\right).\end{array}$$

**Fig. 3:**
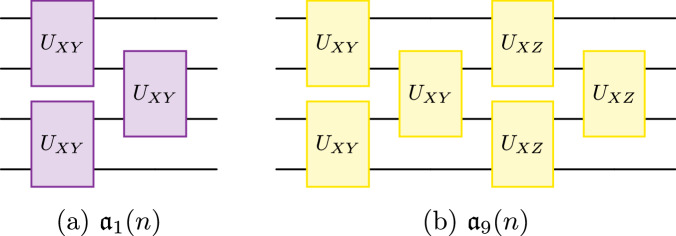
Examples of brick layer circuits that fall within our classification. **a** Variational ansatz circuit for the Hamiltonian ${H}_{XY}=\mathop{\sum }\nolimits_{i = 1}^{n-1}{X}_{i}{Y}_{i+1}$, which parameterizes an element of the group SO(*n*). **b** Variational ansatz that parameterizes a unitary in Sp(2^*n*−2^) via products of unitaries generated by terms in ${{\mathfrak{a}}}_{9}(n)$. We note that these types of brick-layer circuits often show up in the tensor network literature on quantum compilation^111,112^.

We illustrate these circuits schematically in Fig. 3.

### Example II.2

**Fig. 4:**
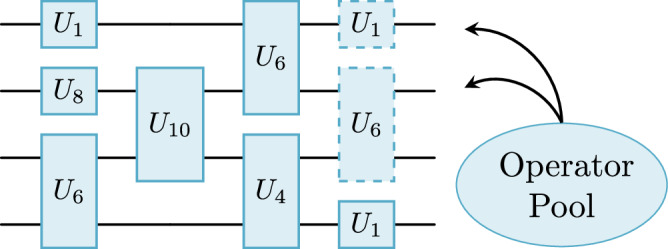
ADAPT-VQE circuit growing heuristic. We consider a generator pool in our classification and dynamically grow the circuit.

*Adapt-VQE*. In ADAPT-VQE, one dynamically grows the circuit using a predetermined operator pool, so that each gate lowers the cost function by the largest amount^61^. This class of dynamical circuit ansätze can be understood as a Riemannian gradient flow over a specific subgroup^62^. This heuristic is popular in quantum chemistry for circuit design, where specific operator pools are considered that are tailored to fermionic Hamiltonians^63–65^. The operator pool can be seen as a set of generators, with a corresponding DLA (see Fig. 4). In the context of our classification, we can thus determine the resulting subgroup of the dynamically grown circuit ansatz based on the generators in the operator pool.

### Example II.3

**Fig. 5:**
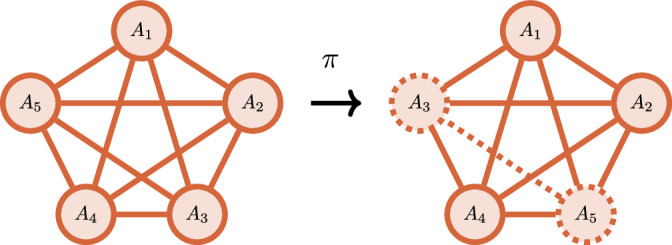
Permutation-invariant topology. Permuting sites leaves the Hamiltonian invariant.

*Permutation-invariant circuits*. Instead of a 1-dimensional topology, one can consider a Hamiltonian with a fully connected topology (see Fig. 5); for example,

$$H=\sum _{1\le i\ne j\le n}{X}_{i}{Y}_{j}.$$

This topology is common in ion trap quantum computers^66^ and also shows up in the context of quantum Boltzmann machines^67,68^, which are the quantum equivalent of the Sherrington–Kirkpatrick model with tunable parameters^69^. Closely related are the so-called permutation-equivariant circuits, which consist of parameterized blocks of unitaries that are permutation invariant^70^. These circuit ansätze were shown to be powerful quantum machine learning models for permutation-invariant data sets. Theorem I.3 provides a classification of DLAs for these types of ansätze.

A hurdle in minimizing a cost function of the form (11) are so-called *barren plateaus*^32^, which are flat areas in the cost landscape of a variational quantum algorithm. When barren plateaus are present, the variance of gradients with respect to the gate parameters will decay, on average, exponentially as a function of system size. Hence, obtaining accurate estimates quickly becomes intractable due to the large number of shots required. There is a variety of different setups in which barren plateaus occur^30,32,33,71–74^. To mitigate this problem, several recent works are aimed at finding ways to avoid the regions where optimization is hard^75–81^.

The relevance of our classification for barren plateaus stems from the conjecture of^31^, which states that the variance of the gradients of gate parameters is inversely proportional to the dimension of the DLA ${\mathfrak{g}}$ of the circuit:

$${\rm{Var}}[{\partial }_{k}C({\boldsymbol{\theta }})]\propto \frac{1}{\dim {\mathfrak{g}}}.$$

There are some subtleties involved in this conjecture, such as the locality of the cost function and the choice of initial state, which are discussed in refs. ^31,82^. In the common case where ${H}_{c}\in i{\mathfrak{g}}$, an exact formula for the variance was obtained independently in refs. ^82,83^, which in particular refines and proves the above conjecture. This formula was interpreted in ref. ^82^ in terms of the ${\mathfrak{g}}$-*purity*^84,85^ of the initial state *ρ*_0_ and the observable *H*_*c*_, underscoring again the crucial role of the DLA.

As an illustration, we compare the barren plateau behavior for two of our Lie algebras, ${{\mathfrak{a}}}_{5}^{\circ }(n)$ and ${{\mathfrak{a}}}_{14}(n)$, whose dimensions scale exponentially and polynomially with *n*, respectively. We consider the cost function (11) with *H*_*c*_ = *Z*_1_*Z*_2_ and ${\rho }_{0}=\left\vert 0\right\rangle {\left\langle 0\right\vert }^{\otimes n}$. The circuit ansatz *U*(***θ***) consists of unitaries generated by generators in our classification. To observe the barren plateau effect, we take the derivative of the cost function with respect to the first parameter in the first layer of the circuit, ${\theta }_{1}^{(1)}$.

**Fig. 6:**
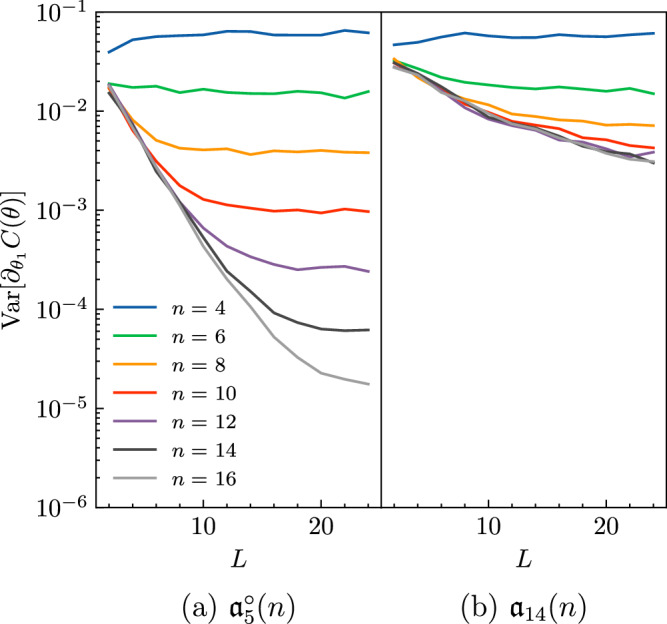
Barren plateaus in variational quantum circuits. We calculate the variance of 1000 randomly initialized circuits with ***θ*** sampled uniformly in [0, *π*]. **a** The Lie algebra ${{\mathfrak{a}}}_{5}^{\circ }(n)$ is isomorphic to ${\mathfrak{so}}({2}^{n})$, ${\mathfrak{so}}{({2}^{n-2})}^{\oplus 4}$ or ${\mathfrak{sp}}({2}^{n-2})$ depending on *n*; hence we expect exponentially decaying gradients for all *n*. This is confirmed in the figure above, since for a linear increase in *n*, we see an order of magnitude decrease in the gradient variances. **b** Since ${{\mathfrak{a}}}_{14}(n)\cong {\mathfrak{so}}(2n)$, we find polynomially decaying gradients as a function of system size.

In Fig. 6, we then observe the expected gradient decay as a function of the system size for an exponentially scaling DLA and a polynomially scaling DLA. In particular, in Fig. 6a we consider the circuit generated by $H=\mathop{\sum }\nolimits_{i = 1}^{n-1}({X}_{i}{Y}_{i+1}+{Y}_{i}{Z}_{i+1})+{X}_{n}{Y}_{1}+{Y}_{n}{Z}_{1}$ with periodic boundary conditions, whose DLA ${{\mathfrak{a}}}_{5}^{\circ }(n)$ is isomorphic to ${\mathfrak{so}}({2}^{n})$, ${\mathfrak{so}}{({2}^{n-2})}^{\oplus 4}$ or ${\mathfrak{sp}}({2}^{n-2})$ depending on *n* (see Theorem I.2). Since $\dim {{\mathfrak{a}}}_{5}^{\circ }(n)=O({4}^{n})$, we expect the gradients to decay exponentially. Similarly, in Fig. 6b we consider the circuit generated by ${{\mathfrak{a}}}_{14}(n)\cong {\mathfrak{so}}(2n)$, which is described by the Hamiltonian $H=\mathop{\sum }\nolimits_{i = 1}^{n-1}({X}_{i}{X}_{i+1}+{Y}_{i}{Y}_{i+1}+{X}_{i}{Y}_{i+1})$. Here, we have $\dim {{\mathfrak{a}}}_{14}(n)=O({n}^{2})$; hence, we expect the decay of gradients to be polynomial with respect to the system size.

According to Corollary I.1, the only circuits free from barren plateaus generated by Hamiltonians in our classification, which are not composed of only 1-qubit gates, have to be composed of ${\mathfrak{so}}$-type, since these are the only polynomially scaling DLAs in our classification.

Modern neural networks used in deep learning tend to have many more parameters than available data points, but are both easy to optimize and generalize well to unseen data in practice^86^. This phenomenon is known as *overparameterization*. In variational quantum computing, a similar effect has been observed^27,28,87^, where deep variational quantum circuits tend to have favorable optimization properties. Recent works that have made progress in theoretically understanding this effect in quantum circuits can be connected to the DLA generated by the circuit ansätze used^29,30^. In particular, in^30^, the dimension of the DLA can be used to analyze the Hessian around the global minimum of a typical variational quantum eigensolver cost function^52^. Additionally, the authors find that the critical number of parameters needed to overparameterize a variational quantum circuit can be directly linked to the dimension of the associated DLA. In^29^, the authors study the optimization dynamics of overparameterized quantum circuit as perturbations of Riemannian gradient flows^88^. The size of the DLA (defined as the effective dimension in^29^) allows one to bound the number of parameters required to reach the overparameterization regime.

Corollary I.1 tells us that for quantum circuits constructed from the Pauli generators of spin chains, there are only DLAs whose dimension scales as *O*(*n*), *O*(*n*^2^) and *O*(4^*n*^). Consequently, the linearly and quadratically scaling DLAs are expected to overparameterize with a non-exponential number of parameters. Additionally, the quadratically scaling DLAs in our classification correspond to free fermion models, whose dynamics can be simulated efficiently if *ρ*_0_ is an eigenstate of *H*_*c*_. However, choosing *ρ*_0_ to be an arbitrary quantum state will still be intractable to simulate classically.

As discussed in ref. ^29^, a requirement for overparameterzation is that the initial state has non-vanishing overlap with the ground state. Similarly, in ref. ^89^, it is shown that choosing the initial state in the right symmetry sector is crucial for the quality of the optimization. We highlight this importance in one of the numerical examples, where we choose an initial state that prevents overparameterization from occurring for an odd number of sites.

**Fig. 7:**
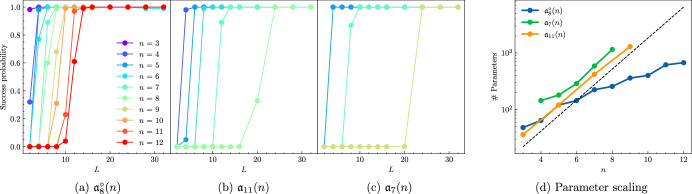
Overparameterization of variational quantum circuits. We plot the success probability of reaching a state with ∣*E*_0_ − *C*(***θ***)∣ < 5 × 10^−4^ as a function of the circuit depth *L*, where *E*_0_ is the lowest energy of the cost Hamiltonian *H*_*c*_. These results were obtained by averaging 100 random instances for each *L* and *N*. The error bars indicate one standard deviation *σ*. A single random instance consists of 3000 optimization steps of the Adam optimizer^113^ with learning rate *η* = 10^−2^. A handful of instances converge to solutions that are further than 5*σ* from the mean and these outliers are therefore not included in the final plot. **a** The TFIM on a ring has DLA ${{\mathfrak{a}}}_{8}^{\circ }(n)\cong {\mathfrak{so}}{(2n)}^{\oplus 2}$, whose dimension scales quadratically. We see that for a moderate circuit depth, the probability of success goes to 1. **b** Since the DLA ${{\mathfrak{a}}}_{11}(n)\cong {\mathfrak{so}}({2}^{n})$, we expect that overparameterization occurs at depths that are exponential in the system size. Although this is not immediately clear in here, we see in (**d**) that the number of parameters indeed scales exponentially in *n*. **c** For the Heisenberg chain, which has an exponentially-scaling DLA ${{\mathfrak{a}}}_{7}(n)$, the choice of initial state ${\rho }_{0}=\left\vert 0\right\rangle {\left\langle 0\right\vert }^{\otimes n}$ prevents overparameterization from occurring for odd *n*. **d** If we set the threshold for overparameterization to be a success probability of 0.99, we can plot the required number of parameters to reach this threshold. We see that ${{\mathfrak{a}}}_{11}(n)$ and ${{\mathfrak{a}}}_{7}(n)$ require an exponentially scaling number of parameters, whereas ${{\mathfrak{a}}}_{8}^{\circ }(n)$ only requires a polynomial number. The dashed line is a guiding line that indicates *O*(4^*n*^) scaling.

In Fig. 7, we illustrate the overparametrization phenomenon for three examples in our classification. In particular, in Fig. 7a, we consider the TFIM on a ring, which is given by the Hamiltonian ${H}_{c}=\mathop{\sum }\nolimits_{i = 1}^{n}({Z}_{i}{Z}_{i+1}+{X}_{i})$ where *Z*_*n*+1_ ≔ *Z*_1_. The corresponding DLA is given by ${{\mathfrak{a}}}_{8}^{\circ }(n)\cong {\mathfrak{so}}{(2n)}^{\oplus 2}$, whose dimension scales quadratically in *n*. We take the Hamiltonian Variational Ansatz of *H*_*c*_ on even and odd qubits as a circuit ansatz, and take the initial state to be ${\rho }_{0}=\left\vert +\right\rangle {\left\langle +\right\vert }^{\otimes n}$. We observe that the cost landscape quickly becomes favorable, resulting in almost guaranteed convergence to the lowest energy state.

In Fig. 7b, we take the DLA ${{\mathfrak{a}}}_{11}(n)\cong {\mathfrak{so}}({2}^{n})$, and a Hamiltonian ${H}_{c}\in {\mathfrak{so}}({2}^{n})$ given by a random skew-symmetric 2^*n*^ × 2^*n*^ real matrix. The circuit consists of unitaries generated by the generators {*XY*, *YX*, *YZ*} of ${{\mathfrak{a}}}_{11}$ placed on even and odd adjacent qubits, and we take ${\rho }_{0}=\left\vert 0\right\rangle {\left\langle 0\right\vert }^{\otimes n}$. It now takes much deeper circuits to reach the same success probabilities as in Fig. 7a, which is due to the exponential scaling of the DLA.

Finally, in Fig. 7c, we consider ${{\mathfrak{a}}}_{7}(n)$, which corresponds to the Heisenberg chain with ${H}_{c}=\mathop{\sum }\nolimits_{i = 1}^{n-1}({X}_{i}{X}_{i+1}+{Y}_{i}{Y}_{i+1}+{Z}_{i}{Z}_{i+1})$. The circuit is again the Hamiltonian Variational Ansatz of *H*_*c*_, and ${\rho }_{0}=\left\vert 0\right\rangle {\left\langle 0\right\vert }^{\otimes n}$. This choice of an initial state only works for an odd number *n* of qubits, while it fails to produce the overparameterization phenomenon for even *n*, leading to a success probability of 0 (not plotted). Instead, for even *n*, the optimization of deep circuits gets stuck in a local minimum. We still observe the exponential scaling of the number of parameters, in accordance with the scaling of the dimension of ${{\mathfrak{a}}}_{7}(n)$, which is *O*(4^*n*^).

In quantum control, one is interested in performing a specific unitary evolution by controlling individual terms in a Hamiltonian acting on a small number of qubits. In order to create any unitary, one requires the system to be *controllable*, which corresponds to the DLA being equal to ${\mathfrak{su}}({2}^{n})$. The conditions for complete controllability are known^35^, and it is in principle easy to create a controllable system since any real simple Lie algebra can be generated from 2 elements^90^. If the DLA is a proper subalgebra of ${\mathfrak{su}}({2}^{n})$, we say that the system is *uncontrollable*. Note that this includes simple Lie algebras like ${\mathfrak{so}}$ and ${\mathfrak{sp}}$^36^. Uncontrollable systems can arise when there are conserved quantities or symmetries in the physical system one is trying to control. Note that, due to Proposition I.1, the DLA must split into a direct sum of simple Lie algebras and a center. If this decomposition has the form ${\mathfrak{su}}({N}_{1})\oplus \cdots \oplus {\mathfrak{su}}({N}_{k})$ where *N*_1_ + ⋯ + *N*_*k*_ = 2^*n*^, then we say that the system is *subspace controllable*^45^, Sect. 4.3.3.

We can contextualize our classification in terms of these definitions. For example, we know that ${{\mathfrak{a}}}_{12}(n)$ will produce a controllable quantum system for *n* ≥ 4 since this DLA is equal to ${\mathfrak{su}}({2}^{n})$. Similarly, since ${{\mathfrak{a}}}_{1}(n)\cong {\mathfrak{so}}(n)$, we know that it is uncontrollable. Finally, there are many examples of uncontrollable systems that consist of direct sums of ${\mathfrak{su}}$ blocks and are subspace controllable. For instance, ${{\mathfrak{a}}}_{3}^{\circ }(n)$ for odd *n* produces a DLA of the form ${\mathfrak{su}}{({2}^{n-1})}^{\oplus 2}$. In addition to the notion of controllability of spin systems, we can ask what other types of systems we can simulate with our spin chains, e.g., fermionic or bosonic systems. This question was originally explored for Hamiltonians on cubic lattices with translation symmetry^91,92^. In particular, the dynamics of quadratic fermionic Hamiltonians is described by DLAs of the ${\mathfrak{so}}(2n)$ or ${\mathfrak{so}}(2n+1)$ type, which show up in our classification as ${{\mathfrak{a}}}_{14}(n)$ and ${{\mathfrak{a}}}_{8}(n)$. Similarly, the dynamics of a bosonic quadratic Hamiltonian with *n* modes is related to a symplectic DLA^38^, which we can identify with ${{\mathfrak{a}}}_{5}(n)$ for *n* ≡ 3 mod 6.

Our classification of Lie algebras arising in spin chains has bearing on other areas of physics and quantum simulation. The most direct connection is that we have established sets of equivalent models, some of which are traditional spin models^93,94^ studied in physics, while others are new (cf. Table S.II). The integrability^24,25,95^, dynamical Lie algebra, and symmetry of 1-dimensional spin systems remains an active area of research, and our result provides a database of models where desired properties can be selected or different hypotheses tested.

The various properties of physical systems typically arise from the exact Hamiltonian. As a straightforward example, we can consider the transverse-field Ising model; whether this model is in the paramagnetic or ferromagnetic phase depends on the relative strengths of the nearest neighbor coupling and applied magnetic field. In this work, we have neglected these parameters in the Hamiltonian and solely focused on the presence (or absence) of terms in the Hamiltonian. This coarsening of the problem hides much of the critical detail. However, there is still a remarkable amount of information that can be gleaned from this admittedly coarse view.

One example of this is the observation that Hamiltonians with polynomially-scaling dynamical Lie algebras belong to a special class of integrable models, which can be simulated efficiently with Lie algebra-based algorithms^85,96^. Additionally, the dynamics of such models can be simulated in less time than the duration of the desired evolution through a process called fast-forwarding^97^. These algorithms have recently been rediscovered for the purposes of simulating specific classes of quantum circuits^34,83^. Interestingly, the number of polynomially-scaling algebras in our classification is relatively small (${{\mathfrak{a}}}_{1},{{\mathfrak{a}}}_{2},{{\mathfrak{a}}}_{4},{{\mathfrak{a}}}_{8},{{\mathfrak{a}}}_{14},{{\mathfrak{a}}}_{1}^{\circ },{{\mathfrak{a}}}_{2}^{\circ },{{\mathfrak{a}}}_{4}^{\circ },{{\mathfrak{a}}}_{8}^{\circ },{{\mathfrak{a}}}_{14}^{\circ }$), and they are all of the ${\mathfrak{so}}$-type. For example, the TFIM model, which is known to be integrable, arises in our classification as ${{\mathfrak{a}}}_{8}(n)\cong {\mathfrak{so}}(2n-1)$, which is polynomial in size.

The polynomially scaling algebras in principle come with a “maximal set of independent commuting quantum operators”^98^, which enables the integration in the first place. Unfortunately, our method does not capture these because the conserved quantities are not single Pauli strings. However, global symmetries are preserved for some of the models; these include ${{\mathbb{Z}}}_{2}$ (spin flip), SU(2) (global spin rotation) and U(1) (global phase rotation).

One particular property of note is the presence of *non-commuting charges* which describe *non-Abelian symmetries*—that is, elements of the stabilizer of the DLA that do not commute with each other. These are found in ${{\mathfrak{a}}}_{8}(n),{{\mathfrak{a}}}_{9}(n)$ for all *n*, and in ${{\mathfrak{a}}}_{2}(n)-{{\mathfrak{a}}}_{7}(n),{{\mathfrak{a}}}_{10}(n)$ for odd *n* only (see SM C IV for a list of stabilizers and their centers). We emphasize that the non-commuting charges we find are intensive, as opposed to extensive. The latter consist of a sum of terms that grows with the system size, which can be related to a wide range of quantum effects in thermodynamics (see ref. ^99^ for a review). Extensive non-commuting charges have been studied in the context of bond algebras to understand thermalization and quantum many body scars^14,100,101^, hence our classification could potentially be used in this context. Notably, the presence of non-commuting charges complicates questions regarding thermalization. Depending on the context, they either help thermalization (e.g. by increasing entanglement entropy^102^) or hinder it (e.g. by invalidating the Eigenstate Thermalization Hypothesis^103^). Perhaps more interestingly within the context of quantum computing, non-commuting charges couple the dynamics between different irreducible representations of the charges, severely limiting the unitaries that can be implemented^104^. The DLA can also be used in the path integral formulation of quantum mechanics to study many-body systems^105^.

A final point is the appearance of symplectic Lie algebras, which are not as common as the orthogonal or unitary types. Here they appear from an AIII Cartan decomposition of a larger Lie algebra; in applications, they come up in the preparation of bosonic quantum states^92^, photonics^106^ and Clifford circuits/error correction^107^.

In conclusion, we have provided a classification of the *dynamical Lie algebras* (DLAs) generated by the Pauli terms of 2-local spin systems on a linear, circular or permutation invariant topology, and have discussed the relevance of this result in a variety of contexts. We have discovered several new examples beyond the standard Ising and Heisenberg models; thus increasing dramatically the number of explicit Hamiltonians available for theoretical investigations. It would be interesting to study in more detail the thermodynamic properties of these new Hamiltonians, and in particular to determine all of their symmetries, including the extensive non-commuting charges. This would require taking the coefficients of the Hamiltonian into account to refine the classification from the general form we consider here to a specific physical system. We hope that our classification can be used to inspire new quantum algorithms and allow researchers to identify the circuits that they use in practice with the Lie algebras in our classification to asses their optimization properties. Moreover, the methods that we have developed can be used to identify the DLA even in cases that fall outside of our classification.

In fact, we have been able to extend the classification presented in this work from 2-local 1-dimensional topologies to arbitrary *interaction graphs*^44^. The basis of these new results are the present results on complete graphs, which serve as an upper bound for the DLA of an arbitrary graph. Further extensions to 3-local Hamiltonians may be possible, but the size of the power set (2^63^ − 1) would have to be reduced beforehand to make a full enumeration tractable. Additionally, we would like to investigate how the coefficients of the Hamiltonian affect the DLA, which would require extending our method to the case where the generators are linear combinations of Pauli strings.

Another future direction would be to consider other types of systems. For example, instead of spin systems, we could consider fermionic or bosonic Hamiltonians. Such a classification already exists for nearest-neighbor, quadratic Hamiltonians on cubic lattices^92,108^, so this question would have to be explored in the context of non-cubic graphs.

## Methods

### The power sets

We consider all possible interaction sets ${\mathcal{A}}\subseteq {{\mathcal{P}}}_{2}$ not containing *I* ⊗ *I* and want to determine the Lie algebras ${\langle {\mathcal{A}}\rangle }_{{\rm{Lie}}}$ generated by them. The case where ${\mathcal{A}}\subseteq {\{X,Y,Z\}}^{\otimes 2}$ contains only proper 2-qubit interactions without *I* acting on any qubit will be called of ${\mathfrak{a}}$-*type*. There are 9 Pauli strings in {*X*, *Y*, *Z*}^⊗2^; hence, we have 2^9^ − 1 = 511 non-empty subsets ${\mathcal{A}}$ of ${\mathfrak{a}}$-type.

**Fig. 8:**
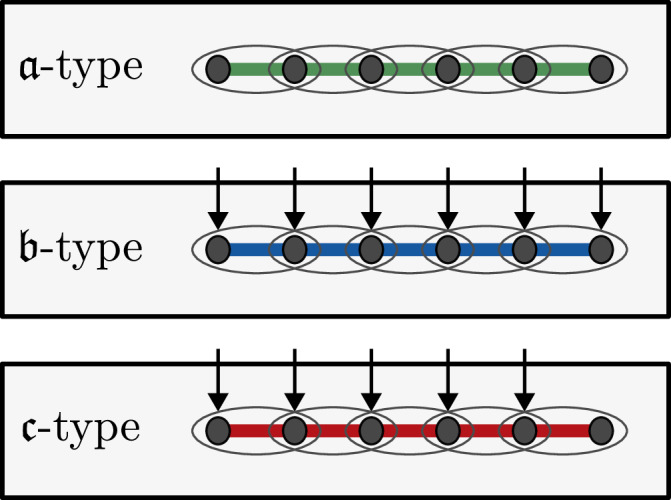
The three types of Lie algebras in the classification. The ${\mathfrak{a}}$-type Lie algebras can be generated only by nearest neighbor 2-Pauli interactions. The ${\mathfrak{b}}$-type Lie algebras are generated by nearest neighbor 2-Pauli interactions and 1-Pauli operators acting on every qubit; for ${\mathfrak{c}}$-type Lie algebras the 1-Pauli operators act differently on the boundary.

The remaining cases, in which some elements of ${\mathcal{A}}$ contain *I* acting on one qubit, will be called of ${\mathfrak{b}}$ and ${\mathfrak{c}}$-types. Note that if $a\otimes I\in {\mathcal{A}}$ for some *a* ∈ {*X*, *Y*, *Z*}, then the Hamiltonian (2) contains terms *a*_*k*_ for *k* = 1, …, *n* − 1. Similarly, if $I\otimes a\in {\mathcal{A}}$ for some *a* ∈ {*X*, *Y*, *Z*}, then (2) contains terms *a*_*k*_ for *k* = 2, …, *n*. As we want the 1-qubit Pauli operators to act on all sites, we will assume that if our interaction set ${\mathcal{A}}$ contains *a* ⊗ *I*, then it also contains *I* ⊗ *a* and vice versa. Such sets will be called of ${\mathfrak{b}}$-*type*, and everything else that is not of ${\mathfrak{a}}$ or ${\mathfrak{b}}$-type will be of ${\mathfrak{c}}$-*type*. Note that in the case of periodic boundary conditions every ${\mathfrak{c}}$-type generating set can be replaced by an equivalent ${\mathfrak{b}}$-type set, while for open boundary conditions ${\mathfrak{c}}$-type differs from ${\mathfrak{b}}$-type only on the first or last site in the chain (see Fig. 8).

In total, there are 2^15^ − 1 = 32767 non-empty subsets ${\mathcal{A}}$ of ${{\mathcal{P}}}_{2}\setminus \{I\otimes I\}$. We proceed by going through all of them and using Algorithm 1 (in the simplified form from Remark I.1) to perform the nested commutators in (1) in order to determine the Lie algebra ${\langle {\mathcal{A}}\rangle }_{{\rm{Lie}}}$. This procedure can be performed numerically with ease, and we provide the code to reproduce it at^109^. We store only the unique (up to equality as sets) subalgebras ${\langle {\mathcal{A}}\rangle }_{{\rm{Lie}}}$, and we obtain only 127 and 19 subalgebras of ${\mathfrak{su}}(4)$ of ${\mathfrak{a}}$-type and ${\mathfrak{b}}$-type, respectively. For completeness, these are listed explicitly in SM B II, together with 56 subalgebras of ${\mathfrak{c}}$-type.

#### Example III.1

Consider the generating sets:

$$\begin{array}{ll}{{\mathcal{A}}}_{1}\,=\,\{XY,XZ\},\\ {{\mathcal{A}}}_{2}\,=\,\{IX,XY\}.\end{array}$$

Note that as generating sets, ${{\mathcal{A}}}_{1}$ is of ${\mathfrak{a}}$-type and ${{\mathcal{A}}}_{2}$ is of ${\mathfrak{c}}$-type. After running Algorithm 1, we find:

$$\begin{array}{ll}{\langle {{\mathcal{A}}}_{1}\rangle }_{{\rm{Lie}}}\,=\,{\rm{span}}\{XY,XZ,IX\},\\ {\langle {{\mathcal{A}}}_{2}\rangle }_{{\rm{Lie}}}\,=\,{\rm{span}}\{IX,XY,XZ\}.\end{array}$$

We see that ${{\mathcal{A}}}_{1}$ and ${{\mathcal{A}}}_{2}$ generate the same Lie algebra; hence, this Lie algebra is counted among the ${\mathfrak{a}}$-type Lie algebras.

#### Example III.2

The set of ${\mathfrak{b}}$-type

$${\mathcal{A}}=\{XY,XI,IX\}$$

generates the Lie algebra

$$\begin{array}{r}{\langle {\mathcal{A}}\rangle }_{{\rm{Lie}}}={\rm{span}}\{XY,XZ,XI,IX\}.\end{array}$$

If we try to generate it from the ${\mathfrak{a}}$-type subset {*XY*, *XZ*}, we find the strictly smaller subalgebra

$$\begin{array}{r}{\langle {{XY,XZ}}\rangle}_{{\rm{Lie}}}={\rm{span}}\{XY,XZ,IX\}.\end{array}$$

Hence, ${\langle {\mathcal{A}}\rangle }_{{\rm{Lie}}}$ is indeed of ${\mathfrak{b}}$-type and not of ${\mathfrak{a}}$-type. This DLA is strictly larger that the DLA from Example III.1 in the case of open boundary conditions. However, for periodic boundary conditions, *X* will be placed on every site, and the DLA will become equal to the DLA generated by the set ${{\mathcal{A}}}_{2}=\{IX,XY\}$.

#### Example III.3

Consider the generating sets of ${\mathfrak{a}}$-type:

$$\begin{array}{ll}{{\mathcal{A}}}_{1}\,=\,\{XX,YY\},\\ {{\mathcal{A}}}_{2}\,=\,\{XX,YX\}.\end{array}$$

After running Algorithm 1, we find:

$$\begin{array}{ll}{\langle {{\mathcal{A}}}_{1}\rangle }_{{\rm{Lie}}}\,=\,{\rm{span}}\{XX,YY\},\\ {\langle {{\mathcal{A}}}_{2}\rangle }_{{\rm{Lie}}}\,=\,{\rm{span}}\{XX,YX,ZI\}.\end{array}$$

Hence, ${{\mathcal{A}}}_{1}$ and ${{\mathcal{A}}}_{2}$ generate distinct Lie algebras, both of ${\mathfrak{a}}$-type.

### Symmetries of the power sets

There are certain symmetries that can be exploited to reduce the number of subalgebras of ${\mathfrak{su}}(4)$ in the above power sets. To start, we note that the Pauli matrices satisfy the following algebraic relations:

$$[{\sigma }^{\alpha },{\sigma }^{\beta }]=2i\mathop{\sum }\limits_{\gamma =1}^{3}{\epsilon }_{\alpha \beta \gamma }{\sigma }^{\gamma },$$

where *ϵ*_*αβγ*_ is the Levi-Civita tensor and *α*, *β*, *γ* ∈ {1, 2, 3}, respectively (see SM A I for more details). We will ignore the factor 2*i*, since we only care about the linear span of nested commutators. Note that the above relation is independent of how we assign *X*, *Y*, *Z* to *σ*^*α*^. In other words, we can relabel the Paulis and retain the algebraic structure of the subalgebras, which together with ignoring the prefactors formally corresponds to an *S*_3_ permutation symmetry.

In addition to relabelling, we consider the exchange of location of the two Pauli terms, since the order of such terms is an arbitrary choice that does not impact the structure of the resulting Lie algebras. This location exchange corresponds to a ${{\mathbb{Z}}}_{2}$ symmetry. Hence, the symmetry group of the Pauli algebra for *n* = 2 is ${S}_{3}\times {{\mathbb{Z}}}_{2}$. Subalgebras of ${\mathfrak{su}}(4)$ that are in the same orbit of this symmetry group will be considered equivalent, which allows us to reduce the number of subalgebras significantly.

#### Example III.4

We have that {*XX*, *YZ*} ≡ {*YY*, *ZX*} are equivalent under relabeling *X* → *Y* → *Z* → *X*. On the other hand, {*XX*, *YZ*} and {*XX*, *YY*} are not equivalent.

In order to determine the orbits of the symmetry group ${S}_{3}\times {{\mathbb{Z}}}_{2}$ on the set of subalgebras of ${\mathfrak{su}}(4)$ listed in SM B II, we introduce their invariants *s*, *p*, *e*, *d*, defined as follows. These enumerate the number of single Paulis (such as *XI*) in the basis of the Lie algebra, the number of single Pauli pairs (such as *XI*, *IX*), the number of double equal Paulis (such as *XX*) and the number of double different Paulis (such as *XY*), respectively. Since all these quantities are invariant under the action of the symmetry group, two subalgebras are not equivalent if they have different invariants.

#### Example III.5

Consider the following bases of subalgebras and their invariants:

$$\begin{array}{lc}{{\mathcal{A}}}_{1}\,=\,\{ZZ,YX,XY\}\to (0,0,1,2),\\ {{\mathcal{A}}}_{2}\,=\,\{XX,YZ,ZY\}\to (0,0,1,2),\\ {{\mathcal{A}}}_{3}\,=\,\{YY,ZX,XZ\}\to (0,0,1,2).\end{array}$$

We see that ${{\mathcal{A}}}_{1}\equiv {{\mathcal{A}}}_{2}$ under *Z* *⇌* *X*. Similarly, ${{\mathcal{A}}}_{2}\equiv {{\mathcal{A}}}_{3}$ under *X* *⇌* *Y* and ${{\mathcal{A}}}_{3}\equiv {{\mathcal{A}}}_{1}$ under *Y* *⇌* *Z*.

#### Example III.6

Consider the following bases of subalgebras and their invariants:

$$\begin{array}{ll}{{\mathcal{A}}}_{1}\,=\,\{XX,XZ,IY\}\to (1,0,1,1),\\ {{\mathcal{A}}}_{2}\,=\,\{XY,XZ,IX\}\to (1,0,0,2).\end{array}$$

We see that ${{\mathcal{A}}}_{1}\not\equiv {{\mathcal{A}}}_{2}$, since they have different invariants.

#### Example III.7

Even though the two bases

$$\begin{array}{ll}{{\mathcal{A}}}_{1}\,=\,\{XY,YX\}\to (0,0,0,2),\\ {{\mathcal{A}}}_{2}\,=\,\{XY,YZ\}\to (0,0,0,2)\end{array}$$

have the same invariants, they are not equivalent under the symmetry group ${S}_{3}\times {{\mathbb{Z}}}_{2}$.

Carrying out this procedure exhaustively for the 127 and 19 subalgebras of ${\mathfrak{a}}$-type and ${\mathfrak{b}}$-type gives us 23 and 5 inequivalent Lie algebras, respectively. We denote these subalgebras by ${{\mathfrak{a}}}_{k}\,(0\le k\le 22)$ and ${{\mathfrak{b}}}_{l}\,(0\le l\le 4)$. For the full list of invariants, see Table S.I in the Supplemental Materials. In particular, it turns out that the only case in which the invariants (*s*, *p*, *e*, *d*) cannot distinguish inequivalent subalgebras is that presented in Example III.7.

By Proposition I.1, we can identify these subalgebras by inspection with direct sums of simple Lie algebras plus a center.

#### Example III.8

The set

$${\mathcal{A}}=\{XX,YY,ZZ,ZY\}$$

generates the Lie algebra

$$\begin{array}{rc}&{{\mathfrak{a}}}_{20}:= {\langle {\mathcal{A}}\rangle }_{{\rm{Lie}}}={\rm{span}}\{XX,YY,ZZ,YZ,ZY,XI,IX\}\\ &={\rm{span}}\{YY+ZZ,YZ+ZY,XI+IX\}\\ &\oplus {\rm{span}}\{YY-ZZ,YZ-ZY,XI-IX\}\oplus {\rm{span}}\{XX\}\\ &\cong {\mathfrak{su}}(2)\oplus {\mathfrak{su}}(2)\oplus {\mathfrak{u}}(1).\end{array}$$

At this point, we have reduced the number of possible DLAs significantly by taking into account the symmetries of the Pauli group. After that, we grow the DLA from 2 to *n* sites, as explained in Section “Growing the dynamical Lie algebras”.

### Sketch of the proof of Theorem I.1

The complete proof of Theorem I.1 is presented in the Supplemental Materials. Here is a brief sketch of the proof; we refer to SM C I for a more detailed outline. We divide the set of Lie algebras ${{\mathfrak{a}}}_{k}(n)$, ${{\mathfrak{b}}}_{l}(n)$ into three classes: linear, quadratic, and exponential, according to the anticipated growth of their dimension. The *linear* class consists of ${{\mathfrak{a}}}_{0}(n)$ and ${{\mathfrak{b}}}_{l}(n)$ with *l* = 0, 1, 3, and their treatment is obvious. The *quadratic* class contains ${{\mathfrak{a}}}_{k}(n)$ with *k* = 1, 2, 4, 8, 14. These Lie algebras are determined by using the frustration graphs of their generators in SM C III. For the *exponential* class, we first observe that ${{\mathfrak{b}}}_{2}(n)={{\mathfrak{a}}}_{9}(n)\oplus {\rm{span}}\{{X}_{1}\}$ and ${{\mathfrak{b}}}_{4}(n)={{\mathfrak{a}}}_{15}(n)\oplus {\rm{span}}\{{X}_{1}\}$. Next, we identify the cases when ${{\mathfrak{a}}}_{k}(n)={\mathfrak{su}}({2}^{n})$; see SM B IV for details. We also find isomorphisms that are obtained by relabeling of the Pauli matrices among some of the algebras (SM C II).

The **strategy** in the remaining exponential cases is as follows.

1. For each of our Lie subalgebras ${\mathfrak{s}}={{\mathfrak{a}}}_{k}(n)\subseteq {\mathfrak{su}}({2}^{n})$, we find its *stabilizer* ${\rm{St}}({\mathfrak{s}})$, which is defined as the set of all Pauli strings $\in {{\mathcal{P}}}_{n}$ that commute with every element of ${\mathfrak{s}}$. This can be done explicitly, because the stabilizer is determined only from the generators of ${\mathfrak{s}}$ (see Proposition C.3).
2. By definition, ${\mathfrak{s}}$ commutes with all elements of its stabilizer ${\rm{St}}({\mathfrak{s}})$; hence, it is contained in the *centralizer* of ${\rm{St}}({\mathfrak{s}})$ in ${\mathfrak{su}}({2}^{n})$, which we denote ${\mathfrak{su}}{({2}^{n})}^{{\rm{St}}({\mathfrak{s}})}$. We can reduce the Lie subalgebra ${\mathfrak{su}}{({2}^{n})}^{{\rm{St}}({\mathfrak{s}})}$ further by factoring all elements of the center of ${\rm{St}}({\mathfrak{s}})$, which will become central in it, because we have shown that ${\mathfrak{s}}$ has a trivial center (Lemma C.12). This results in a Lie algebra denoted ${{\mathfrak{g}}}_{k}(n)$ when ${\mathfrak{s}}={{\mathfrak{a}}}_{k}(n)$.
3. By the above construction, we have ${{\mathfrak{a}}}_{k}(n)\subseteq {{\mathfrak{g}}}_{k}(n)$. In the case of associative algebras, we would get equality due to (a finite-dimensional version of) von Neumann’s Double Commutant Theorem (see e.g.^110^, Theorem 6.2.5). However, in the Lie case, we might have a strict inclusion. We improve the upper bounds for ${{\mathfrak{a}}}_{k}(n)$ by finding *involutions* *θ*_*k*_ of ${{\mathfrak{g}}}_{k}(n)$ such that all elements of ${{\mathfrak{a}}}_{k}(n)$ are fixed under *θ*_*k*_. The last condition can be checked only on the generators of ${{\mathfrak{a}}}_{k}(n)$ (see SM C V).
4. We prove by induction on *n* that the upper bound is exact, that is ${{\mathfrak{a}}}_{k}(n)={{\mathfrak{g}}}_{k}{(n)}^{{\theta }_{k}}$ (see SM C VI). First we note that both ${{\mathfrak{a}}}_{k}(n)$ and ${{\mathfrak{g}}}_{k}{(n)}^{{\theta }_{k}}$ are linearly spanned by the Pauli strings contained in them. We start with an arbitrary Pauli string $a\in i{{\mathcal{P}}}_{n}\cap {{\mathfrak{g}}}_{k}{(n)}^{{\theta }_{k}}$ and want to show that it is in ${{\mathfrak{a}}}_{k}(n)$. The main idea is to use suitable commutators of *a* with elements of ${{\mathfrak{a}}}_{k}(n)$ to produce a Pauli string $b\in i{{\mathcal{P}}}_{n}\cap {{\mathfrak{g}}}_{k}{(n)}^{{\theta }_{k}}$ with *I* in one of its positions. Erasing the *I* in *b* gives an element of ${{\mathfrak{g}}}_{k}{(n-1)}^{{\theta }_{k}}$, which by induction is in ${{\mathfrak{a}}}_{k}(n-1)$.
5. Finally, we identify the Lie algebras ${{\mathfrak{g}}}_{k}{(n)}^{{\theta }_{k}}$ with those from Theorem I.1 (see SM C VII). This is accomplished by applying in each case a suitable unitary transformation that brings the stabilizer ${\rm{St}}({\mathfrak{s}})$ to a more convenient form (cf. SM A III).

### Example: ${{\mathfrak{a}}}_{9}(n)$

Consider the example of ${{\mathfrak{a}}}_{9}={\langle XY,XZ\rangle }_{{\rm{Lie}}}$, which produces the subalgebra ${{\mathfrak{a}}}_{9}(n)\subseteq {\mathfrak{su}}({2}^{n})$ generated by:

12 $${X}_{1}{Y}_{2},{X}_{1}{Z}_{2},{X}_{2}{Y}_{3},{X}_{2}{Z}_{3},\ldots ,{X}_{n-1}{Y}_{n},{X}_{n-1}{Z}_{n}.$$

Let us sketch the above steps in the strategy of the proof of Theorem I.1 in the case ${\mathfrak{s}}={{\mathfrak{a}}}_{9}(n)$.

1. The stabilizer ${\rm{St}}({\mathfrak{s}})$ is the set of all Pauli strings $P\in {{\mathcal{P}}}_{n}$ such that [*a*, *P*] = 0 for every $a\in {\mathfrak{s}}$. It is enough to check this for all *a* in the list of generators (12), which means that the substring of *P* in positions *j*, *j* + 1 commutes with *XY* and *XZ* for all 1 ≤ *j* ≤ *n* − 1. By inspection, we find St(*XY*, *XZ*) = {*II*, *XI*, *YX*, *ZX*}, so these are the only possible such substrings of *P*. This gives ${\rm{St}}({\mathfrak{s}})=\{{I}^{\otimes n},{X}_{1},{Y}_{1}{X}_{2},{Z}_{1}{X}_{2}\}$.
2. The centralizer ${\mathfrak{su}}{({2}^{n})}^{{\rm{St}}({\mathfrak{s}})}$ is the set of all $a\in {\mathfrak{su}}({2}^{n})$ such that [*a*, *P*] = 0 for every $P\in {\rm{St}}({\mathfrak{s}})$; hence it contains ${\mathfrak{s}}$. As the center of ${\rm{St}}({\mathfrak{s}})$ is trivial, we have ${{\mathfrak{g}}}_{9}(n)={\mathfrak{su}}{({2}^{n})}^{{\rm{St}}({\mathfrak{s}})}$. To illustrate this last step, we mention that ${\rm{St}}({{\mathfrak{a}}}_{15}(n))=\{{I}^{\otimes n},{X}_{1}\}$. In this case, ${X}_{1}\in {\mathfrak{su}}{({2}^{n})}^{{X}_{1}}$ is central and we have to quotient by it to obtain ${{\mathfrak{g}}}_{15}(n)={\mathfrak{su}}{({2}^{n})}^{{X}_{1}}/{\rm{span}}\{{X}_{1}\}$.
3. We saw above that ${\mathfrak{s}}\subseteq {{\mathfrak{g}}}_{9}(n)$. Now we find an involution *θ*_9_ of ${{\mathfrak{g}}}_{9}(n)$ such that ${\mathfrak{s}}\subseteq {{\mathfrak{g}}}_{9}{(n)}^{{\theta }_{9}}$, the set of fixed points under *θ*_9_. Since *θ*_9_ respects the Lie brackets, it is enough to check *θ*_9_(*a*) = *a* only for the generators (12). Explicitly, we let *θ*_9_(*a*) = −*Q*_9_*a*^*T*^*Q*_9_ where *Q*_9_ = *IYZZ* ⋯ *Z*.
4. We prove by induction on *n* that ${{\mathfrak{a}}}_{9}(n)={{\mathfrak{g}}}_{9}{(n)}^{{\theta }_{9}}$. To show that any $a\in i{{\mathcal{P}}}_{n}\cap {{\mathfrak{g}}}_{9}{(n)}^{{\theta }_{9}}$ with *n* ≥ 4 is in ${{\mathfrak{a}}}_{9}(n)$, we first take suitable commutators of *a* with the generators (12) to produce $b\in i{{\mathcal{P}}}_{n}\cap {{\mathfrak{g}}}_{k}{(n)}^{{\theta }_{k}}$ that has *I* in some position *j* ≥ 3. Erasing the *I* gives an element $c\in {{\mathfrak{g}}}_{9}{(n-1)}^{{\theta }_{9}}$, which by induction is in ${{\mathfrak{a}}}_{9}(n-1)$. Inserting *I* back in *j*-th place in *c* gives that $b\in {{\mathfrak{a}}}_{9}(n)$.
5. As ${\rm{St}}({\mathfrak{s}})\cong \{{I}^{\otimes n},{X}_{1},{Y}_{1},{Z}_{1}\}$, we can simplify ${{\mathfrak{g}}}_{9}{(n)}^{{\theta }_{9}}$ by applying a unitary transformation *a* ↦ *UaU*^†^ that takes ${\rm{St}}({\mathfrak{s}})$ to {*I*^⊗*n*^, *X*_1_, *Y*_1_, *Z*_1_}. Explicitly, we take $U={e}^{i\frac{\pi }{4}{X}_{1}{X}_{2}}$. Then ${{\mathfrak{g}}}_{9}(n)\cong {\mathfrak{su}}{({2}^{n})}^{\{{X}_{1},{Y}_{1},{Z}_{1}\}}=I\otimes {\mathfrak{su}}({2}^{n-1})\cong {\mathfrak{su}}({2}^{n-1})$. The involution *θ*_9_(*a*) = −*Q*_9_*a*^*T*^*Q*_9_ gets transformed to $-{\tilde{Q}}_{9}{a}^{T}{\tilde{Q}}_{9}$, where ${\tilde{Q}}_{9}=U{Q}_{9}{U}^{T}$ in this case happens to be = *Q*_9_. Restricted to ${\mathfrak{su}}({2}^{n-1})$, this gives the involution −*Qa*^*T*^*Q* with *Q* = *YZZ* ⋯ *Z*, whose fixed points are $\cong {\mathfrak{sp}}({2}^{n-2})$ because *Q*^*T*^ = −*Q*.

We conclude that ${{\mathfrak{a}}}_{9}(n)\cong {\mathfrak{sp}}({2}^{n-2})$.

## Supplementary information

Supplemental Material

## Data Availability

Data generated and analyzed during the current study can be reproduced from the code which is available at ref. ^109^.
