# Supplementary material for: Classification of dynamical Lie algebras of 2-local spin systems on linear, circular and fully connected topologies
Source: npj Quantum Inf. 2024 Nov 6;10(1):110. doi: 10.1038/s41534-024-00900-2 (PMC11540907; doi:10.1038/s41534-024-00900-2)
Supplement: Supplementary file 1 — Supplemental Material [file 41534_2024_900_MOESM1_ESM.pdf]

Supplemental Material

A. PRELIMINARIES ON PAULI STRINGS AND $\mathfrak{su}(2^n)$

Length-n Pauli strings, when multiplied with the imaginary unit i, form a natural basis for the Lie algebra $\mathfrak{u}(2^n)$ of skew-Hermitian matrices. Because of this and their other remarkable properties, Pauli strings have been excessively used in this and many other works. In this section, we review the notation and basic properties of Pauli strings, the Lie algebra $\mathfrak{su}(2^n)$, and its subalgebras $\mathfrak{so}(2^n)$ and $\mathfrak{sp}(2^{n-1})$. We also discuss involutions of Lie algebras, and particularly of $\mathfrak{su}(2^n)$.

I. Pauli strings

Throughout the paper, we work with the *Pauli matrices*

$$\sigma_0 = I = \begin{pmatrix} 1 & 0 \\ 0 & 1 \end{pmatrix}, \qquad \sigma_1 = X = \begin{pmatrix} 0 & 1 \\ 1 & 0 \end{pmatrix}, \qquad \sigma_2 = Y = \begin{pmatrix} 0 & -i \\ i & 0 \end{pmatrix}, \qquad \sigma_3 = Z = \begin{pmatrix} 1 & 0 \\ 0 & -1 \end{pmatrix},$$

including the identity matrix I, which form a basis for the real vector space of 2×2 Hermitian matrices. We will denote by A^T the transpose of a matrix, and by $A^\dagger$ its Hermitian conjugate (which is obtained from A^T by taking complex conjugates of all entries). Thus, $A^\dagger = A$ for all $A \in \mathcal{P}_1 := \{I, X, Y, Z\}$. On the other hand, we have

$$Y^T = -Y, \qquad A^T = A \quad \text{for} \quad A = I, X, Z.$$

Fix a positive number n. Length-n *Pauli strings* are tensor products of n Pauli matrices of the form

$$a = A^1 \otimes A^2 \otimes \cdots \otimes A^n, \qquad A^j \in \mathcal{P}_1 \tag{A1}$$

(where the superscripts are indices not powers). We denote the set of all such Pauli strings by $\mathcal{P}_n := \{I, X, Y, Z\}^{\otimes n}$. Every $a \in \mathcal{P}_n$ is a linear operator on the Hilbert space $(\mathbb{C}^2)^{\otimes n}$ of n qubits, so a can be represented as a matrix of size $2^n \times 2^n$ (by the Kronecker product). In particular, $I^{\otimes n}$ is the $2^n \times 2^n$ identity matrix. The Hermitian conjugate and transpose of a Pauli string are done componentwise:

$$\begin{aligned} a^\dagger &= (A^1)^\dagger \otimes (A^2)^\dagger \otimes \cdots \otimes (A^n)^\dagger = a, \\ a^T &= (A^1)^T \otimes (A^2)^T \otimes \cdots \otimes (A^n)^T = (-1)^{\#\{A^j = Y\}} a. \end{aligned}$$

All Pauli strings are Hermitian, and $\mathcal{P}_n$ is a basis (over $\mathbb{R}$) of the vector space of $2^n \times 2^n$ Hermitian matrices.

To shorten the notation, we will often omit the tensor product signs in Pauli strings, so (A1) will be written as $a = A^1 A^2 \cdots A^n$. For example, we will write

$$XX = X \otimes X, \qquad XY = X \otimes Y, \qquad Z \cdots Z = Z^{\otimes n}, \quad \text{etc.}$$

For $A \in \mathcal{P}_1$ and $1 \le j \le n$, we will denote by

$$A_j := I^{\otimes (j-1)} \otimes A \otimes I^{\otimes (n-j)} \tag{A2}$$

the linear operator A acting on the j-th qubit. For example, for $n = 3$,

$$X_1 = XII = X \otimes I \otimes I, \qquad Z_2 = IZI = I \otimes Z \otimes I, \qquad X_1 Z_2 Y_3 = XZY = X \otimes Z \otimes Y, \quad \text{etc.}$$

With this notation, we distinguish

$$A_1 A_2 \cdots A_n = AA \cdots A = A \otimes A \otimes \cdots \otimes A = A^{\otimes n}$$

from (A1), where in the latter the tensor factors $A^1, \ldots, A^n$ are allowed to be different.

When there is a danger to confuse the tensor product and the matrix product, we will use $\cdot$ for the product of matrices. We have:

$$X \cdot Y = iZ = -Y \cdot X, \qquad Y \cdot Z = iX = -Z \cdot Y, \qquad Z \cdot X = iY = -X \cdot Z,$$

and each Pauli matrix squares to the identity:

$$X \cdot X = Y \cdot Y = Z \cdot Z = I.$$

The matrix product of Pauli strings is done componentwise:

$$(A^1 \otimes \cdots \otimes A^n) \cdot (B^1 \otimes \cdots \otimes B^n) = (A^1 \cdot B^1) \otimes \cdots \otimes (A^n \cdot B^n).$$

From here, it is easy to deduce the following important properties of Pauli strings.

Lemma A.1. *For any $a, b \in \mathcal{P}_n$, we have $a \cdot a = I^{\otimes n}$ and $a \cdot b = \pm b \cdot a$. Hence, any two Pauli strings either commute or anticommute.*

Notice that the product of two Pauli strings is again a Pauli string, up to a multiple of $\pm 1, \pm i$. Thus, the set $\{\pm a, \pm ia \,|\, a \in \mathcal{P}_n\}$ is a group under the matrix product, called the *Pauli group*. The following corollary of Lemma A.1 will be useful in the future.

Corollary A.1. *For any $a, b \in \mathcal{P}_n$, if $[a, b] := a \cdot b - b \cdot a \neq 0$, then $[a, [a, b]] = 4b$.*

Proof. When $[a, b] \neq 0$, we have $[a, b] = 2a \cdot b$ and $[a, [a, b]] = 4a \cdot a \cdot b = 4b$. □

Another important consequence of Lemma A.1 is *Euler's formula*

$$e^{i\theta a} = (\cos\theta) I^{\otimes n} + i(\sin\theta) a, \qquad a \in \mathcal{P}_n, \;\; \theta \in \mathbb{R}. \tag{A3}$$

A useful special case is $\theta = \pi/2$; then

$$e^{i\frac{\pi}{2} a} = ia, \qquad a \in \mathcal{P}_n. \tag{A4}$$

Note that any $a \in \mathcal{P}_n$ is Hermitian (i.e., $a^\dagger = a$), ia is skew-Hermitian (i.e., $(ia)^\dagger = -ia$), and $U = e^{i\theta a}$ is unitary (i.e., $UU^\dagger = I^{\otimes n}$). In the following, we will use the following corollary of Euler's formulas (A3), (A4).

Corollary A.2. *For any anticommuting $a, b \in \mathcal{P}_n$ and a real number θ, we have*

$$e^{i\theta a} \cdot b = b \cdot e^{-i\theta a}. \tag{A5}$$

In particular,

$$e^{i\frac{\pi}{4} a}\, b\, e^{-i\frac{\pi}{4} a} = ia \cdot b. \tag{A6}$$

II. The Lie algebras $\mathfrak{su}(N)$, $\mathfrak{so}(N)$, and $\mathfrak{sp}(N)$

The purpose of this subsection is to review some standard terminology concerning Lie algebras, especially $\mathfrak{su}(N)$, $\mathfrak{so}(N)$, and $\mathfrak{sp}(N)$. We fix a positive integer N; later we will have $N = 2^n$ where n is the number of qubits.

The set $\mathbb{C}^{N\times N}$ of $N \times N$ matrices with complex entries is a vector space over $\mathbb{C}$, as matrices can be added or multiplied by complex scalars. Then $\mathbb{C}^{N\times N}$ is an *associative algebra* under the matrix product ab, i.e., ab is bilinear (depends linearly on both a or b) and associative: $a(bc) = (ab)c$. Under the *commutator bracket* $[a, b] = ab - ba$, we get a complex Lie algebra denoted as $\mathfrak{gl}(N, \mathbb{C})$.

In general, a *Lie algebra* is defined as a vector space $\mathfrak{g}$ (over a field such as $\mathbb{C}$ or $\mathbb{R}$), equipped with a bilinear operation $[a, b] \in \mathfrak{g}$ for $a, b \in \mathfrak{g}$, which satisfies the following skew-symmetry and Jacobi identity:

$$[a, b] = -[b, a], \qquad [a, [b, c]] = [[a, b], c] + [b, [a, c]].$$

It is convenient to use the notation $\mathrm{ad}_a(b) := [a, b]$ for $a, b \in \mathfrak{g}$. Then ad_a is a linear operator on $\mathfrak{g}$ for every $a \in \mathfrak{g}$. A trivial example of a Lie algebra is any vector space $\mathfrak{g}$ with the zero bracket $[a, b] = 0$ for all $a, b \in \mathfrak{g}$; such Lie algebras are called *Abelian.*

A *subalgebra* $\mathfrak{s}$ of a Lie algebra $\mathfrak{g}$ is a subspace (i.e., closed under vector addition and scalar multiplication), which is also closed under the bracket: $a, b \in \mathfrak{s} \Rightarrow [a, b] \in \mathfrak{s}$. For example, the set $\mathfrak{sl}(N, \mathbb{C})$ of all $N \times N$ complex matrices with trace 0 is a subalgebra of $\mathfrak{gl}(N, \mathbb{C})$; hence, it is itself a Lie algebra. Recall that an *ideal* in a Lie algebra $\mathfrak{g}$ is a subspace $\mathfrak{s}$ such that $a \in \mathfrak{g}$, $b \in \mathfrak{s} \Rightarrow [a, b] \in \mathfrak{s}$. For example, both $\mathfrak{sl}(N, \mathbb{C})$ and $\mathbb{C}I_N$ (where I_N is the $N \times N$ identity matrix) are ideals of $\mathfrak{gl}(N, \mathbb{C})$, and

$$\mathfrak{gl}(N, \mathbb{C}) = \mathfrak{sl}(N, \mathbb{C}) \oplus \mathbb{C}I_N$$

is a direct sum of not just subspaces but of commuting subalgebras and ideals. When we write a direct sum of Lie algebras, we will always mean that the summands are subalgebras that commute with each other. The space $\mathbb{C}I_N$ is the *center* $Z(\mathfrak{g})$ of $\mathfrak{g} = \mathfrak{gl}(N, \mathbb{C})$, i.e., the set of all $c \in \mathfrak{g}$ such that $[c, a] = 0$ for all $a \in \mathfrak{g}$.

The Lie algebra $\mathfrak{sl}(N, \mathbb{C})$ is *simple*, which means that it is not Abelian and has no ideals other than the trivial $\{0\}$ and the whole algebra. Other examples of simple Lie algebras over $\mathbb{C}$ are provided by the *orthogonal* Lie algebras $\mathfrak{so}(N, \mathbb{C})$ and the *symplectic* Lie algebras $\mathfrak{sp}(2N, \mathbb{C})$. Let us recall that $\mathfrak{so}(N, \mathbb{C})$ is defined as the set of all complex skew-symmetric matrices (i.e., such that $a^T = -a$), and it is a subalgebra of $\mathfrak{sl}(N, \mathbb{C})$. Consider the $2N \times 2N$ matrix

$$J_{2N} := \begin{pmatrix} 0 & I_N \\ -I_N & 0 \end{pmatrix}.$$

Then $\mathfrak{sp}(2N, \mathbb{C})$ is defined as the set of all $a \in \mathfrak{gl}(2N, \mathbb{C})$ such that $a^T J_{2N} = -J_{2N} a$; this is a subalgebra of $\mathfrak{sl}(2N, \mathbb{C})$.

In this paper, we will work with Lie algebras over $\mathbb{R}$. The set of all skew-Hermitian matrices (i.e., satisfying $a^\dagger = -a$) is a real vector space, and is closed under the commutator; hence, it is a real Lie algebra, denoted $\mathfrak{u}(N)$. Imposing that the trace of the matrix is 0, we get the Lie algebra $\mathfrak{su}(N)$. If a matrix has real entries, then it is skew-Hermitian if and only if it is skew-symmetric. Thus, we have the subalgebra

$$\mathfrak{so}(N) := \mathfrak{so}(N, \mathbb{R}) = \{a \in \mathfrak{su}(N) \,|\, a^T = -a\} \subset \mathfrak{su}(N). \tag{A7}$$

On the other hand, the Lie algebra of real symplectic matrices $\mathfrak{sp}(2N, \mathbb{R})$ is *not* a subalgebra of $\mathfrak{su}(2N)$. Instead of it, the relevant subalgebra is

$$\mathfrak{sp}(N) := \mathfrak{sp}(2N, \mathbb{C}) \cap \mathfrak{su}(2N) = \{a \in \mathfrak{su}(2N) \,|\, a^T J_{2N} = -J_{2N} a\} \subset \mathfrak{su}(2N). \tag{A8}$$

The Lie algebras $\mathfrak{su}(N)$, $\mathfrak{so}(N)$, $\mathfrak{sp}(N)$ are simple and *compact* (they are Lie algebras of compact Lie groups). Their dimensions over $\mathbb{R}$ are given by:

$$\dim \mathfrak{su}(N) = N^2 - 1, \qquad \dim \mathfrak{so}(N) = \frac{1}{2} N(N-1), \qquad \dim \mathfrak{sp}(N) = N(2N+1). \tag{A9}$$

It is known (see e.g. [1, 2]) that any subalgebra of $\mathfrak{u}(N)$ is either Abelian or a direct sum of a center (which could be $\{0\}$) and Lie algebras isomorphic to one of $\mathfrak{su}$, $\mathfrak{so}$, $\mathfrak{sp}$ or to one of five exceptional compact simple Lie algebras (cf. Proposition I.1). For completeness, we provide the proof of this important fact here.

Proposition A.1. *Any subalgebra of $\mathfrak{u}(N)$ is either Abelian or a direct sum of compact simple Lie algebras and a center.*

Proof. First, recall that the trace form $(a, b) = \mathrm{Tr}[ab]$ is negative definite on $\mathfrak{u}(N)$. Indeed, one can see that

$$\mathrm{Tr}[H^2] = \sum_{j=1}^{N} \lambda_j^2 > 0$$

for any nonzero Hermitian matrix $H \in i\mathfrak{u}(N)$ with eigenvalues $\lambda_1, \ldots, \lambda_N$, because all λ_j are real. Second, the trace form $(\cdot, \cdot)$ is bilinear, symmetric and invariant; the latter meaning that

$$([a, b], c) = -(b, [a, c]), \qquad a, b, c \in \mathfrak{u}(N).$$

All of these follow easily from the properties of the trace.

Now let $\mathfrak{g}$ be a subalgebra of $\mathfrak{u}(N)$. Then the same proof as in [3], Corollary 4.25, shows that $\mathfrak{g}$ is *reductive*, i.e., it is a direct sum of simple or Abelian ideals. Indeed, for any ideal $\mathfrak{s}$ of $\mathfrak{g}$, we have an orthogonal direct sum

$$\mathfrak{g} = \mathfrak{s} \oplus \mathfrak{s}^\perp,$$

due to the definiteness of the trace form. Furthermore, the invariance of the trace form implies that $\mathfrak{s}^\perp$ is itself an ideal of $\mathfrak{g}$. As both $\mathfrak{s}$ and $\mathfrak{s}^\perp$ are ideals, they must commute: $[\mathfrak{s}, \mathfrak{s}^\perp] \subseteq \mathfrak{s} \cap \mathfrak{s}^\perp = \{0\}$. Thus, $\mathfrak{g}$ is a direct sum of commuting ideals. Suppose that $\dim \mathfrak{s} > 1$ and $\mathfrak{s}$ is not simple as a Lie algebra. Then $\mathfrak{s}$ has a nonzero proper ideal $\mathfrak{t}$. From $[\mathfrak{t}, \mathfrak{s}] \subseteq \mathfrak{t}$ and $[\mathfrak{t}, \mathfrak{s}^\perp] = \{0\}$, we get that $[\mathfrak{t}, \mathfrak{g}] \subseteq \mathfrak{t}$, so $\mathfrak{t}$ is an ideal of $\mathfrak{g}$. Hence, we can proceed by induction on $\dim \mathfrak{g}$ and break $\mathfrak{g}$ into a direct sum of commuting ideals, each of which is either simple or 1-dimensional (Abelian).

Finally, we note that any simple subalgebra $\mathfrak{g}$ of $\mathfrak{u}(N)$ is compact, i.e., the Lie group $e^{\mathfrak{g}}$ is compact as a closed subgroup of the compact Lie group $\mathrm{U}(N)$ (see e.g. [3], Proposition 4.27, whose proof still applies). The classification of all compact simple Lie algebras is due to Cartan, and can be found in standard textbooks; for example in [3], Chapter VI. □

Let now $N = 2^n$ where n is the number of qubits. Then a basis for $\mathfrak{u}(2^n)$ is given by $i\mathcal{P}_n$ (see Sect. A I). To get a basis for $\mathfrak{su}(2^n)$, we just have to remove $iI^{\otimes n}$, since $I^{\otimes n} = I_N$ is the identity matrix. A basis for $\mathfrak{so}(2^n)$ consists of all ia where $a \in \mathcal{P}_n$ is a Pauli string containing an odd number of Y's. Finally, to describe the subalgebra $\mathfrak{sp}(2^{n-1}) \subset \mathfrak{su}(2^n)$, we observe that

$$Y_1 = Y \otimes I^{\otimes(n-1)} = -i \begin{pmatrix} 0 & I_{\frac{N}{2}} \\ -I_{\frac{N}{2}} & 0 \end{pmatrix} = -iJ_N.$$

Therefore,

$$\mathfrak{sp}(2^{n-1}) = \big\{ a \in \mathfrak{su}(2^n) \,\big|\, a^T \cdot Y_1 = -Y_1 \cdot a \big\}. \tag{A10}$$

The following definition plays a crucial role throughout the paper.

Definition A.1. *For a Lie algebra $\mathfrak{g}$ and a subset $\mathcal{A} \subset \mathfrak{g}$, we define $\langle \mathcal{A} \rangle_{\mathrm{Lie}}$ to be the minimal (under inclusion) subalgebra of $\mathfrak{g}$ that contains $\mathcal{A}$. We say that $\langle \mathcal{A} \rangle_{\mathrm{Lie}}$ is the subalgebra* generated *by $\mathcal{A}$, and that $\mathcal{A}$ is a set of* generators *of $\langle \mathcal{A} \rangle_{\mathrm{Lie}}$. In the case where $\mathcal{A} \subset \mathcal{P}_n$ is a set of Pauli strings, we will slightly abuse the notation and write $\langle \mathcal{A} \rangle_{\mathrm{Lie}}$ for the subalgebra of $\mathfrak{su}(2^n)$ generated by the subset $i\mathcal{A} \subset \mathfrak{su}(2^n)$.*

More explicitly, it follows from the Jacobi identity that $\langle \mathcal{A} \rangle_{\mathrm{Lie}}$ is the set of all linear combinations of all nested commutators of the form

$$\mathrm{ad}_{a_1} \mathrm{ad}_{a_2} \cdots \mathrm{ad}_{a_r}(a_{r+1}) = [a_1, [a_2, [\cdots [a_r, a_{r+1}] \cdots]]], \qquad a_j \in \mathcal{A}, \;\; r \geq 0, \tag{A11}$$

where $r = 0$ corresponds to an empty commutator $= a_1$. The following simple observation will be useful.

Lemma A.2. *For any subset $\mathcal{A} \subset \mathcal{P}_n$, the Lie algebra $\langle \mathcal{A} \rangle_{\mathrm{Lie}} \subseteq \mathfrak{su}(2^n)$ generated by $i\mathcal{A}$ has a basis consisting of Pauli strings times i. In other words,*

$$\langle \mathcal{A} \rangle_{\mathrm{Lie}} = \mathrm{span}_{\mathbb{R}}\big(i\mathcal{P}_n \cap \langle \mathcal{A} \rangle_{\mathrm{Lie}} \big).$$

Proof. By definition, $\langle \mathcal{A} \rangle_{\mathrm{Lie}}$ is linearly spanned over $\mathbb{R}$ by all elements of the form (A11) with $a_j \in i\mathcal{A} \subset i\mathcal{P}_n$. All such elements are scalar multiples of Pauli strings, i.e., lie in $i\mathbb{R}\mathcal{P}_n$. From any spanning set, one can choose a subset that forms a basis. □

III. Involutions of $\mathfrak{su}(2^n)$

In this subsection, we explain how we can describe subalgebras of $\mathfrak{su}(2^n)$ as fixed points of involutions. We start more generally by recalling that an *isomorphism* from a Lie algebra $\mathfrak{g}$ to another Lie algebra $\mathfrak{g}_1$ is an invertible linear transformation $\varphi \colon \mathfrak{g} \to \mathfrak{g}_1$ that is compatible with the bracket, i.e., $\varphi([a, b]) = [\varphi(a), \varphi(b)]$ for all $a, b \in \mathfrak{g}$. We write $\mathfrak{g} \cong \mathfrak{g}_1$ to indicate that $\mathfrak{g}$ is isomorphic to $\mathfrak{g}_1$. An *automorphism* of $\mathfrak{g}$ is an isomorphism $\varphi \colon \mathfrak{g} \to \mathfrak{g}$. The set of fixed points of φ is defined as:

$$\mathfrak{g}^{\varphi} = \big\{ a \in \mathfrak{g} \,\big|\, \varphi(a) = a \big\}. \tag{A12}$$

It is easy to check that $\mathfrak{g}^{\varphi}$ is a subalgebra of $\mathfrak{g}$, called the *fixed-point subalgebra*. An *involution* on $\mathfrak{g}$ is an automorphism $\theta \colon \mathfrak{g} \to \mathfrak{g}$ with the property that $\theta(\theta(a)) = a$ for all $a \in \mathfrak{g}$, i.e., $\theta^{-1} = \theta$.

Later, we will need to understand how, for an involution θ, the fixed-point subalgebra $\mathfrak{g}^{\theta}$ transforms under another automorphism φ of $\mathfrak{g}$. The answer is given in the following lemma.

Lemma A.3. *Let φ be an automorphism of a Lie algebra $\mathfrak{g}$, and θ be an involution of $\mathfrak{g}$. Then $\varphi\theta\varphi^{-1}$ is an involution of $\mathfrak{g}$, and we have $\varphi(\mathfrak{g}^{\theta}) = \mathfrak{g}^{\varphi\theta\varphi^{-1}}$.*

Proof. Note that $\varphi\theta\varphi^{-1}$ is an automorphism of $\mathfrak{g}$, since the composition of isomorphisms is again an isomorphism. It is an involution of $\mathfrak{g}$, because $(\varphi\theta\varphi^{-1})^{-1} = \varphi\theta^{-1}\varphi^{-1} = \varphi\theta\varphi^{-1}$. To check the claim about the fixed points, suppose that $a \in \mathfrak{g}^{\theta}$, i.e., $\theta(a) = a$. Then

$$(\varphi\theta\varphi^{-1})\varphi(a) = \varphi(\theta(a)) = \varphi(a) \quad \Rightarrow \quad \varphi(a) \in \mathfrak{g}^{\varphi\theta\varphi^{-1}}.$$

Conversely, if $b \in \mathfrak{g}^{\varphi\theta\varphi^{-1}}$, then the above calculation shows that $a = \varphi^{-1}(b) \in \mathfrak{g}^{\theta}$. □

Now we will discuss how to construct automorphisms and involutions of $\mathfrak{su}(N)$.

Lemma A.4. *Suppose that U and Q are unitary $N \times N$ matrices, and $Q^T = \pm Q$. Then the formulas*

$$\varphi(a) = UaU^\dagger, \qquad \theta(a) = -Qa^TQ^\dagger, \qquad a \in \mathfrak{su}(N),$$

define an automorphism φ and an involution θ of $\mathfrak{su}(N)$. Moreover, we have

$$(\varphi\theta\varphi^{-1})(a) = -(UQU^T)a^T(UQU^T)^\dagger, \qquad a \in \mathfrak{su}(N).$$

Proof. First, note that φ is invertible with $\varphi^{-1}(a) = U^\dagger aU$. It is clear that $\varphi([a,b]) = [\varphi(a), \varphi(b)]$, because

$$\varphi(a)\varphi(b) = UaU^\dagger UbU^\dagger = UabU^\dagger = \varphi(ab)$$

for any two matrices a, b. (This means that φ is an automorphism of the associative algebra $\mathbb{C}^{N\times N}$.)

To check that θ is an involution, we calculate

$$\theta(\theta(a)) = \theta(-Qa^TQ^\dagger) = Q(Q^\dagger)^T(a^T)^TQ^TQ^\dagger = Q(\pm Q^\dagger)a(\pm Q)Q^\dagger = a,$$

where we used that $(Q^\dagger)^T = (Q^T)^\dagger$. Next, we have

$$\theta(ab) = -Q(ab)^TQ^\dagger = -Qb^Ta^TQ^\dagger = -Qb^TQ^\dagger Qa^TQ^\dagger = -\theta(b)\theta(a).$$

This implies that $\theta([a,b]) = [\theta(a), \theta(b)]$ and proves that θ is an involution of $\mathfrak{su}(N)$.

Finally, we find

$$\begin{aligned}(\varphi\theta\varphi^{-1})(a) &= \varphi\big(\theta(U^\dagger aU)\big)\\ &= -\varphi\big(QU^Ta^T(U^\dagger)^TQ^\dagger\big)\\ &= -UQU^Ta^T(U^\dagger)^TQ^\dagger U^\dagger\\ &= -(UQU^T)a^T(UQU^T)^\dagger,\end{aligned}$$

as claimed. □

Example A.1. The subalgebra $\mathfrak{so}(N) \subset \mathfrak{su}(N)$ is the fixed-point subalgebra of the involution $a \mapsto -a^T$ (see (A7)). Similarly, we see from (A8) that $\mathfrak{sp}(N) = \mathfrak{su}(2N)^\theta$, where $\theta(a) = -Qa^TQ^\dagger$ as in Lemma A.4, with $Q = -iJ_{2N}$.

It is well known, in general, that for an involution $\theta(a) = -Qa^TQ^\dagger$ as in Lemma A.4, the fixed-point subalgebra $\mathfrak{su}(N)^\theta \cong \mathfrak{so}(N)$ when $Q^T = Q$, and $\mathfrak{su}(N)^\theta \cong \mathfrak{sp}(N/2)$ when $Q^T = -Q$ (in which case N must be even). For completeness, we will present the proof of these facts in the special case of interest to us: when $N = 2^n$ and $Q \in \mathcal{P}_n$ is a length-n Pauli string. Note that all Pauli strings Q satisfy $Q = Q^\dagger = Q^{-1}$ and $Q^T = \pm Q$.

Lemma A.5. *For any Pauli string $Q \in \mathcal{P}_n$, there exists a unitary $2^n \times 2^n$ matrix U such that*

$$UQU^T = \begin{cases} I^{\otimes n}, & \text{if } Q^T = Q,\\ Y_1, & \text{if } Q^T = -Q.\end{cases}$$

Proof. In the case $Q^T = Q$, we let $U = e^{i\frac{\pi}{4}Q}$. Then

$$UQU^T = UQU = U^2Q = e^{i\frac{\pi}{2}Q}Q = iQ\cdot Q = iI^{\otimes n},$$

where we used Euler's formula (A4). The superfluous phase i can be eliminated by applying the unitary transformation $V = e^{-i\frac{\pi}{4}I^{\otimes n}}$, which satisfies $V^T = V$ and $V^2 = -iI^{\otimes n}$.

Suppose now that $Q^T = -Q$, which means that Q contains an odd number of Y's. If Q has a Y in j-th position, let $P = Y_j \cdot Q$ and $U = e^{i\frac{\pi}{4}P}$. Note that

$$P = Y_j \cdot Q = Q \cdot Y_j \quad\Rightarrow\quad P\cdot Q = Q\cdot P = Y_j$$

and

$$P^T = Q^T \cdot Y_j^T = (-Q)\cdot(-Y_j) = Q\cdot Y_j = P.$$

Hence, as above, we find:

$$UQU^T = UQU = U^2Q = e^{i\frac{\pi}{2}P}Q = iP \cdot Q = iY_j.$$

If $j = 1$, we are done (after eliminating the phase i). If $j \neq 1$, we apply the unitary transformation $e^{-i\frac{\pi}{4}Y_1Y_j}$ and obtain:

$$e^{-i\frac{\pi}{4}Y_1Y_j}(iY_j)(e^{-i\frac{\pi}{4}Y_1Y_j})^T = e^{-i\frac{\pi}{2}Y_1Y_j}(iY_j) = -iY_1Y_j \cdot (iY_j) = Y_1,$$

completing the proof. □

Corollary A.3. *Any Pauli string $Q \in \mathcal{P}_n$ defines an involution θ of $\mathfrak{su}(2^n)$ given by $\theta(a) = -Qa^TQ$. The fixed-point subalgebra of this involution is:*

$$\mathfrak{su}(2^n)^\theta \cong \begin{cases} \mathfrak{so}(2^n), & \text{if } Q^T = Q, \\ \mathfrak{sp}(2^{n-1}), & \text{if } Q^T = -Q. \end{cases}$$

B. STATEMENT OF RESULTS

In this section, we present detailed information about all subalgebras of $\mathfrak{su}(4)$ generated by Pauli strings, including a complete list of such subalgebras with their bases, generators, and equivalence under symmetry. We match this list with examples of known spin systems. Next, we discuss how subalgebras of $\mathfrak{su}(4)$ are extended to subalgebras of $\mathfrak{su}(2^n)$ (cf. *Growing the dynamical Lie algebras* in the Results section of the main text). We present the list of all subalgebras of $\mathfrak{su}(8)$ obtained in this way, and the list of subalgebras of $\mathfrak{su}(2^n)$ in the case of open boundary conditions.

I. Classification up to symmetry of subalgebras of $\mathfrak{su}(4)$ generated by Pauli strings

Recall that $\mathfrak{su}(4)$ has a basis over $\mathbb{R}$ consisting of all possible tensor products iAB, where $A, B \in \{I, X, Y, Z\}$, $AB \neq II$. There are $2^{15} - 1 = 32767$ non-empty subsets $\mathcal{A}$ of this basis. For each of them, we run Algorithm 1 in the simplified form from Remark II.1 to determine the Lie algebra $\langle \mathcal{A} \rangle_{\text{Lie}}$ generated by $\mathcal{A}$. We have written code to do this, which is publicly available at [4]. As a result, we have found 202 subalgebras of $\mathfrak{su}(4)$ generated by sets of Pauli strings. Their complete list, with a basis for each subalgebra, is presented in the next subsection, Sect. B II.

As discussed in *Symmetries of the power sets* in the Methods section of the main text, the symmetry group $S_3 \times \mathbb{Z}_2$ acts on $\mathfrak{su}(4)$ as follows: the symmetric group S_3 permutes simultaneously all $\{X, Y, Z\}$, while the non-identity element of $\mathbb{Z}_2$ acts as the flip $AB \rightleftharpoons BA$. By inspection, we have examined the orbits of the action of $S_3 \times \mathbb{Z}_2$ on the 202 subalgebras and found that there are 36 orbits, which are listed in Table S.I below. The full list of all 202 subalgebras in Sect. B II is grouped into orbits, which can easily be verified by hand.

The numbers s, p, e, d in Table S.I are equal to the numbers of: single Paulis (such as XI), single Pauli pairs (such as XI, IX), double equal Paulis (such as XX), and double different Paulis (such as XY), respectively, in the basis of each subalgebra. These are invariant under the action of the symmetry group; hence, subalgebras of $\mathfrak{su}(4)$ with different invariants are not equivalent to each other. It turns out that the only two non-equivalent subalgebras with the same invariants are $\mathfrak{a}_2$ and $\mathfrak{a}_5$.

As in *The power sets* in the Methods section of the main text, we distinguish between three types of subalgebras of $\mathfrak{su}(4)$. The $\mathfrak{a}$-*type* are those that can be generated by a set of products of two Paulis both different from the identity. The $\mathfrak{b}$-*type* are not of $\mathfrak{a}$-type and have a generating set of Pauli strings that contain the identity but come in pairs such as XI, IX or YI, IY or ZI, IZ. The remaining $\mathfrak{c}$-*type* subalgebras have generators that contain some Pauli strings of the form AI without the corresponding term IA or vice versa ($A \in \{X, Y, Z\}$).

When extended to subalgebras of $\mathfrak{su}(2^n)$ on a length-n open spin chain, as in *Growing the dynamical Lie algebras* in the Results section of the main text or in Sect. B IV below, the $\mathfrak{c}$-type subalgebras will behave like the $\mathfrak{b}$-type except for a boundary effect at the first or last site in the chain. As we will insist that every single Pauli generator $A \in \{X, Y, Z\}$ operates on all sites of the chain, in the future we will exclude the $\mathfrak{c}$-type subalgebras of $\mathfrak{su}(2^n)$. However, for completeness, we include them in the list of subalgebras of $\mathfrak{su}(4)$ below.

**Label**	**Basis**	**dim**	**Stabilizer**	**Orbit**	$(\boldsymbol{s},\boldsymbol{p},\boldsymbol{e},\boldsymbol{d})$
$\mathfrak{a}_0$	XX	1	4	3	(0,0,1,0)
$\mathfrak{a}_1$	XY	1	2	6	(0,0,0,1)
$\mathfrak{a}_2$	XY, YX	2	4	3	(0,0,0,2)
$\mathfrak{a}_3$	XX, YZ	2	2	6	(0,0,1,1)
$\mathfrak{a}_4$	XX, YY	2	4	3	(0,0,2,0)
$\mathfrak{a}_5$	XY, YZ	2	2	6	(0,0,0,2)
$\mathfrak{a}_6$	XX, YZ, ZY	3	4	3	(0,0,1,2)
$\mathfrak{a}_7$	XX, YY, ZZ	3	12	1	(0,0,3,0)
$\mathfrak{a}_8$	XX, XZ, IY	3	1	12	(1,0,1,1)
$\mathfrak{a}_9$	XY, XZ, IX	3	2	6	(1,0,0,2)
$\mathfrak{a}_{10}$	XY, YZ, ZX	3	6	2	(0,0,0,3)
$\mathfrak{a}_{11}$	XY, YX, YZ, IY	4	1	12	(1,0,0,3)
$\mathfrak{a}_{12}$	XX, XY, YZ, IZ	4	1	12	(1,0,1,2)
$\mathfrak{a}_{13}$	XX, YY, YZ, IX	4	1	12	(1,0,2,1)
$\mathfrak{a}_{14}$	XX, YY, XY, YX, ZI, IZ	6	4	3	(2,1,2,2)
$\mathfrak{a}_{15}$	XX, XY, XZ, IX, IY, IZ	6	2	6	(3,0,1,2)
$\mathfrak{a}_{16}$	XY, YX, YZ, ZY, YI, IY	6	4	3	(2,1,0,4)
$\mathfrak{a}_{17}$	XX, XY, ZX, ZY, YI, IZ	6	2	6	(2,0,1,3)
$\mathfrak{a}_{18}$	XX, YY, XZ, ZY, XI, IY	6	2	6	(2,0,2,2)
$\mathfrak{a}_{19}$	$XX, XY, ZX, ZY, YZ, YI, IZ$	7	2	6	(2,0,1,4)
$\mathfrak{a}_{20}$	$XX, YY, ZZ, YZ, ZY, XI, IX$	7	4	3	(2,1,3,2)
$\mathfrak{a}_{21}$	$XX, YY, XY, YX, ZX, ZY, XI, YI, ZI, IZ$	10	2	6	(4,1,2,4)
$\mathfrak{a}_{22}$	all Paulis except II	15	12	1	(6,3,3,6)
$\mathfrak{b}_0$	XI, IX	2	4	3	(2,1,0,0)
$\mathfrak{b}_1$	XX, XI, IX	3	4	3	(2,1,1,0)
$\mathfrak{b}_2$	XY, XZ, XI, IX	4	2	6	(2,1,0,2)
$\mathfrak{b}_3$	XI, YI, ZI, IX, IY, IZ	6	12	1	(6,3,0,0)
$\mathfrak{b}_4$	$XX, XY, XZ, XI, IX, IY, IZ$	7	2	6	(4,1,1,2)
$\mathfrak{c}_0$	XI	1	2	6	(1,0,0,0)
$\mathfrak{c}_1$	XY, XI	2	1	12	(1,0,0,1)
$\mathfrak{c}_2$	XX, XI	2	2	6	(1,0,1,0)
$\mathfrak{c}_3$	XI, IY	2	2	6	(2,0,0,0)
$\mathfrak{c}_4$	XY, XI, IY	3	2	6	(2,0,0,1)
$\mathfrak{c}_5$	XI, YI, ZI	3	6	2	(3,0,0,0)
$\mathfrak{c}_6$	XX, XY, XI, IZ	4	1	12	(2,0,1,1)
$\mathfrak{c}_7$	XI, IX, YI, ZI	4	2	6	(4,1,0,0)

Table S.I. List of all subalgebras of $\mathfrak{su}(4)$ generated by Pauli strings, up to symmetry $S_3 \times \mathbb{Z}_2$. For each subalgebra, we have listed: a basis (over $\mathbb{R}$, after multiplication by i), its dimension, the order of the stabilizer, the order of the orbit under the action of $S_3 \times \mathbb{Z}_2$, and the invariants s, p, e, d. Note that the orders of all orbits add up to 202.

For each of our subalgebras of $\mathfrak{su}(4)$, we list below a minimal set of generators and the isomorphism class of the Lie algebra. Commuting direct summands are denoted with $\oplus$. Note also that $\mathfrak{so}(3) \cong \mathfrak{su}(2)$ and $\mathfrak{so}(4) \cong \mathfrak{su}(2) \oplus \mathfrak{su}(2)$.

$$
\begin{aligned}
\mathfrak{a}_0 &= \langle XX \rangle_{\mathrm{Lie}} \cong \mathfrak{u}(1),\\
\mathfrak{a}_1 &= \langle XY \rangle_{\mathrm{Lie}} \cong \mathfrak{u}(1),\\
\mathfrak{a}_2 &= \langle XY, YX \rangle_{\mathrm{Lie}} \cong \mathfrak{u}(1) \oplus \mathfrak{u}(1),\\
\mathfrak{a}_3 &= \langle XX, YZ \rangle_{\mathrm{Lie}} \cong \mathfrak{u}(1) \oplus \mathfrak{u}(1),\\
\mathfrak{a}_4 &= \langle XX, YY \rangle_{\mathrm{Lie}} \cong \mathfrak{u}(1) \oplus \mathfrak{u}(1),\\
\mathfrak{a}_5 &= \langle XY, YZ \rangle_{\mathrm{Lie}} \cong \mathfrak{u}(1) \oplus \mathfrak{u}(1),\\
\mathfrak{a}_6 &= \langle XX, YZ, ZY \rangle_{\mathrm{Lie}} \cong \mathfrak{u}(1) \oplus \mathfrak{u}(1) \oplus \mathfrak{u}(1),\\
\mathfrak{a}_7 &= \langle XX, YY, ZZ \rangle_{\mathrm{Lie}} \cong \mathfrak{u}(1) \oplus \mathfrak{u}(1) \oplus \mathfrak{u}(1),\\
\mathfrak{a}_8 &= \langle XX, XZ \rangle_{\mathrm{Lie}} \cong \mathfrak{su}(2),\\
\mathfrak{a}_9 &= \langle XY, XZ \rangle_{\mathrm{Lie}} \cong \mathfrak{su}(2),\\
\mathfrak{a}_{10} &= \langle XY, YZ, ZX \rangle_{\mathrm{Lie}} \cong \mathfrak{u}(1) \oplus \mathfrak{u}(1) \oplus \mathfrak{u}(1),\\
\mathfrak{a}_{11} &= \langle XY, YX, YZ \rangle_{\mathrm{Lie}} \cong \mathfrak{su}(2) \oplus \mathfrak{u}(1),\\
\mathfrak{a}_{12} &= \langle XX, XY, YZ \rangle_{\mathrm{Lie}} \cong \mathfrak{su}(2) \oplus \mathfrak{u}(1),\\
\mathfrak{a}_{13} &= \langle XX, YY, YZ \rangle_{\mathrm{Lie}} \cong \mathfrak{su}(2) \oplus \mathfrak{u}(1),\\
\mathfrak{a}_{14} &= \langle XX, YY, XY \rangle_{\mathrm{Lie}} = \mathfrak{so}(4),\\
\mathfrak{a}_{15} &= \langle XX, XY, XZ \rangle_{\mathrm{Lie}} \cong \mathfrak{su}(2) \oplus \mathfrak{su}(2),\\
\mathfrak{a}_{16} &= \langle XY, YX, YZ, ZY \rangle_{\mathrm{Lie}} = \mathfrak{so}(4),\\
\mathfrak{a}_{17} &= \langle XX, XY, ZX \rangle_{\mathrm{Lie}} \cong \mathfrak{su}(2) \oplus \mathfrak{su}(2),\\
\mathfrak{a}_{18} &= \langle XX, XZ, YY, ZY \rangle_{\mathrm{Lie}} \cong \mathfrak{su}(2) \oplus \mathfrak{su}(2),\\
\mathfrak{a}_{19} &= \langle XX, XY, ZX, YZ \rangle_{\mathrm{Lie}} \cong \mathfrak{su}(2) \oplus \mathfrak{su}(2) \oplus \mathfrak{u}(1),\\
\mathfrak{a}_{20} &= \langle XX, YY, ZZ, ZY \rangle_{\mathrm{Lie}} \cong \mathfrak{su}(2) \oplus \mathfrak{su}(2) \oplus \mathfrak{u}(1),\\
\mathfrak{a}_{21} &= \langle XX, YY, XY, ZX \rangle_{\mathrm{Lie}} \cong \mathfrak{sp}(2),\\
\mathfrak{a}_{22} &= \langle XX, XY, XZ, YX, ZX \rangle_{\mathrm{Lie}} = \mathfrak{su}(4),\\
\mathfrak{b}_0 &= \langle XI, IX \rangle_{\mathrm{Lie}} \cong \mathfrak{u}(1) \oplus \mathfrak{u}(1),\\
\mathfrak{b}_1 &= \langle XX, XI, IX \rangle_{\mathrm{Lie}} \cong \mathfrak{u}(1) \oplus \mathfrak{u}(1) \oplus \mathfrak{u}(1),\\
\mathfrak{b}_2 &= \langle XY, XI, IX \rangle_{\mathrm{Lie}} \cong \mathfrak{su}(2) \oplus \mathfrak{u}(1),\\
\mathfrak{b}_3 &= \langle XI, YI, IX, IY \rangle_{\mathrm{Lie}} \cong \mathfrak{su}(2) \oplus \mathfrak{su}(2),\\
\mathfrak{b}_4 &= \langle XX, XY, XI, IX \rangle_{\mathrm{Lie}} \cong \mathfrak{su}(2) \oplus \mathfrak{su}(2) \oplus \mathfrak{u}(1),\\
\mathfrak{c}_0 &= \langle XI \rangle_{\mathrm{Lie}} \cong \mathfrak{u}(1),\\
\mathfrak{c}_1 &= \langle XY, XI \rangle_{\mathrm{Lie}} \cong \mathfrak{u}(1) \oplus \mathfrak{u}(1),\\
\mathfrak{c}_2 &= \langle XX, XI \rangle_{\mathrm{Lie}} \cong \mathfrak{u}(1) \oplus \mathfrak{u}(1),\\
\mathfrak{c}_3 &= \langle XI, IY \rangle_{\mathrm{Lie}} \cong \mathfrak{u}(1) \oplus \mathfrak{u}(1),\\
\mathfrak{c}_4 &= \langle XY, XI, IY \rangle_{\mathrm{Lie}} \cong \mathfrak{u}(1) \oplus \mathfrak{u}(1) \oplus \mathfrak{u}(1),\\
\mathfrak{c}_5 &= \langle XI, YI, ZI \rangle_{\mathrm{Lie}} \cong \mathfrak{su}(2),\\
\mathfrak{c}_6 &= \langle XX, XY, XI \rangle_{\mathrm{Lie}} \cong \mathfrak{su}(2) \oplus \mathfrak{u}(1),\\
\mathfrak{c}_7 &= \langle XI, YI, IX \rangle_{\mathrm{Lie}} \cong \mathfrak{su}(2) \oplus \mathfrak{u}(1).
\end{aligned}
$$

Finally, we note that

$$
\begin{aligned}
\mathfrak{b}_2 &= \mathfrak{a}_9 \oplus \mathrm{span}\{XI\},\\
\mathfrak{b}_4 &= \mathfrak{a}_{15} \oplus \mathrm{span}\{XI\},
\end{aligned}
$$

are central extensions of $\mathfrak{a}$-type subalgebras.

II. List of all 202 subalgebras of $\mathfrak{su}(4)$ generated by Pauli strings

Here, by inspection, we have grouped the 202 subalgebras of $\mathfrak{su}(4)$ from the previous subsection into orbits of the symmetry group $S_3 \times \mathbb{Z}_2$. For each orbit, we provide its label and its size.

$\mathfrak{a}_0 : 3,\ \{XX\}, \{YY\}, \{ZZ\};$

$\mathfrak{a}_1 : 6,\ \{XY\}, \{XZ\}, \{YX\}, \{YZ\}, \{ZX\}, \{ZY\};$

$\mathfrak{a}_2 : 3,\ \{XY, YX\}, \{XZ, ZX\}, \{YZ, ZY\};$

$\mathfrak{a}_3 : 6,\ \{XX, YZ\}, \{XX, ZY\}, \{YY, XZ\}, \{YY, ZX\}, \{ZZ, XY\}, \{ZZ, YX\};$

$\mathfrak{a}_4 : 3,\ \{XX, YY\}, \{XX, ZZ\}, \{YY, ZZ\};$

$\mathfrak{a}_5 : 6,\ \{XY, YZ\}, \{XZ, ZY\}, \{YX, XZ\}, \{YZ, ZX\}, \{ZX, XY\}, \{ZY, YX\};$

$\mathfrak{a}_6 : 3,\ \{XY, YX, ZZ\}, \{XZ, ZX, YY\}, \{YZ, ZY, XX\};$

$\mathfrak{a}_7 : 1,\ \{XX, YY, ZZ\};$

$\mathfrak{a}_8 : 12,\ \{XX, XY, IZ\}, \{XX, XZ, IY\}, \{XX, YX, ZI\}, \{XX, ZX, YI\},$
$\{YY, YX, IZ\}, \{YY, YZ, IX\}, \{YY, XY, ZI\}, \{YY, ZY, XI\},$
$\{ZZ, ZX, IY\}, \{ZZ, ZY, IX\}, \{ZZ, XZ, YI\}, \{ZZ, YZ, XI\};$

$\mathfrak{a}_9 : 6,\ \{XY, XZ, IX\}, \{YX, YZ, IY\}, \{ZX, ZY, IZ\},$
$\{YX, ZX, XI\}, \{XY, ZY, YI\}, \{XZ, YZ, ZI\};$

$\mathfrak{a}_{10} : 2,\ \{XY, YZ, ZX\}, \{XZ, ZY, YX\};$

$\mathfrak{a}_{11} : 12,\ \{XY, YX, XZ, IX\}, \{XZ, ZX, XY, IX\}, \{XY, YX, ZX, XI\}, \{XZ, ZX, YX, XI\},$
$\{YX, XY, YZ, IY\}, \{YZ, ZY, YX, IY\}, \{YX, XY, ZY, YI\}, \{YZ, ZY, XY, YI\},$
$\{ZX, XZ, ZY, IZ\}, \{ZY, YZ, ZX, IZ\}, \{ZX, XZ, YZ, ZI\}, \{ZY, YZ, XZ, ZI\};$

$\mathfrak{a}_{12} : 12,\ \{XX, XY, YZ, IZ\}, \{XX, XZ, ZY, IY\}, \{XX, YX, ZY, ZI\}, \{XX, ZX, YZ, YI\},$
$\{YY, YX, XZ, IZ\}, \{YY, YZ, ZX, IX\}, \{YY, XY, ZX, ZI\}, \{YY, ZY, XZ, XI\},$
$\{ZZ, ZX, XY, IY\}, \{ZZ, ZY, YX, IX\}, \{ZZ, XZ, YX, YI\}, \{ZZ, YZ, XY, XI\};$

$\mathfrak{a}_{13} : 12,\ \{XX, YY, YZ, IX\}, \{XX, YY, XZ, IY\}, \{XX, YY, ZY, XI\}, \{XX, YY, ZX, YI\},$
$\{XX, ZZ, ZY, IX\}, \{XX, ZZ, XY, IZ\}, \{XX, ZZ, YZ, XI\}, \{XX, ZZ, YX, ZI\},$
$\{YY, ZZ, ZX, IY\}, \{YY, ZZ, YX, IZ\}, \{YY, ZZ, XZ, YI\}, \{YY, ZZ, XY, ZI\};$

$\mathfrak{a}_{14} : 3,\ \{XX, YY, XY, YX, ZI, IZ\}, \{XX, ZZ, XZ, ZX, YI, IY\}, \{YY, ZZ, YZ, ZY, XI, IX\};$

$\mathfrak{a}_{15} : 6,\ \{XX, XY, XZ, IX, IY, IZ\}, \{YY, YX, YZ, IX, IY, IZ\}, \{ZZ, ZX, ZY, IX, IY, IZ\},$
$\{XX, YX, ZX, XI, YI, ZI\}, \{YY, XY, ZY, XI, YI, ZI\}, \{ZZ, XZ, YZ, XI, YI, ZI\};$

$\mathfrak{a}_{16} : 3,\ \{XY, YX, XZ, ZX, XI, IX\}, \{YX, XY, YZ, ZY, YI, IY\}, \{ZX, XZ, ZY, YZ, ZI, IZ\};$

$\mathfrak{a}_{17} : 6,\ \{XX, XY, ZX, ZY, YI, IZ\}, \{YY, YX, ZY, ZX, XI, IZ\}, \{ZZ, ZX, YZ, YX, XI, IY\},$
$\{XX, YX, XZ, YZ, IY, ZI\}, \{YY, XY, YZ, XZ, IX, ZI\}, \{ZZ, XZ, ZY, XY, IX, YI\};$

$\mathfrak{a}_{18} : 6,\ \{XX, YY, XZ, ZY, XI, IY\}, \{XX, ZZ, XY, YZ, XI, IZ\}, \{YY, ZZ, YX, XZ, YI, IZ\},$
$\{XX, YY, ZX, YZ, IX, YI\}, \{XX, ZZ, YX, ZY, IX, ZI\}, \{YY, ZZ, XY, ZX, IY, ZI\};$

$\mathfrak{a}_{19} : 6,\ \{XX, XY, ZX, ZY, YZ, YI, IZ\}, \{XX, YX, XZ, YZ, ZY, IY, ZI\},$
$\{YY, YX, ZY, ZX, XZ, XI, IZ\}, \{YY, XY, YZ, XZ, ZX, IX, ZI\},$
$\{ZZ, ZX, YZ, YX, XY, XI, IY\}, \{ZZ, XZ, ZY, XY, YX, IX, YI\};$

$\mathfrak{a}_{20} : 3,\ \{XX, YY, ZZ, XY, YX, ZI, IZ\}, \{XX, YY, ZZ, XZ, ZX, YI, IY\},$
$\{XX, YY, ZZ, YZ, ZY, XI, IX\};$

$\mathfrak{a}_{21} : 6,\ \{XX, YY, XY, YX, ZX, ZY, XI, YI, ZI, IZ\}, \{XX, YY, XY, YX, XZ, YZ, IX, IY, ZI, IZ\},$
$\{XX, ZZ, XZ, ZX, YX, YZ, XI, ZI, YI, IY\}, \{XX, ZZ, XZ, ZX, XY, ZY, IX, IZ, YI, IY\},$
$\{YY, ZZ, YZ, ZY, XY, XZ, YI, ZI, XI, IX\}, \{YY, ZZ, YZ, ZY, YX, ZX, IY, IZ, XI, IX\};$

$\mathfrak{a}_{22} : 1,\ \{XX, YY, ZZ, XY, YX, XZ, ZX, YZ, ZY, XI, IX, YI, IY, ZI, IZ\};$

$\mathfrak{b}_0 : 3,\ \{XI, IX\}, \{YI, IY\}, \{ZI, IZ\};$

$\mathfrak{b}_1 : 3,\ \{XX, XI, IX\}, \{YY, YI, IY\}, \{ZZ, ZI, IZ\};$

$\mathfrak{b}_2 : 6,\ \{XY, XZ, XI, IX\}, \{YX, YZ, YI, IY\}, \{ZX, ZY, ZI, IZ\},$
$\{YX, ZX, XI, IX\}, \{XY, ZY, YI, IY\}, \{XZ, YZ, ZI, IZ\};$

$\mathfrak{b}_3 : 1,\ \{XI, YI, ZI, IX, IY, IZ\};$

$\mathfrak{b}_4 : 6,\ \{XX, XY, XZ, XI, IX, IY, IZ\}, \{XX, YX, ZX, IX, XI, YI, ZI\},$
$\{YY, YX, YZ, YI, IY, IX, IZ\}, \{YY, XY, ZY, IY, YI, XI, ZI\},$
$\{ZZ, ZX, ZY, ZI, IZ, IX, IY\}, \{ZZ, XZ, YZ, IZ, ZI, XI, YI\};$

$\mathfrak{c}_0 : 6,\ \{XI\}, \{YI\}, \{ZI\}, \{IX\}, \{IY\}, \{IZ\};$

$\mathfrak{c}_1 : 12,\ \{XY, XI\}, \{XZ, XI\}, \{YX, YI\}, \{YZ, YI\}, \{ZX, ZI\}, \{ZY, ZI\},$
$\{YX, IX\}, \{ZX, IX\}, \{XY, IY\}, \{ZY, IY\}, \{XZ, IZ\}, \{YZ, IZ\};$

$\mathfrak{c}_2 : 6,\ \{XX, XI\}, \{XX, IX\}, \{YY, YI\}, \{YY, IY\}, \{ZZ, ZI\}, \{ZZ, IZ\};$

$\mathfrak{c}_3 : 6,\ \{XI, IY\}, \{XI, IZ\}, \{YI, IX\}, \{YI, IZ\}, \{ZI, IX\}, \{ZI, IY\};$

$\mathfrak{c}_4 : 6,\ \{XY, XI, IY\}, \{YZ, YI, IZ\}, \{ZX, ZI, IX\},$
$\{YX, YI, IX\}, \{ZY, ZI, IY\}, \{XZ, XI, IZ\};$

$\mathfrak{c}_5 : 2,\ \{XI, YI, ZI\}, \{IX, IY, IZ\};$

$\mathfrak{c}_6 : 12,\ \{XX, XY, XI, IZ\}, \{XX, XZ, XI, IY\}, \{XX, YX, IX, ZI\}, \{XX, ZX, IX, YI\},$
$\{YY, YX, YI, IZ\}, \{YY, YZ, YI, IX\}, \{YY, XY, IY, ZI\}, \{YY, ZY, IY, XI\},$
$\{ZZ, ZX, ZI, IY\}, \{ZZ, ZY, ZI, IX\}, \{ZZ, XZ, IZ, YI\}, \{ZZ, YZ, IZ, XI\};$

$\mathfrak{c}_7 : 6,\ \{XI, IX, YI, ZI\}, \{YI, IY, XI, ZI\}, \{ZI, IZ, XI, YI\},$
$\{XI, IX, IY, IZ\}, \{YI, IY, IX, IZ\}, \{ZI, IZ, IX, IY\}.$

Adding up the orders of the orbits, we obtain a total of 127 Lie algebras of type $\mathfrak{a}$, 19 of type $\mathfrak{b}$, and 56 of type $\mathfrak{c}$.

III. Identifying the subalgebras of $\mathfrak{su}(4)$ with known spin systems

In Table S.II below, we identify some of our Lie algebras from Sect. B I with the dynamical Lie algebras of known spin models. The listed generating sets are from Sect. B I. However, these generating sets are not unique, and they can be replaced with alternative generators that represent the Pauli terms of the Hamiltonian as in *2-local spin chains* in the Results section of the main text; we call those conventional generators.

**Label**	**Generating set**	**Conventional generators**	**Example Model**
$\mathfrak{a}_0$	XX	ZZ	Ising model [5]
$\mathfrak{a}_1$	XY	XY	Kitaev chain
$\mathfrak{a}_2$	XY, YX		Massless free fermion in a magnetic field
$\mathfrak{a}_3$	XX, YZ	ZZ, XY	Kitaev chain with nearest neighbor Coulomb interaction
$\mathfrak{a}_4$	XX, YY		XY-model [6] / Massless free fermion
$\mathfrak{a}_5$	XY, YZ		
$\mathfrak{a}_6$	XX, YZ, ZY	ZZ, XY, YX	Massless free fermion in a magnetic field with nearest neighbor Coulomb interaction
$\mathfrak{a}_7$	XX, YY, ZZ		Heisenberg model / XXZ Chain [6]
$\mathfrak{a}_8$	XX, XZ	ZZ, IX	Transverse-field Ising model [6]
$\mathfrak{a}_9$	XY, XZ	XY, IX	Kitaev chain in an X field
$\mathfrak{a}_{10}$	XY, YZ, ZX		Heisenberg model
$\mathfrak{a}_{11}$	XY, YX, YZ	XX, YY, IY	XY model in a Y field
$\mathfrak{a}_{12}$	XX, XY, YZ		
$\mathfrak{a}_{13}$	XX, YY, YZ	XX, YY, IX	XY-model in a longitudinal field [6]
$\mathfrak{a}_{14}$	XX, YY, XY		Transverse-field XY / Ising model [6]
$\mathfrak{a}_{15}$	XX, XY, XZ	$ZZ, IX, IY, (IZ)$	Ising model in an arbitrary magnetic field
$\mathfrak{a}_{16}$	XY, YX, YZ, ZY	XY, YX, IY, YI	Kitaev chain in a Y field
$\mathfrak{a}_{17}$	XX, XY, ZX	$ZZ, IX, IY, (IZ)$	Ising model in an arbitrary magnetic field
$\mathfrak{a}_{18}$	XX, XZ, YY, ZY	$XX, YY, IY, XI, (ZI)$	XY model in an arbitrary field
$\mathfrak{a}_{19}$	XX, XY, ZX, YZ		
$\mathfrak{a}_{20}$	XX, YY, ZZ, ZY	XX, YY, ZZ, IX, XI	XXZ chain in an X field [6]
$\mathfrak{a}_{21}$	XX, YY, XY, ZX	$XX, YY, IZ, YI, (IX)$	XY model in an arbitrary field
$\mathfrak{a}_{22}$	XX, XY, XZ, YX	ZZ, XI, IY, IZ, YI	Ising model in an arbitrary field
$\mathfrak{b}_0$	XI, IX	ZI, IZ	Uncoupled spins
$\mathfrak{b}_1$	XX, XI, IX	ZZ, ZI, IZ	Ising model [5]
$\mathfrak{b}_2$	XY, XI, IX		Kitaev chain in an X field
$\mathfrak{b}_3$	XI, YI, IX, IY		Uncoupled spins
$\mathfrak{b}_4$	XX, XY, XI, IX	ZZ, IX, IY, IZ, ZI	Ising model in an arbitrary field

Table S.II. Examples of conventional spin models corresponding to the dynamical Lie algebras discussed in the main text. Terms in parentheses do not appear explicitly in the set of generators, but are generated from them.

IV. Extending subalgebras of $\mathfrak{su}(4)$ to $\mathfrak{su}(2^n)$

In this subsection, we elaborate on the constructions of *Growing the dynamical Lie algebras* in the Results section of the main text. First, starting from an arbitrary subalgebra $\mathfrak{a} \subseteq \mathfrak{su}(4)$, we define $\mathfrak{a}(n)$ as the subalgebra of $\mathfrak{su}(2^n)$ generated by the set

$$\bigcup_{0\le k\le n-2} I^{\otimes k} \otimes \mathfrak{a} \otimes I^{\otimes(n-2-k)}. \tag{B1}$$

In particular, $\mathfrak{a}(2) = \mathfrak{a}$. The sequence of Lie algebras $\mathfrak{a}(2), \mathfrak{a}(3), \ldots$ can be determined inductively as follows.

Remark B.1. *We have two Lie algebra embeddings $\mathfrak{a}(n) \to \mathfrak{a}(n+1)$, given by appending I in the last or first qubit, and $\mathfrak{a}(n+1)$ is generated as a Lie algebra by the union of the two images:*

$$\mathfrak{a}(n+1) = \langle (\mathfrak{a}(n) \otimes I) \cup (I \otimes \mathfrak{a}(n)) \rangle_{\mathrm{Lie}}. \tag{B2}$$

Indeed, if we denote the set (B1) *by $\mathcal{A}(n)$, we have $\mathcal{A}(n+1) = (\mathcal{A}(n) \otimes I) \cup (I \otimes \mathcal{A}(n))$.*

As a consequence of the above remark, if $\mathfrak{a}(n) = \mathfrak{su}(2^n)$ for some $n = n_0 \geq 2$, then this is true for all $n \geq n_0$. For instance, since

$$\mathfrak{a}_{18}(3) = \mathfrak{a}_{19}(3) = \mathfrak{a}_{21}(3) = \mathfrak{a}_{22}(3) = \mathfrak{su}(8), \quad \mathfrak{a}_{12}(4) = \mathfrak{a}_{17}(4) = \mathfrak{su}(16), \tag{B3}$$

we obtain that

$$\mathfrak{a}_k(n) = \mathfrak{su}(2^n), \qquad k = 12, 17, 18, 19, 21, 22, \quad n \geq 4. \tag{B4}$$

Recall that in Sect. B I we considered only subalgebras $\mathfrak{a} \subseteq \mathfrak{su}(4)$ generated by a set $\mathcal{A}$ of Pauli strings. In this case, we have

$$\mathfrak{a}(n) = \langle \{ A_i B_{i+1} \,|\, AB \in \mathcal{A},\ 1 \leq i \leq n-1 \} \rangle_{\mathrm{Lie}}, \tag{B5}$$

where AB denote 2-site Pauli strings ($A, B \in \{I, X, Y, Z\}$). Equation (B5) can be used as an equivalent definition of $\mathfrak{a}(n)$ in the case where $\mathfrak{a}$ is generated by Pauli strings.

We also consider *periodic boundary conditions*. For each $n \geq 2$, define the subalgebra of $\mathfrak{su}(2^n)$:

$$\mathfrak{a}^\circ(n) = \langle \{ A_i B_{i+1}, B_1 A_n \,|\, AB \in \mathcal{A},\ 1 \leq i \leq n-1 \} \rangle_{\mathrm{Lie}}, \tag{B6}$$

again in the case where $\mathfrak{a} \subseteq \mathfrak{su}(4)$ is generated by a set $\mathcal{A}$ of Pauli strings. In order to define $\mathfrak{a}^\circ(n)$ in the general case, we introduce the cyclic shift operator $\tau_n \colon \mathfrak{su}(2^n) \to \mathfrak{su}(2^n)$, which acts on Pauli strings as

$$\tau_n(P^1 \otimes P^2 \otimes \cdots \otimes P^n) = P^2 \otimes \cdots \otimes P^n \otimes P^1, \qquad P^j \in \{I, X, Y, Z\} \tag{B7}$$

(where the superscipts are indices not powers) and is extended by linearity to the whole $\mathfrak{su}(2^n)$. Then τ_n is a Lie algebra automorphism. By definition, $\mathfrak{a}^\circ(n)$ is generated as a Lie algebra by the union of $\mathfrak{a}(n)$ and $\tau_n \mathfrak{a}(n)$:

$$\mathfrak{a}^\circ(n) = \langle \mathfrak{a}(n) \cup \tau_n \mathfrak{a}(n) \rangle_{\mathrm{Lie}}. \tag{B8}$$

Note that, by construction, $\tau_n \mathfrak{a}^\circ(n) = \mathfrak{a}^\circ(n)$ and $\tau_n \mathfrak{a}(n) \subseteq \mathfrak{a}^\circ(n)$.

We will also consider the fully connected topology corresponding to *permutation invariant* subalgebras of $\mathfrak{su}(2^n)$, i.e., invariant under the action of the symmetric group S_n that permutes the n qubits. Similarly to (B7), every permutation $\sigma \in S_n$ defines an automorphism $\sigma \colon \mathfrak{su}(2^n) \to \mathfrak{su}(2^n)$. Then we define

$$\mathfrak{a}^\pi(n) = \Big\langle \bigcup_{\sigma \in S_n} \sigma \mathfrak{a}(n) \Big\rangle_{\mathrm{Lie}}. \tag{B9}$$

In the case where $\mathfrak{a} \subseteq \mathfrak{su}(4)$ is generated by a set $\mathcal{A}$ of Pauli strings, we can write more explicitly

$$\mathfrak{a}^\pi(n) = \langle \{ A_i B_j \,|\, AB \in \mathcal{A},\ 1 \leq i \neq j \leq n \} \rangle_{\mathrm{Lie}}. \tag{B10}$$

Note that, in particular, $\mathfrak{a}^\circ(n) \subseteq \mathfrak{a}^\pi(n)$. Moreover, without loss of generality, we can assume that the generating subalgebra $\mathfrak{a} \subseteq \mathfrak{su}(4)$ is itself invariant under S_2, i.e., under the flip of the two qubits. In particular, we have:

$$\begin{aligned}
\mathfrak{a}_1^\pi(n) &= \mathfrak{a}_2^\pi(n), \\
\mathfrak{a}_3^\pi(n) &= \mathfrak{a}_6^\pi(n), \\
\mathfrak{a}_5^\pi(n) &= \mathfrak{a}_{11}^\pi(n) = \mathfrak{a}_{16}^\pi(n), \\
\mathfrak{a}_8^\pi(n) &\cong \mathfrak{a}_{14}^\pi(n), \\
\mathfrak{a}_9^\pi(n) &= \mathfrak{b}_2^\pi(n) \cong \mathfrak{a}_{16}^\pi(n), \\
\mathfrak{a}_{13}^\pi(n) &= \mathfrak{a}_{20}^\pi(n), \\
\mathfrak{a}_k^\pi(n) &= \mathfrak{b}_4^\pi(n) = \mathfrak{su}(2^n), \qquad k = 10, 12, 15, 17, 18, 19, 21, 22.
\end{aligned}$$

Thus, we only need to determine $\mathfrak{a}_k^\pi(n)$ for $k = 0, 2, 4, 6, 7, 14, 16, 20$ and $\mathfrak{b}_l^\pi(n)$ for $l = 0, 1, 3$.

V. Subalgebras of $\mathfrak{su}(8)$

For completeness and for later use, we list a linear basis (over $\mathbb{R}$, after multiplication by i) for each of the following subalgebras of $\mathfrak{su}(8)$. We have found these subalgebras from (B5), (B6) after running Algorithm 1 in the simplified form from Remark II.1, using our code [4].

Open case:

$$\begin{aligned}
\mathfrak{a}_0(3) &: \{IXX, XXI\},\\
\mathfrak{a}_1(3) &: \{IXY, XYI, XZY\},\\
\mathfrak{a}_2(3) &: \{IXY, IYX, XYI, XZY, YXI, YZX\},\\
\mathfrak{a}_3(3) &: \{IXX, IYZ, XXI, XZZ, YIY, YXZ, YYX, YZI, ZIZ, ZXY\},\\
\mathfrak{a}_4(3) &: \{IXX, IYY, XXI, XZY, YYI, YZX\},\\
\mathfrak{a}_5(3) &: \{IXY, IYZ, XYI, XZY, YIX, YXZ, YYY, YZI, ZIY, ZYX\},\\
\mathfrak{a}_6(3) &: \{IXY, IYX, IZZ, XIX, XXZ, XYI, XZY, YIY, YXI, YYZ, YZX, ZIZ, ZXX, ZYY, ZZI\},\\
\mathfrak{a}_7(3) &: \{IXX, IYY, IZZ, XIX, XXI, XYZ, XZY, YIY, YXZ, YYI, YZX, ZIZ, ZXY, ZYX, ZZI\},\\
\mathfrak{a}_8(3) &: \{IIY, IXX, IXZ, IYI, IZX, IZZ, XXI, XYX, XYZ, XZI\},\\
\mathfrak{a}_9(3) &: \{IIX, IXI, IXY, IXZ, XYI, XYY, XYZ, XZI, XZY, XZZ\},\\
\mathfrak{a}_{10}(3) &: \{IXY, IYZ, IZX, XIZ, XXX, XYI, XZY, YIX, YXZ, YYY, YZI, ZIY, ZXI, ZYX, ZZZ\},\\
\mathfrak{a}_{11}(3) &: \{IIX, IXI, IXY, IXZ, IYX, IZX, XIY, XIZ, XXX, XYI, XYY, XYZ, XZI, XZY,\\
&\quad XZZ, YXI, YYX, YZX, ZIX, ZXY, ZXZ\},\\
\mathfrak{a}_{12}(3) &: \{IIX, IIY, IIZ, IXX, IXY, IXZ, IYX, IYY, IYZ, IZI, XII, XXI, XYI, XZX, XZY,\\
&\quad XZZ, YIX, YIY, YIZ, YXX, YXY, YXZ, YYX, YYY, YYZ, YZI, ZIX, ZIY, ZIZ, ZXX,\\
&\quad ZXY, ZXZ, ZYX, ZYY, ZYZ, ZZI\},\\
\mathfrak{a}_{13}(3) &: \{IIX, IXI, IXX, IYY, IYZ, IZY, IZZ, XII, XIX, XXI, XYY, XYZ, XZY, XZZ, YIY,\\
&\quad YIZ, YXY, YXZ, YYI, YYX, YZI, YZX, ZIY, ZIZ, ZXY, ZXZ, ZYI, ZYX, ZZI, ZZX\},\\
\mathfrak{a}_{14}(3) &: \{IIZ, IXX, IXY, IYX, IYY, IZI, XXI, XYI, XZX, XZY, YXI, YYI, YZX, YZY, ZII\},\\
\mathfrak{a}_{15}(3) &: \{IIX, IIY, IIZ, IXI, IXX, IXY, IXZ, IYI, IYX, IYY, IYZ, IZI, IZX, IZY, IZZ, XIX,\\
&\quad XIY, XIZ, XXI, XXX, XXY, XXZ, XYI, XYX, XYY, XYZ, XZI, XZX, XZY, XZZ\},\\
\mathfrak{a}_{16}(3) &: \{IIX, IXI, IXY, IXZ, IYX, IZX, XII, XIY, XIZ, XXX, XYI, XYY, XYZ, XZI,\\
&\quad XZY, XZZ, YIX, YXI, YXY, YXZ, YYX, YZX, ZIX, ZXI, ZXY, ZXZ, ZYX, ZZX\},\\
\mathfrak{a}_{17}(3) &: \{IIZ, IXI, IXX, IXY, IYI, IYX, IYY, IZI, IZX, IZY, XIZ, XXI, XXX, XXY,\\
&\quad XYI, XYX, XYY, XZI, XZX, XZY, YII, YIX, YIY, YXZ, YYZ, YZZ, ZIZ, ZXI,\\
&\quad ZXX, ZXY, ZYI, ZYX, ZYY, ZZI, ZZX, ZZY\},\\
\mathfrak{a}_{20}(3) &: \{IIZ, IXX, IXY, IYX, IYY, IZI, IZZ, XIX, XIY, XXI, XXZ, XYI, XYZ, XZX, XZY,\\
&\quad YIX, YIY, YXI, YXZ, YYI, YYZ, YZX, YZY, ZII, ZIZ, ZXX, ZXY, ZYX, ZYY, ZZI\}.
\end{aligned}$$

Periodic case:

$$\begin{aligned}
\mathfrak{a}^\circ_0(3) &: \{IXX, XIX, XXI\},\\
\mathfrak{a}^\circ_1(3) &: \{IXY, XYI, XZY, YIX, YXZ, ZYX\},\\
\mathfrak{a}^\circ_2(3) &: \{IXY, IYX, XIY, XYI, XYZ, XZY, YIX, YXI, YXZ, YZX, ZXY, ZYX\},\\
\mathfrak{a}^\circ_3(3) &: \{IIX, IXI, IXX, IYY, IYZ, IZY, IZZ, XII, XIX, XXI, XYY, XYZ, XZY, XZZ, YIY,\\
&\quad YIZ, YXY, YXZ, YYI, YYX, YZI, YZX, ZIY, ZIZ, ZXY, ZXZ, ZYI, ZYX, ZZI, ZZX\},\\
\mathfrak{a}^\circ_4(3) &: \{IXX, IYY, IZZ, XIX, XXI, XYZ, XZY, YIY, YXZ, YYI, YZX, ZIZ, ZXY, ZYX, ZZI\},\\
\mathfrak{a}^\circ_6(3) &: \{IIZ, IXX, IXY, IYX, IYY, IZI, IZZ, XIX, XIY, XXI, XXZ, XYI, XYZ, XZX, XZY,\\
&\quad YIX, YIY, YXI, YXZ, YYI, YYZ, YZX, YZY, ZII, ZIZ, ZXX, ZXY, ZYX, ZYY, ZZI\},\\
\mathfrak{a}^\circ_8(3) &: \{IIY, IXX, IXZ, IYI, IYY, IZX, IZZ, XIX, XIZ, XXI, XXY, XYX, XYZ, XZI, XZY,\\
&\quad YII, YIY, YXX, YXZ, YYI, YZX, YZZ, ZIX, ZIZ, ZXI, ZXY, ZYX, ZYZ, ZZI, ZZY\},\\
\mathfrak{a}^\circ_9(3) &: \{IIX, IXI, IXY, IXZ, XII, XYI, XYY, XYZ, XZI, XZY, XZZ, YIX, YXY, YXZ, YYX,
\end{aligned}$$

$$
\begin{aligned}
&\quad YZX, ZIX, ZXY, ZXZ, ZYX, ZZX\},\\
\mathfrak{a}_{11}^{\circ}(3):\ &\{IIX, IXI, IXY, IXZ, IYX, IZX, XII, XIY, XIZ, XXX, XYI, XYY, XYZ, XZI, XZY,\\
&\quad XZZ, YIX, YXI, YXY, YXZ, YYX, YZX, ZIX, ZXI, ZXY, ZXZ, ZYX, ZZX\},\\
\mathfrak{a}_{14}^{\circ}(3):\ &\{IIZ, IXX, IXY, IYX, IYY, IZI, IZZ, XIX, XIY, XXI, XXZ, XYI, XYZ, XZX, XZY,\\
&\quad YIX, YIY, YXI, YXZ, YYI, YYZ, YZX, YZY, ZII, ZIZ, ZXX, ZXY, ZYX, ZYY, ZZI\}.
\end{aligned}
$$

Moreover, we have

$$\mathfrak{a}_k^{\circ}(3) = \mathfrak{a}_k(3), \qquad k = 5, 7, 10, 13, 16, 20,$$

and

$$\mathfrak{a}_{12}^{\circ}(3) = \mathfrak{a}_{15}^{\circ}(3) = \mathfrak{a}_{17}^{\circ}(3) = \mathfrak{su}(8).$$

VI. Subalgebras of $\mathfrak{su}(2^n)$ corresponding to open spin chains

For convenience, here we reproduce the list of subalgebras of $\mathfrak{su}(2^n)$ from Theorem IV.1, with the additional information of the dimensions of the Lie algebras (cf. (A9)). The proof of the theorem is given in Sect. C below.

$$
\begin{aligned}
\mathfrak{a}_0(n) &= \mathrm{span}\{X_jX_{j+1}\}_{1\le j\le n-1} \cong \mathfrak{u}(1)^{\oplus(n-1)}, \quad \dim = n-1,\\
\mathfrak{a}_1(n) &= \mathrm{span}\{X_iZ_{i+1}\cdots Z_{j-1}Y_j\}_{1\le i<j\le n} \cong \mathfrak{so}(n), \quad \dim = \frac{n(n-1)}{2},\\
\mathfrak{a}_2(n) &= \mathrm{span}\{X_iZ_{i+1}\cdots Z_{j-1}Y_j\}_{1\le i<j\le n} \oplus \mathrm{span}\{Y_iZ_{i+1}\cdots Z_{j-1}X_j\}_{1\le i<j\le n}\\
&\cong \mathfrak{so}(n)\oplus\mathfrak{so}(n), \quad \dim = n(n-1),\\
\mathfrak{a}_3(n) &\cong \begin{cases}
\mathfrak{so}(2^{n-2})^{\oplus 4}, & \dim = 2^{n-1}(2^{n-2}-1), \quad n\equiv 0 \mod 8,\\
\mathfrak{so}(2^{n-1}), & \dim = 2^{n-2}(2^{n-1}-1), \quad n\equiv \pm1 \mod 8,\\
\mathfrak{su}(2^{n-2})^{\oplus 2}, & \dim = 2^{2n-3}-2, \quad n\equiv \pm2 \mod 8,\\
\mathfrak{sp}(2^{n-2}), & \dim = 2^{n-2}(2^{n-1}+1), \quad n\equiv \pm3 \mod 8,\\
\mathfrak{sp}(2^{n-3})^{\oplus 4}, & \dim = 2^{n-1}(2^{n-2}+1), \quad n\equiv 4 \mod 8,
\end{cases}\\
\mathfrak{a}_4(n) &\cong \mathfrak{a}_2(n),\\
\mathfrak{a}_5(n) &\cong \begin{cases}
\mathfrak{so}(2^{n-2})^{\oplus 4}, & \dim = 2^{n-1}(2^{n-2}-1), \quad n\equiv 0 \mod 6,\\
\mathfrak{so}(2^{n-1}), & \dim = 2^{n-2}(2^{n-1}-1), \quad n\equiv \pm1 \mod 6,\\
\mathfrak{su}(2^{n-2})^{\oplus 2}, & \dim = 2^{2n-3}-2, \quad n\equiv \pm2 \mod 6,\\
\mathfrak{sp}(2^{n-2}), & \dim = 2^{n-2}(2^{n-1}+1), \quad n\equiv 3 \mod 6,
\end{cases}\\
\mathfrak{a}_6(n) &\cong \mathfrak{a}_7(n) \cong \mathfrak{a}_{10}(n) \cong \begin{cases}
\mathfrak{su}(2^{n-1}), & \dim = 2^{2n-2}-1, \quad n \text{ odd},\\
\mathfrak{su}(2^{n-2})^{\oplus 4}, & \dim = 2^{2n-2}-4, \quad n\ge 4 \text{ even},
\end{cases}\\
\mathfrak{a}_8(n) &\cong \mathfrak{so}(2n-1), \quad \dim = (n-1)(2n-1),\\
\mathfrak{a}_9(n) &\cong \mathfrak{sp}(2^{n-2}), \quad \dim = 2^{n-2}(2^{n-1}+1),\\
\mathfrak{a}_{11}(n) &= \mathfrak{a}_{16}(n) = \mathfrak{so}(2^n), \quad \dim = 2^{n-1}(2^n-1), \quad n\ge 4,\\
\mathfrak{a}_k(n) &= \mathfrak{su}(2^n), \quad \dim = 2^{2n}-1, \quad k = 12, 17, 18, 19, 21, 22, \quad n\ge 4,\\
\mathfrak{a}_{13}(n) &= \mathfrak{a}_{20}(n) \cong \mathfrak{a}_{15}(n) \cong \mathfrak{su}(2^{n-1})\oplus\mathfrak{su}(2^{n-1}), \quad \dim = 2^{2n-1}-2,\\
\mathfrak{a}_{14}(n) &\cong \mathfrak{so}(2n), \quad \dim = n(2n-1),\\
\mathfrak{b}_0(n) &= \mathrm{span}\{X_i\}_{1\le i\le n} \cong \mathfrak{u}(1)^{\oplus n}, \quad \dim = n,\\
\mathfrak{b}_1(n) &= \mathrm{span}\{X_i, X_jX_{j+1}\}_{1\le i\le n,\ 1\le j\le n-1} \cong \mathfrak{u}(1)^{\oplus(2n-1)}, \quad \dim = 2n-1,\\
\mathfrak{b}_2(n) &= \mathfrak{a}_9(n)\oplus\mathrm{span}\{X_1\} \cong \mathfrak{sp}(2^{n-2})\oplus\mathfrak{u}(1), \quad \dim = 2^{n-2}(2^{n-1}+1)+1,\\
\mathfrak{b}_3(n) &= \mathrm{span}\{X_i, Y_i, Z_i\}_{1\le i\le n} \cong \mathfrak{su}(2)^{\oplus n}, \quad \dim = 3n,\\
\mathfrak{b}_4(n) &= \mathfrak{a}_{15}(n)\oplus\mathrm{span}\{X_1\} \cong \mathfrak{su}(2^{n-1})\oplus\mathfrak{su}(2^{n-1})\oplus\mathfrak{u}(1), \quad \dim = 2^{2n-1}-1.
\end{aligned}
$$

C. PROOFS

This section contains detailed proofs of Theorems IV.1, IV.2, and IV.3. The proof of Theorem IV.1 occupies Sect. C II–C VII; its plan is outlined in Sect. C I below. The proofs of Theorems IV.2 and IV.3 utilize the results of Theorem IV.1, and are given in Sect. C VIII and C IX, respectively. For an index of each proof for each algebra, see Table S.III.

Label	Generators	Scaling	Isomorphism	Reason
$\mathfrak{a}_0$	XX	$O(n)$	$\mathfrak{u}(1)^{\oplus(n-1)}$	Sect. C I
$\mathfrak{a}_1$	XY	$O(n^2)$	$\mathfrak{so}(n)$	Sect. C III, frustration graph
$\mathfrak{a}_2$	XY, YX	$O(n^2)$	$\mathfrak{so}(n) \oplus \mathfrak{so}(n)$	Sect. C III, frustration graph
$\mathfrak{a}_3$	XX, YZ	$O(n)$	n-dependent	Lemmas C.30, C.32
$\mathfrak{a}_4$	XX, YY	$O(n^2)$	$\mathfrak{a}_2$	Sect. C II, inclusion
$\mathfrak{a}_5$	XY, YZ	$O(4^n)$	n-dependent	Lemmas C.31, C.33
$\mathfrak{a}_6$	XX, YZ, ZY	$O(4^n)$	$\mathfrak{a}_7$	Sect. C II, inclusion
$\mathfrak{a}_7$	XX, YY, ZZ	$O(4^n)$	n-dependent	Lemma C.29
$\mathfrak{a}_8$	XX, XZ	$O(n^2)$	$\mathfrak{so}(2n-1)$	Sect. C III, frustration graph
$\mathfrak{a}_9$	XY, XZ	$O(4^n)$	$\mathfrak{sp}(2^{n-2})$	Lemma C.28, fixed point under involution
$\mathfrak{a}_{10}$	XY, YZ, ZX	$O(4^n)$	$\mathfrak{a}_6$	Sect. C II, inclusion
$\mathfrak{a}_{11}$	XY, YX, YZ	$O(4^n)$	$\mathfrak{a}_{16}$	Sect. C II, inclusion
$\mathfrak{a}_{12}$	XX, XY, YZ	$O(4^n)$	$\mathfrak{su}(2^n)$	Sect. B IV, explicit for $n = 4$
$\mathfrak{a}_{13}$	XX, YY, YZ	$O(4^n)$	$\mathfrak{su}(2^{n-1}) \oplus \mathfrak{su}(2^{n-1})$	Lemma C.27, fixed point under involution
$\mathfrak{a}_{14}$	XX, YY, XY	$O(n^2)$	$\mathfrak{so}(2n)$	Sect. C III, frustration graph
$\mathfrak{a}_{15}$	XX, XY, XZ	$O(4^n)$	$\mathfrak{a}_{13}$	Lemma C.27, fixed point under involution
$\mathfrak{a}_{16}$	XY, YX, YZ, ZY	$O(4^n)$	$\mathfrak{so}(2^n)$	Lemma C.25, fixed point under involution
$\mathfrak{a}_{17}$	XX, XY, ZX	$O(4^n)$	$\mathfrak{su}(2^n)$	Sect. B IV, explicit for $n = 4$
$\mathfrak{a}_{18}$	XX, XZ, YY, ZY	$O(4^n)$	$\mathfrak{su}(2^n)$	Sect. B IV, explicit for $n = 3$
$\mathfrak{a}_{19}$	XX, XY, ZX, YZ	$O(4^n)$	$\mathfrak{su}(2^n)$	Sect. B IV, explicit for $n = 3$
$\mathfrak{a}_{20}$	XX, YY, ZZ, ZY	$O(4^n)$	$\mathfrak{a}_{13}$	Sect. C II, inclusion
$\mathfrak{a}_{21}$	XX, YY, XY, ZX	$O(4^n)$	$\mathfrak{su}(2^n)$	Sect. B IV, explicit for $n = 3$
$\mathfrak{a}_{22}$	XX, XY, XZ, YX	$O(4^n)$	$\mathfrak{su}(2^n)$	Sect. B IV, explicit for $n = 3$
$\mathfrak{b}_0$	XI, IX	$O(n)$	$\mathfrak{u}(1)^{\oplus n}$	Sect. C I
$\mathfrak{b}_1$	XX, XI, IX	$O(n)$	$\mathfrak{u}(1)^{\oplus(2n-1)}$	Sect. C I
$\mathfrak{b}_2$	XY, XI, IX	$O(4^n)$	$\mathfrak{sp}(2^{n-2}) \oplus \mathfrak{u}(1)$	Sect. C I, central extension
$\mathfrak{b}_3$	XI, YI, IX, IY	$O(n)$	$\mathfrak{su}(2)^{\oplus n}$	Sect. C I
$\mathfrak{b}_4$	$XX, XY, XZ, XI, IX, IY, IZ$	$O(4^n)$	$\mathfrak{su}(2^{n-1}) \oplus \mathfrak{su}(2^{n-1}) \oplus \mathfrak{u}(1)$	Sect. C I, central extension

Table S.III. Proofs and where to find them.

I. Plan of the proof of Theorem IV.1

Our starting point is the list of subalgebras of $\mathfrak{su}(4)$ from Sect. B I:

$$\mathfrak{a}_k, \mathfrak{b}_l \subseteq \mathfrak{su}(4), \qquad 0 \le k \le 22, \;\; 0 \le l \le 4, \tag{C1}$$

and the goal is to determine (up to isomorphism) their extensions as subalgebras of $\mathfrak{su}(2^n)$:

$$\mathfrak{a}_k(n), \mathfrak{b}_l(n) \subseteq \mathfrak{su}(2^n), \qquad 3 \le n, \;\; 0 \le k \le 22, \;\; 0 \le l \le 4, \tag{C2}$$

which are defined in Sect. B IV. The answer is presented in Theorem IV.1, and in more detail, in Sect. B VI above. Here we outline the proof, which consists of multiple parts. We divide the set of Lie algebras (C2) into three classes: linear, quadratic, and exponential, according to the anticipated growth of their dimension.

Linear case: $\mathfrak{a}_0(n)$, $\mathfrak{b}_l(n)$ $(l = 0, 1, 3)$. The linear case is obvious. Indeed, the Lie algebras $\mathfrak{a}_0(n)$, $\mathfrak{b}_0(n)$, and $\mathfrak{b}_1(n)$ are Abelian (i.e., have identically zero Lie brackets), because all of their generators commute with each other. The Lie algebra $\mathfrak{b}_3(n) \cong \mathfrak{su}(2)^{\oplus n}$ is a direct sum of n commuting copies of $\mathfrak{su}(2)$, since its generators split into n groups acting independently on each qubit and commuting with each other.

Quadratic case: $\mathfrak{a}_k(n)$ $(k = 1, 2, 4, 8, 14)$. These Lie algebras are determined by using the *frustration graphs* of their generators; see Sect. C III and especially Lemma C.6. Note that $\mathfrak{a}_2(n) \cong \mathfrak{a}_4(n)$ by Lemma C.3.

Exponential case: $\mathfrak{a}_k(n)$, $\mathfrak{b}_l(n)$ $(k = 3, 5, 6, 7, 9–13, 15–22,\ l = 2, 4)$. First, recall that in Sect. B IV, we have already found that

$$\mathfrak{a}_k(n) = \mathfrak{su}(2^n), \qquad k = 12, 17, 18, 19, 21, 22, \quad n \geq 4. \tag{C3}$$

Second, we observe that $\mathfrak{b}_2(n) = \mathfrak{a}_9(n) \oplus \operatorname{span}\{X_1\}$ and $\mathfrak{b}_4(n) = \mathfrak{a}_{15}(n) \oplus \operatorname{span}\{X_1\}$, because their generators consist of a central element X_1 (commuting with all other generators) and the generators of $\mathfrak{a}_9(n)$ or $\mathfrak{a}_{15}(n)$, respectively. Third, in Sect. C II, we find equalities and isomorphisms among some of the Lie algebras $\mathfrak{a}_k(n)$. Namely,

$$\mathfrak{a}_6(n) \cong \mathfrak{a}_7(n) \cong \mathfrak{a}_{10}(n), \qquad \mathfrak{a}_{11}(n) = \mathfrak{a}_{16}(n), \qquad \mathfrak{a}_{13}(n) = \mathfrak{a}_{20}(n), \qquad n \geq 4. \tag{C4}$$

Thus, we are left to investigate the Lie algebras $\mathfrak{a}_k(n)$ for $k = 3, 5, 7, 9, 13, 15, 16$.

Strategy for $\mathfrak{a}_k(n)$ $(k = 3, 5, 7, 9, 13, 15, 16)$. The strategy in the remaining exponential cases is as follows.

1. For each of our Lie algebras $\mathfrak{s} = \mathfrak{a}_k(n)$, we find its *stabilizer* $\operatorname{St}(\mathfrak{s})$, which is defined as the set of all Pauli strings $\in \mathcal{P}_n$ that commute with every element of $\mathfrak{s}$. This can be done explicitly, because the stabilizer is determined only from the generators of $\mathfrak{s}$; see Proposition C.3 in Sect. C IV.

2. By definition, $\mathfrak{s}$ commutes with all elements of its stabilizer $\operatorname{St}(\mathfrak{s})$; hence, it is contained in the *centralizer* of $\operatorname{St}(\mathfrak{s})$ in $\mathfrak{su}(2^n)$, which we denote $\mathfrak{su}(2^n)^{\operatorname{St}(\mathfrak{s})}$. We can reduce the Lie subalgebra $\mathfrak{su}(2^n)^{\operatorname{St}(\mathfrak{s})}$ further by factoring all elements of the center of $\operatorname{St}(\mathfrak{s})$, which will become central in it, because $\mathfrak{s}$ has a trivial center by Lemma C.12. This results in a Lie algebra denoted $\mathfrak{g}_k(n)$ when $\mathfrak{s} = \mathfrak{a}_k(n)$; these are listed explicitly in (C25)–(C31).

3. By the above construction, we have $\mathfrak{s} \subseteq \mathfrak{g}_k(n)$. However, equality does not hold in all cases. We improve the upper bound for $\mathfrak{s}$ by finding an *involution* θ_k of $\mathfrak{g}_k(n)$ such that all elements of $\mathfrak{s}$ are fixed under θ_k, i.e., $\theta_k(a) = a$ for all $a \in \mathfrak{s}$ (see Sect. A III for a refresher on involutions). The last condition can be checked only on the generators of $\mathfrak{s}$, and the details are carried out in Sect. C V (Theorem C.1 and Lemmas C.14, C.15, C.16). Thus, we have the upper bound $\mathfrak{s} \subseteq \mathfrak{g}_k(n)^{\theta_k}$, where the superscript θ_k indicates fixed points under θ_k.

4. Then, in Sect. C VI, we establish a lower bound for $\mathfrak{s}$, i.e., we prove that the upper bound is exact: $\mathfrak{a}_k(n) = \mathfrak{g}_k(n)^{\theta_k}$. The main idea is to start with an arbitrary Pauli string $\in i\mathcal{P}_n \cap \mathfrak{g}_k(n)^{\theta_k}$ and use suitable commutators with elements of $\mathfrak{a}_k(n)$ to produce a Pauli string $\in i\mathcal{P}_n \cap \mathfrak{g}_k(n)^{\theta_k}$ with I in one of its positions. Erasing the I gives an element of $\mathfrak{g}_k(n-1)^{\theta_k}$, which by induction is in $\mathfrak{a}_k(n-1)$. The specific details are broken into a sequence of lemmas. The cases $k = 3, 5, 7$, $k = 9$, $k = 13$, $k = 15$, and $k = 16$ are treated in Lemmas C.21, C.23, C.24, C.26, and C.25, respectively. As a consequence, since $\mathfrak{g}_{16}(n) = \mathfrak{su}(2^n)$ by (C30), and $\theta_{16}(g) = -g^T$ by (C36), we obtain that $\mathfrak{a}_{16}(n) = \mathfrak{so}(2^n)$.

5. Finally, in Sect. C VII, we identify the Lie algebras $\mathfrak{g}_k(n)^{\theta_k}$ with those from Theorem IV.1. The idea is to apply a suitable unitary transformation that brings the stabilizer $\operatorname{St}(\mathfrak{s})$ to a more convenient form (such transformations and their effect on the fixed points of involutions are reviewed in Sect. A III). For example, $\operatorname{St}(\mathfrak{a}_{13}(n)) = \{I^{\otimes n}, X^{\otimes n}\}$ and we can bring $X^{\otimes n}$ to X_1 with a unitary transformation, after which it is easy to determine the centralizer $\mathfrak{su}(2^n)^{X_1}$. This is carried out in Lemmas C.27, C.28, and C.29 for $k = 13, 15$, $k = 9$, and $k = 7$, respectively. The more complicated cases $k = 3$ and $k = 5$ are further broken down to n odd and n even; see Lemmas C.30, C.32, C.31, C.33. Taken all together, this completes the proof of Theorem IV.1.

II. Inclusions and isomorphisms

There are several obvious inclusions among the Lie algebras $\mathfrak{a}_k$ $(k = 0–11, 13–16, 20)$, which extend to the corresponding subalgebras of $\mathfrak{su}(2^n)$ due to the following trivial observation.

Lemma C.1. *Consider two arbitrary subalgebras $\mathfrak{a} \subseteq \mathfrak{b} \subseteq \mathfrak{su}(4)$. Then $\mathfrak{a}(n) \subseteq \mathfrak{b}(n)$, $\mathfrak{a}^\circ(n) \subseteq \mathfrak{b}^\circ(n)$ and $\mathfrak{a}^\pi(n) \subseteq \mathfrak{b}^\pi(n)$ for all $n \geq 2$. Furthermore, if $\mathfrak{a}(n_0) = \mathfrak{b}(n_0)$ for some n_0, then $\mathfrak{a}(n) = \mathfrak{b}(n)$ for all $n \geq n_0$.*

Proof. If $\mathfrak{a} \subseteq \mathfrak{b}$, then the generating set (B1) of $\mathfrak{a}(n)$ is contained in the corresponding generating set of $\mathfrak{b}(n)$; hence, $\mathfrak{a}(n) \subseteq \mathfrak{b}(n)$. From here, we also get $\tau_n \mathfrak{a}(n) \subseteq \tau_n \mathfrak{b}(n)$, which implies $\mathfrak{a}^\circ(n) \subseteq \mathfrak{b}^\circ(n)$ due to (B8). Similarly, from (B9), we derive $\mathfrak{a}^\pi(n) \subseteq \mathfrak{b}^\pi(n)$. The last claim of the lemma follows from the inductive construction of the Lie algebras $\mathfrak{a}(n)$ given in (B2). □

For the rest of this subsection, we focus on the open case; we will treat the periodic (closed) case and the permutation-invariant case later. By comparing the bases of the subalgebras $\mathfrak{a}_k \subset \mathfrak{su}(4)$ listed in Table S.I, we notice the following inclusions:

$$\begin{aligned}
&\mathfrak{a}_0 \subset \mathfrak{a}_3 \subset \mathfrak{a}_6 \subset \mathfrak{a}_{20}, && \mathfrak{a}_1 \subset \mathfrak{a}_2 \subset \mathfrak{a}_{11} \subset \mathfrak{a}_{16}, \\
&\mathfrak{a}_0 \subset \mathfrak{a}_4 \subset \mathfrak{a}_7, && \mathfrak{a}_1 \subset \mathfrak{a}_2 \subset \mathfrak{a}_{14}, \\
&\mathfrak{a}_0 \subset \mathfrak{a}_4 \subset \mathfrak{a}_{13} \subset \mathfrak{a}_{20}, && \mathfrak{a}_1 \subset \mathfrak{a}_5 \subset \mathfrak{a}_{10}, \quad \text{and} \quad \mathfrak{a}_1 \subset \mathfrak{a}_5 \subset \mathfrak{a}_{16}, \\
&\mathfrak{a}_0 \subset \mathfrak{a}_8 \subset \mathfrak{a}_{15}, && \mathfrak{a}_1 \subset \mathfrak{a}_9 \subset \mathfrak{a}_{15}.
\end{aligned}$$

The above chains of inclusions are maximal, i.e., cannot be extended further. By Lemma C.1, for any pair $\mathfrak{a}_k \subset \mathfrak{a}_l$, we get an inclusion $\mathfrak{a}_k(n) \subseteq \mathfrak{a}_l(n)$ for all $n \geq 3$. However, observe that even though $\mathfrak{a}_5 = \mathfrak{a}_{10} \cap \mathfrak{a}_{16}$, we only have $\mathfrak{a}_5(n) \subseteq \mathfrak{a}_{10}(n) \cap \mathfrak{a}_{16}(n)$ and not necessarily an equality. In fact, one checks that $IZX \in \mathfrak{a}_{10}(3) \cap \mathfrak{a}_{16}(3)$ but $IZX \notin \mathfrak{a}_5(3)$. In the next lemma, we present two consequences of the above inclusions.

Lemma C.2. *We have:*

$$\mathfrak{a}_{11}(n) = \mathfrak{a}_{16}(n), \qquad n \geq 4, \tag{C5}$$
$$\mathfrak{a}_{13}(n) = \mathfrak{a}_{20}(n), \qquad n \geq 3. \tag{C6}$$

Proof. Since $\mathfrak{a}_{11} \subset \mathfrak{a}_{16}$, we have $\mathfrak{a}_{11}(n) \subseteq \mathfrak{a}_{16}(n)$ for all n. But because $\dim \mathfrak{a}_{11}(4) = \dim \mathfrak{a}_{16}(4) = 120$, we obtain that $\mathfrak{a}_{11}(4) = \mathfrak{a}_{16}(4)$, which implies (C5). Similarly, from $\mathfrak{a}_{13} \subset \mathfrak{a}_{20}$, we get $\mathfrak{a}_{13}(n) \subseteq \mathfrak{a}_{20}(n)$ for all n. But because $\dim \mathfrak{a}_{13}(3) = \dim \mathfrak{a}_{20}(3) = 30$, we have $\mathfrak{a}_{13}(3) = \mathfrak{a}_{20}(3)$. □

There are other inclusions among the Lie algebras $\mathfrak{a}_k$ after we relabel some of the Paulis. Such relabelings act as automorphisms of the Lie algebra $\mathfrak{su}(2^n)$, i.e., they are invertible linear operator that respects the Lie bracket (see Sect. A III). We will express them in the form $\varphi(a) = UaU^\dagger$ ($a \in \mathfrak{su}(2^n)$) for some fixed unitary matrix U, as in Lemma A.4.

As a first example, consider the linear operator ψ on $\mathbb{C}^{2\times 2}$, defined by $\psi(A) = VAV^\dagger$ where $V = e^{i\frac{\pi}{4}Z}$. Using (A6), we find:

$$\psi(I) = I, \qquad \psi(X) = iZ \cdot X = -Y, \qquad \psi(Y) = iZ \cdot Y = X, \qquad \psi(Z) = Z.$$

We extend it to an automorphism ψ_n of $\mathfrak{su}(2^n)$ by:

$$\psi_n := \psi^0 \otimes \psi^1 \otimes \cdots \otimes \psi^{n-1},$$

where ψ^j denotes the j-th power of ψ. Note that, up to an overall sign, ψ_n swaps $X \rightleftharpoons Y$ on all even qubits. It can be represented as a unitary transformation:

$$\psi_n(a) = UaU^\dagger, \qquad \text{with} \quad U = V^0 \otimes V^1 \otimes \cdots \otimes V^{n-1} = \exp\Big(i\frac{\pi}{4}\sum_{j=1}^{n}(j-1)Z_j\Big). \tag{C7}$$

Lemma C.3. *The map ψ_n, defined by (C7), restricts to an isomorphism $\mathfrak{a}_2(n) \cong \mathfrak{a}_4(n)$.*

Proof. Since ψ_n is an automorphisms of $\mathfrak{su}(2^n)$, it is in particular injective and respects the Lie bracket. The same is true for the restriction of ψ_n to $\mathfrak{a}_2(n)$. In order to prove that ψ_n is an isomorphism from $\mathfrak{a}_2(n)$ to $\mathfrak{a}_4(n)$, it remains to show that it is surjective, i.e., $\psi_n \mathfrak{a}_2(n) = \mathfrak{a}_4(n)$. Note that $\psi_n \mathfrak{a}_2(n)$ is a subalgebra of $\mathfrak{a}_4(n)$. We will show that $\psi_n \mathfrak{a}_2(n)$ contains all generators of $\mathfrak{a}_4(n)$, which would imply that it is equal to it.

Indeed, ψ_n acts as follows on the generators of $\mathfrak{a}_2(n)$:

$$\begin{aligned}
\psi_n(X_i Y_{i+1}) &= (\psi^{i-1}(X))_i (\psi^i(Y))_{i+1} = (\psi^{i-1}(X))_i (\psi^{i-1}(X))_{i+1}, \\
\psi_n(Y_i X_{i+1}) &= (\psi^{i-1}(Y))_i (\psi^i(X))_{i+1} = -(\psi^{i-1}(Y))_i (\psi^{i-1}(Y))_{i+1}.
\end{aligned}$$

Hence, up to a sign, ψ_n sends the generators of $\mathfrak{a}_2(n)$ to the generators $X_i X_{i+1}$, $Y_i Y_{i+1}$ of $\mathfrak{a}_4(n)$. Therefore, $\psi_n \mathfrak{a}_2(n) = \mathfrak{a}_4(n)$, which completes the proof of the lemma. □

As another similar example, consider the linear operator φ on $\mathbb{C}^{2\times 2}$, defined by

$$\varphi(A) := e^{i\frac{\pi}{4}X} A e^{-i\frac{\pi}{4}X} \quad \Rightarrow \quad \varphi(I) = I, \quad \varphi(X) = X, \quad \varphi(Y) = -Z, \quad \varphi(Z) = Y. \tag{C8}$$

We extend it to an automorphism of $\mathfrak{su}(2^n)$ by

$$\varphi_n := \varphi^0 \otimes \varphi^1 \otimes \cdots \otimes \varphi^{n-1}, \tag{C9}$$

which, up to a sign, swaps $Y \rightleftharpoons Z$ on all even qubits. As in (C7), we have

$$\varphi_n(a) = UaU^\dagger, \qquad \text{with} \quad U = \exp\Big(i\frac{\pi}{4}\sum_{j=1}^{n}(j-1)X_j\Big). \tag{C10}$$

Lemma C.4. *The map φ_n, defined by (C8), (C9), restricts to an isomorphism $\mathfrak{a}_6(n) \cong \mathfrak{a}_7(n)$.*

Proof. As in the proof of Lemma C.3, we find that φ_n acts on the generators of $\mathfrak{a}_6(n)$ as follows:

$$\begin{aligned} \varphi_n(X_iX_{i+1}) &= X_iX_{i+1}, \\ \varphi_n(Y_iZ_{i+1}) &= (\varphi^{i-1}(Y))_i(\varphi^{i-1}(Y))_{i+1}, \\ \varphi_n(Z_iY_{i+1}) &= -(\varphi^{i-1}(Z))_i(\varphi^{i-1}(Z))_{i+1}. \end{aligned}$$

Up to a sign, the images are exactly the generators X_iX_{i+1}, Y_iY_{i+1}, Z_iZ_{i+1} of $\mathfrak{a}_7(n)$. Hence, $\varphi_n\mathfrak{a}_6(n) = \mathfrak{a}_7(n)$. □

Now consider the composition $\gamma := \varphi\psi$, which acts as a cyclic rotation $X \mapsto Z \mapsto Y \mapsto X$:

$$\gamma(I) = I, \qquad \gamma(X) = Z, \qquad \gamma(Y) = X, \qquad \gamma(Z) = Y. \tag{C11}$$

We extend it to automorphism of $\mathfrak{su}(2^n)$ as follows:

$$\gamma_n := \gamma^1 \otimes \gamma^2 \otimes \gamma^3 \otimes \cdots \otimes \gamma^n. \tag{C12}$$

Since $S := (X+Y+Z)/\sqrt{3}$ satisfies $S \cdot S = I$, we can apply Euler's formula (A3) to show that

$$\gamma(A) = e^{i\frac{\pi}{4}X}e^{i\frac{\pi}{4}Z}Ae^{-i\frac{\pi}{4}Z}e^{-i\frac{\pi}{4}X} = e^{i\frac{\pi}{3}S}Ae^{-i\frac{\pi}{3}S}.$$

Hence, similarly to (C7), (C10), we can express γ_n as

$$\gamma_n(a) = UaU^\dagger, \qquad \text{with} \quad U = \exp\Big(i\frac{\pi}{3\sqrt{3}}\sum_{j=1}^{n} j(X_j+Y_j+Z_j)\Big). \tag{C13}$$

Lemma C.5. *The map γ_n, defined by (C11), (C12), restricts to an isomorphism $\mathfrak{a}_{10}(n) \cong \mathfrak{a}_7(n)$.*

Proof. We find that γ_n acts on the generators of $\mathfrak{a}_{10}(n)$ as follows:

$$\begin{aligned} \gamma_n(X_iY_{i+1}) &= (\gamma^i(X))_i(\gamma^{i+1}(Y))_{i+1} = (\gamma^i(X))_i(\gamma^i(X))_{i+1}, \\ \gamma_n(Y_iZ_{i+1}) &= (\gamma^i(Y))_i(\gamma^{i+1}(Z))_{i+1} = (\gamma^i(Y))_i(\gamma^i(Y))_{i+1}, \\ \gamma_n(Z_iX_{i+1}) &= (\gamma^i(Z))_i(\gamma^{i+1}(X))_{i+1} = (\gamma^i(Z))_i(\gamma^i(Z))_{i+1}. \end{aligned}$$

The images are exactly the generators X_iX_{i+1}, Y_iY_{i+1}, Z_iZ_{i+1} of $\mathfrak{a}_7(n)$; hence, $\gamma_n\mathfrak{a}_{10}(n) = \mathfrak{a}_7(n)$. □

III. Frustration graphs

In this subsection, we review the notion of frustration graph, which is a useful visualization tool; see e.g. [7–9]. We determine the dynamical Lie algebra in the cases when the frustration graph is a line or a circle, and apply these results to identify several of our Lie algebras, namely, $\mathfrak{a}_k(n)$ and $\mathfrak{a}_k^\circ(n)$ for $k = 1, 2, 4, 8, 14$.

Definition C.1. *Given a set of Pauli strings $\mathcal{A} \subset \mathcal{P}_n$, its* frustration graph *is the graph with a set of vertices $\mathcal{A}$ and edges connecting all pairs of vertices $a, b \in \mathcal{A}$ such that $[a, b] \neq 0$. The Lie algebra $\langle\mathcal{A}\rangle_{\text{Lie}}$ generated by $\mathcal{A}$ (cf. Definition A.1) will be called the* dynamical Lie algebra (DLA) *of the corresponding frustration graph.*

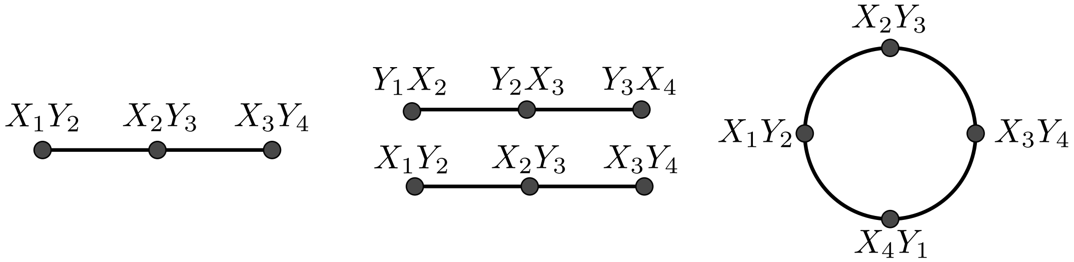

(a) $\mathfrak{a}_1(4)$ (b) $\mathfrak{a}_2(4)$ (c) $\mathfrak{a}_2^{\circ}(4)$

Figure S1. **Frustration graphs for several examples in our classification for** $n = 4$**.** (a) A frustration graph given by a line. (b) A frustration graph consisting of two disjoint lines. (c) A circular frustration graph.

The frustration graph makes it especially easy to determine when two subsets of the generating set $\mathcal{A}$ commute with each other: it means that they are disconnected from each other in the frustration graph. Suppose that $\mathcal{A} = \mathcal{A}_1 \sqcup \mathcal{A}_2$ is a disjoint union of subsets with disconnected frustration graphs. Then the DLA

$$\langle \mathcal{A}_1 \sqcup \mathcal{A}_2 \rangle_{\text{Lie}} = \langle \mathcal{A}_1 \rangle_{\text{Lie}} \oplus \langle \mathcal{A}_2 \rangle_{\text{Lie}} \tag{C14}$$

is a direct sum of commuting subalgebras. This is illustrated in Figure S1.

In the next proposition, we determine the DLA of a line frustration graph.

Proposition C.1. *Suppose that the frustration graph of* $\mathcal{A} = \{a_1, \dots, a_N\} \subset \mathcal{P}_n$ *is a line with* N *vertices, so that* $[a_j, a_k] \neq 0$ *for* $1 \le j < k \le N$ *if and only if* $k = j + 1$*. Then*

$$\langle \mathcal{A} \rangle_{\text{Lie}} \cong \mathfrak{so}(N+1),$$

and a basis for it is given by $\{i^{k-j} L_{j,k}\}_{1 \le j < k \le N+1}$*, where* i *is the imaginary unit and*

$$L_{j,k} := a_j \cdot a_{j+1} \cdots a_{k-1} \qquad (1 \le j < k \le N+1) \tag{C15}$$

are products over line segments.

Proof. Recall that $\langle \mathcal{A} \rangle_{\text{Lie}}$ is the subalgebra of $\mathfrak{su}(2^n)$ generated by the subset $i\mathcal{A} \subset \mathfrak{su}(2^n)$ (see Definition A.1). First, let us us prove that all $i^{k-j} L_{j,k}$ are in $\langle \mathcal{A} \rangle_{\text{Lie}}$. For $k = j+1$, we have $iL_{j,j+1} := ia_j \in i\mathcal{A} \subseteq \langle \mathcal{A} \rangle_{\text{Lie}}$. Suppose, by induction on $k - j$, that $i^{k-j} L_{j,k} \in \langle \mathcal{A} \rangle_{\text{Lie}}$ for some $1 < j < k \le N+1$; then we will show that $i^{k-j+1} L_{j-1,k} \in \langle \mathcal{A} \rangle_{\text{Lie}}$. By definition,

$$L_{j-1,k} = a_{j-1} \cdot a_j \cdot a_{j+1} \cdots a_{k-1} = a_{j-1} \cdot L_{j,k},$$

and by assumption, a_{j-1} anticommutes with a_j and commutes with $a_{j+1}, \dots, a_{k-1}$. Hence, a_{j-1} anticommutes with $L_{j,k}$, which implies that

$$2i^{k-j+1} L_{j-1,k} = 2i^{k-j+1} a_{j-1} \cdot L_{j,k} = i^{k-j+1} [a_{j-1}, L_{j,k}] = [ia_{j-1}, i^{k-j} L_{j,k}] \in \langle \mathcal{A} \rangle_{\text{Lie}}.$$

This proves the claim that $i^{k-j} L_{j,k} \in \langle \mathcal{A} \rangle_{\text{Lie}}$ for all $1 \le j < k \le N+1$.

Similarly to above, one can check that $(1 \le j < k < l \le N+1)$:

$$[i^{k-j} L_{j,k}, i^{l-k} L_{k,l}] = 2i^{l-j} L_{j,l}, \quad [i^{l-k} L_{k,l}, i^{l-j} L_{j,l}] = 2i^{k-j} L_{j,k}, \quad [i^{l-j} L_{j,l}, i^{k-j} L_{j,k}] = 2i^{l-k} L_{k,l}, \tag{C16}$$

and all other commutators (not following from skewsymmetry) are zero. In particular, the real linear span of all $i^{k-j} L_{j,k}$ is closed under the bracket, i.e., is a subalgebra of $\mathfrak{su}(2^n)$. Since $\langle \mathcal{A} \rangle_{\text{Lie}}$ is the minimal (under inclusion) subalgebra of $\mathfrak{su}(2^n)$ containing $i\mathcal{A}$, it follows that

$$\langle \mathcal{A} \rangle_{\text{Lie}} = \text{span}\{i^{k-j} L_{j,k}\}_{1 \le j < k \le N+1}.$$

Recall that $\mathfrak{so}(N+1) = \mathfrak{so}(N+1, \mathbb{R})$ is the Lie algebra of all skew-symmetric $(N+1) \times (N+1)$ real matrices; see (A7). Consider the standard basis $\{E_{j,k}\}_{1 \le j,k \le N+1}$ of $\mathfrak{gl}(N+1, \mathbb{R})$, where $E_{j,k}$ is the matrix with (j,k)-entry $= 1$ and all other entries $= 0$. Then a basis for $\mathfrak{so}(N+1)$ is $\{F_{j,k} := E_{j,k} - E_{k,j}\}_{1 \le j < k \le N+1}$. Using that

$$[E_{j,k}, E_{l,m}] = \delta_{k,l} E_{j,m} - \delta_{j,m} E_{l,k},$$

it is easy to see that

$$[F_{j,k}, F_{k,l}] = F_{j,l}, \quad [F_{k,l}, F_{j,l}] = F_{j,k}, \quad [F_{j,l}, F_{j,k}] = F_{k,l}, \quad \text{for} \quad 1 \le j < k < l \le N+1.$$

Hence, the matrices $2F_{j,k}$ satisfy the same commutation relations as $i^{k-j}L_{j,k}$ given in (C16). This means that the map $\mathfrak{so}(N+1) \to \langle \mathcal{A} \rangle_{\text{Lie}}$ that sends $2F_{j,k}$ to $i^{k-j}L_{j,k}$ is a Lie algebra homomorphism. Its kernel is an ideal in $\mathfrak{so}(N+1)$, but since $\mathfrak{so}(N+1)$ is simple, it has no non-zero proper ideals. Therefore, this map is an isomorphism. □

Remark C.1. *One can see from the above proposition that, for linear frustration graphs, the dimension of the DLA scales quadratically with the number of generators. This was observed for free fermionic models in [10–14], where the number of generators is proportional to the system size and the circuit gate complexity is quadratic with respect to the system size. These models are fast forwardable along with the other Hamiltonians given in [7–9], and the fundamental reason for this is the polynomial scaling of the DLA.*

Remark C.2. *After a Jordan–Wigner transformation, it can be shown that the algebra of free fermions on n sites can be generated by $\{Z_1, X_1X_2, Z_2, X_2X_3, Z_3, \dots, X_{n-1}X_n, Z_n\}$, which will be shown to be equivalent to $\mathfrak{a}_{14}(n)$ in Lemma C.6 below. These generators have a linear frustration graph with $2n-1$ vertices; hence, its DLA is $\mathfrak{so}(2n)$.*

Next, we consider the case where the frustration graph is a circle.

Proposition C.2. *Suppose that the frustration graph of $\mathcal{A} = \{a_1, \dots, a_N\} \subset \mathcal{P}_n$ is a circle with $N \ge 3$ vertices, so that $[a_j, a_k] \ne 0$ for $1 \le j < k \le N$ if and only if $k = j+1$ or $j = 1$, $k = N$. Then*

$$\langle \mathcal{A} \rangle_{\text{Lie}} \cong \mathfrak{so}(N) \oplus \mathfrak{so}(N),$$

and it has a basis $\{i^{k-j}L_{j,k}, i^{N+k-j}C \cdot L_{j,k}\}_{1 \le j<k \le N}$, where i is the imaginary unit, $L_{j,k}$ are defined in (C15), and

$$C := a_1 \cdot a_2 \cdots a_{N-1} \cdot a_N. \tag{C17}$$

Proof. First, notice that $[C, a_j] = 0$ for all $1 \le j \le N$, because a_j does not commute only with its two neighboring vertices in the circle frustration graph. Moreover, using that $a_j \cdot a_j = I^{\otimes n}$ (cf. Lemma A.1), we get $C \cdot C = (-1)^N I^{\otimes n}$. From here, we deduce that

$$[i^N C, i^{k-j}L_{j,k}] = 0, \qquad (i^N C)\cdot(i^N C) = I^{\otimes n}. \tag{C18}$$

If we remove any vertex from the frustration graph of $\mathcal{A}$, we obtain a line frustration graph. By Proposition C.1, we know that

$$i^{k-j}L_{j,k} \in \langle a_1, \dots, a_{N-1} \rangle_{\text{Lie}} \subseteq \langle \mathcal{A} \rangle_{\text{Lie}}, \qquad 1 \le j < k \le N,$$

and these elements form a basis for the subalgebra $\langle a_1, \dots, a_{N-1} \rangle_{\text{Lie}} \cong \mathfrak{so}(N)$. In particular, we have

$$\{ia_1, \dots, ia_{N-1}\} \subset \langle a_1, \dots, a_{N-1} \rangle_{\text{Lie}} = \operatorname{span}_{\mathbb{R}}\{i^{k-j}L_{j,k}\}_{1 \le j<k \le N}.$$

Similarly, the set $\mathcal{A} \setminus \{a_{k-1}\} = \{a_k, a_{k+1}, \dots, a_N, a_1, \dots, a_{k-2}\}$ has a line frustration graph and its subset $\{a_k, a_{k+1}, \dots, a_N, a_1, \dots, a_{j-1}\}$ is a line segment for $1 \le j < k \le N$. Hence, again by Proposition C.1,

$$i^{N+k-j}C \cdot L_{j,k} = \pm i^{N-k+j} a_k \cdot a_{k+1} \cdots a_N \cdot a_1 \cdots a_{j-1} \in \langle \mathcal{A} \setminus \{a_{k-1}\} \rangle_{\text{Lie}} \subseteq \langle \mathcal{A} \rangle_{\text{Lie}}, \qquad 1 \le j < k \le N.$$

In particular, the choice $j = 1$, $k = N$ gives

$$i^{N+N-1}C \cdot L_{1,N} = \pm i a_N.$$

The above discussion implies that

$$i\mathcal{A} \subset \mathcal{L} := \operatorname{span}_{\mathbb{R}}\{i^{k-j}L_{j,k}, i^{N+k-j}C \cdot L_{j,k}\}_{1 \le j<k \le N} \subseteq \langle \mathcal{A} \rangle_{\text{Lie}}.$$

We claim that the vector space $\mathcal{L}$ is closed under the Lie bracket. Indeed, we already know that $\operatorname{span}_{\mathbb{R}}\{i^{k-j}L_{j,k}\}$ is closed. For the other brackets, we use that from (C18), we have:

$$[i^{k-j}L_{j,k}, i^{N+m-l}C \cdot L_{l,m}] = i^N C \cdot [i^{k-j}L_{j,k}, i^{m-l}L_{l,m}],$$
$$[i^{N+k-j}C \cdot L_{j,k}, i^{N+m-l}C \cdot L_{l,m}] = [i^{k-j}L_{j,k}, i^{m-l}L_{l,m}].$$

As the Lie algebra $\mathcal{L}$ contains $i\mathcal{A}$, it must contain $\langle \mathcal{A} \rangle_{\text{Lie}}$. Therefore, $\mathcal{L} = \langle \mathcal{A} \rangle_{\text{Lie}}$.

Using (C18) again (or from the above brackets), we see that

$$\langle \mathcal{A} \rangle_{\text{Lie}} = \operatorname{span}_{\mathbb{R}}\{(I^{\otimes n} + i^N C) \cdot i^{k-j}L_{j,k}\}_{1 \le j<k \le N} \oplus \operatorname{span}_{\mathbb{R}}\{(I^{\otimes n} - i^N C) \cdot i^{k-j}L_{j,k}\}_{1 \le j<k \le N}$$

is isomorphic as a Lie algebra to a direct sum of two copies of $\operatorname{span}_{\mathbb{R}}\{i^{k-j}L_{j,k}\}_{1 \le j<k \le N} \cong \mathfrak{so}(N)$. Therefore, $\langle \mathcal{A} \rangle_{\text{Lie}} \cong \mathfrak{so}(N) \oplus \mathfrak{so}(N)$. □

Remark C.3. *A circular frustration graph corresponds to free fermionic evolution controlled with one ancilla, where the ancilla degree of freedom can be readily found as the operator C defined in* (C17). *As expected, this is not the only example. Some periodic 1-dimensional spin systems such as TFXY, XY and Kitaev models also have DLAs that are generated from Pauli strings with a circular frustration graph. For those spin models, $C = ZZ\cdots Z$.*

Applying the results of Propositions C.1, C.2, in the following lemmas we determine the Lie algebras $\mathfrak{a}_k(n)$ and $\mathfrak{a}^\circ_k(n)$ for $k = 1, 2, 4, 8, 14$. Examples are presented in Figures S2 and S3.

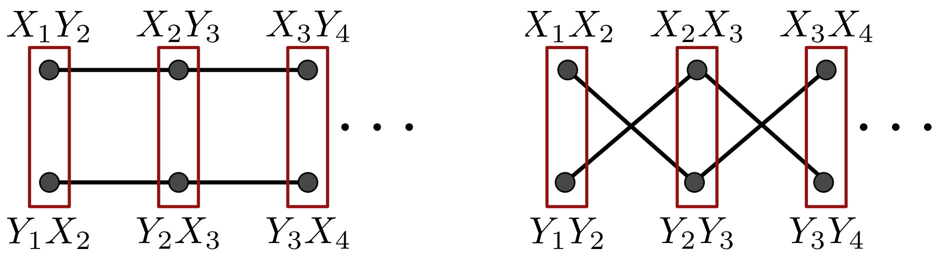

(a) $\mathfrak{a}_2(n)$ (b) $\mathfrak{a}_4(n)$

Figure S2. **Frustration graphs for $\mathfrak{a}_2(n)$ and $\mathfrak{a}_4(n)$.** The red boxes enclose the generators acting on each site $(i,\ i+1)$. Both frustrations graphs are given by two disjoint lines for any n; hence we can conclude that $\mathfrak{a}_2(n) \cong \mathfrak{a}_4(n)$ (cf. Lemma C.3).

Lemma C.6. *We have:*

$$\begin{aligned} \mathfrak{a}_1(n) &\cong \mathfrak{so}(n), \\ \mathfrak{a}_2(n) &\cong \mathfrak{a}_4(n) \cong \mathfrak{so}(n) \oplus \mathfrak{so}(n), \\ \mathfrak{a}_8(n) &\cong \mathfrak{so}(2n-1), \\ \mathfrak{a}_{14}(n) &\cong \mathfrak{so}(2n). \end{aligned}$$

Proof. The proof is based on the frustration graphs of the generating sets of these Lie algebras (see Sect. B I, B IV).

Generators of $\mathfrak{a}_1(n)$ are XY on each adjacent pair of qubits:

$$X_1Y_2, X_2Y_3, X_3Y_4, \ldots, X_{n-1}Y_n.$$

These form a linear frustration graph with $n-1$ vertices, leading to $\mathfrak{a}_1(n) \cong \mathfrak{so}(n)$ (see Figure S3(a)).

The Lie algebra $\mathfrak{a}_2(n)$ is generated by XY and YX on adjacent pairs of qubits:

$$X_1Y_2, X_2Y_3, X_3Y_4, \ldots, X_{n-1}Y_n \qquad \text{and} \qquad Y_1X_2, Y_2X_3, Y_3X_4, \ldots, Y_{n-1}X_n.$$

Both of these form linear frustration graphs with $n-1$ vertices, and commute with each other (see Figure S2(a)). Thus, $\mathfrak{a}_2(n) \cong \mathfrak{so}(n) \oplus \mathfrak{so}(n)$. Note that $\mathfrak{a}_4(n) \cong \mathfrak{a}_2(n)$ due to Lemma C.3 (see also Figure S2).

Since $\mathfrak{a}_8 = \mathrm{span}\{XX, XZ, IY\} = \langle XX, IY\rangle_{\mathrm{Lie}}$, we can generate $\mathfrak{a}_8(n)$ by:

$$X_1X_2, Y_2, X_2X_3, Y_3, X_3X_4, Y_4, \ldots, X_{n-1}X_n, Y_n. \tag{C19}$$

These form a linear frustration graph with $2(n-1)$ vertices; hence $\mathfrak{a}_8(n) \cong \mathfrak{so}(2n-1)$ (see Figure S3(b)).

Similarly, note that

$$\mathfrak{a}_{14} = \mathrm{span}\{XX, YY, XY, YX, ZI, IZ\} = \langle XX, ZI, IZ\rangle_{\mathrm{Lie}},$$

because $[XX, ZI] = -2iYX$, $[XX, IZ] = -2iXY$, and $[XY, ZI] = -2iYY$. Thus, $\mathfrak{a}_{14}(n)$ is generated by:

$$Z_1, X_1X_2, Z_2, X_2X_3, Z_3, X_3X_4, Z_4, \ldots, X_{n-1}X_n, Z_n, \tag{C20}$$

which gives a linear frustration graph with $2n-1$ vertices. Hence $\mathfrak{a}_{14}(n) \cong \mathfrak{so}(2n)$ (see Figure S3(c)). □

Lemma C.7. *We have $\mathfrak{a}^\circ_k(n) \cong \mathfrak{a}_k(n)^{\oplus 2}$ for $k = 1, 2, 14$ and $n \geq 3$.*

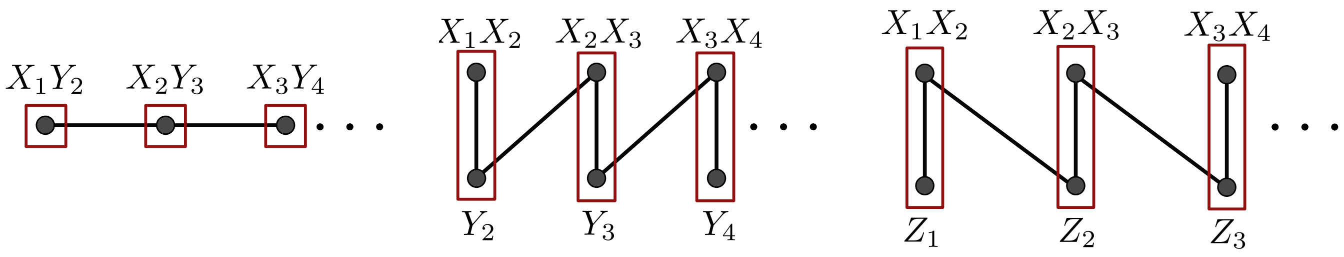

(a) $\mathfrak{a}_1(n)$ (b) $\mathfrak{a}_8(n)$ (c) $\mathfrak{a}_{14}(n)$

Figure S3. **Visualization of the frustration graphs of generators of certain Lie algebras.** The red boxes enclose the generators acting on each site $(i, i+1)$. For the three cases $\mathfrak{a}_1(n)$, $\mathfrak{a}_8(n)$ and $\mathfrak{a}_{14}(n)$, we see that the frustration graph is a line for any n. For (a), the 2-site DLA $\mathfrak{a}_1(2) \cong \mathfrak{u}(1)$, but as n grows we find $\mathfrak{a}_1(n) \cong \mathfrak{so}(n)$. (b) The frustration graph is a line with $2n-2$ vertices; hence $\mathfrak{a}_8(n) \cong \mathfrak{so}(2n-1)$. (c) The frustration graph is a line with $2n-1$ vertices, giving $\mathfrak{a}_{14}(n) \cong \mathfrak{so}(2n)$.

Proof. The Lie algebra $\mathfrak{a}_1^\circ(n)$ is generated by XY applied on adjacent qubits, including periodic boundary conditions:

$$X_1Y_2, X_2Y_3, X_3Y_4, \ldots, X_{n-1}Y_n, X_nY_1.$$

The frustration graph is a circle with n vertices. Therefore, $\mathfrak{a}_1^\circ(n) \cong \mathfrak{so}(n)^{\oplus 2} \cong \mathfrak{a}_1(n)^{\oplus 2}$.

The Lie algebra $\mathfrak{a}_2^\circ(n)$ is generated by XY and YX applied on adjacent qubits with periodic boundary conditions:

$$X_1Y_2, X_2Y_3, X_3Y_4, \ldots, X_{n-1}Y_n, X_nY_1 \qquad \text{and} \qquad Y_1X_2, Y_2X_3, Y_3X_4, \ldots, Y_{n-1}X_n, Y_nX_1.$$

These form two circular frustration graphs with n vertices that are disconnected from each other. Thus, $\mathfrak{a}_2^\circ(n) \cong \mathfrak{so}(n)^{\oplus 2} \oplus \mathfrak{so}(n)^{\oplus 2} \cong \mathfrak{a}_2(n)^{\oplus 2}$.

Using the generating set (C20) of $\mathfrak{a}_{14}(n)$, we see that $\mathfrak{a}_{14}^\circ(n)$ can be generated by:

$$Z_1, X_1X_2, Z_2, X_2X_3, Z_3, X_3X_4, Z_4, \ldots, X_{n-1}X_n, Z_n, X_nX_1.$$

This leads to a circular frustration graph with $2n$ vertices, so $\mathfrak{a}_{14}^\circ(n) \cong \mathfrak{so}(2n)^{\oplus 2} \cong \mathfrak{a}_{14}(n)^{\oplus 2}$. □

Lemma C.8. *We have* $\mathfrak{a}_8^\circ(n) \cong \mathfrak{a}_{14}^\circ(n) \cong \mathfrak{so}(2n)^{\oplus 2}$ *for all* $n \geq 3$.

Proof. The generating sets (C19) and (C20) of $\mathfrak{a}_8(n)$ and $\mathfrak{a}_{14}(n)$ are the same after swapping $Y \rightleftharpoons Z$, except that $\mathfrak{a}_8(n)$ does not have Y_1. When the periodic boundary condition is applied, this difference disappears and we obtain that $\mathfrak{a}_8^\circ(n) \cong \mathfrak{a}_{14}^\circ(n)$. □

Lemma C.9. *We have* $\mathfrak{a}_4^\circ(n) \cong \begin{cases} \mathfrak{so}(2n)^{\oplus 2}, & n \text{ odd}, \\ \mathfrak{so}(n)^{\oplus 4}, & n \text{ even}. \end{cases}$

Proof. As $\mathfrak{a}_4 = \langle XX, YY \rangle_{\text{Lie}}$, the generators of $\mathfrak{a}_4^\circ(n)$ are:

$$X_1X_2, Y_1Y_2, X_2X_3, Y_2Y_3, \ldots, X_{n-1}X_n, Y_{n-1}Y_n, X_nX_1, Y_nY_1.$$

For odd n, these generators form a circular frustration graph with $2n$ vertices:

$$X_1X_2, Y_2Y_3, X_3X_4, Y_4Y_5, \ldots, Y_{n-1}Y_n, X_nX_1, Y_1Y_2, X_2X_3, Y_3Y_4, X_4X_5, \ldots, X_{n-1}X_n, Y_nY_1.$$

Hence, in this case, $\mathfrak{a}_4^\circ(n) \cong \mathfrak{so}(2n)^{\oplus 2}$.

When n is even, the generators form two disjoint circles with n vertices each:

$$X_1X_2, Y_2Y_3, X_3X_4, \ldots, X_{n-1}X_n, Y_nY_1 \qquad \text{and} \qquad Y_1Y_2, X_2X_3, Y_3Y_4, \ldots, Y_{n-1}Y_n, X_nX_1.$$

In this case, we get $\mathfrak{a}_4^\circ(n) \cong \mathfrak{so}(n)^{\oplus 2} \oplus \mathfrak{so}(n)^{\oplus 2}$. □

Remark C.4. *Notice that, although* $\mathfrak{a}_2(n) \cong \mathfrak{a}_4(n)$ *for all* $n \geq 3$*, we have* $\mathfrak{a}_2^\circ(n) \ncong \mathfrak{a}_4^\circ(n)$ *for odd* n.

IV. Stabilizers, commutants, and centralizers

For any Pauli string $A \in \mathcal{P}_k$, we will use the notation $P_A = AAA\cdots \in \mathcal{P}_n$ truncated to the n-th qubit. For example,

$$P_X = XXX\cdots\,, \qquad P_{YZ} = YZYZ\cdots\,, \qquad P_{ZY} = ZYZY\cdots\,,$$
$$P_{XYZ} = XYZXYZ\cdots\,, \qquad P_{YZX} = YZXYZX\cdots\,, \qquad P_{ZXY} = ZXYZXY\cdots\,,$$

where these are viewed as elements of $\mathcal{P}_n$. In particular, $P_I = I\cdots I = I^{\otimes n}$. Recall that $\pm\mathcal{P}_n \cup \pm i\mathcal{P}_n$ is a group under the matrix product, the Pauli group (see Sect. A I).

For any set of matrices $\mathcal{A} \subseteq \mathbb{C}^{2^n\times 2^n}$, we define its *stabilizer* $\mathrm{St}(\mathcal{A}) \subseteq \mathcal{P}_n$ as the set of all Pauli strings that commute with every element of $\mathcal{A}$. It is clear that $\mathrm{St}(\mathcal{A})$ is closed under multiplication, so after allowing appropriate powers of i it is a group. There are two related notions called the commutant and centralizer. All of these consist of elements commuting with the given set $\mathcal{A}$ but differ in where such elements are and what structure they form: the stabilizer is a subgroup of the Pauli group (up to factors of $\pm 1, \pm i$); the commutant is a subalgebra of the associative algebra $\mathbb{C}^{2^n\times 2^n}$ of all complex matrices; while the centralizer is a subalgebra of the real Lie algebra $\mathfrak{su}(2^n)$.

Remark C.5. *The* commutant *of a set $\mathcal{A} \subseteq \mathbb{C}^{2^n\times 2^n}$ is defined as the set $\mathcal{A}'$ of all $2^n \times 2^n$ complex matrices that commute with all elements of $\mathcal{A}$. Then $\mathcal{A}'$ is closed under addition, multiplication, and multiplication by any complex scalar, i.e., it is an associative algebra over $\mathbb{C}$. Note that $\mathcal{A} \subseteq \mathcal{A}'' := (\mathcal{A}')'$. By (a finite-dimensional version of) von Neumann's Double Commutant Theorem (see e.g. [15], Theorem 6.2.5), $\mathcal{A}''$ is the associative algebra generated by $\mathcal{A}$. In particular, for a complex vector space $\mathcal{A}$, we have $\mathcal{A}'' = \mathcal{A} + \mathbb{C}I^{\otimes n}$ if and only if $\mathcal{A}$ is closed under multiplication.*

Remark C.6. *The* centralizer *of a set $\mathcal{A} \subseteq \mathfrak{su}(2^n)$ is the set $\mathfrak{su}(2^n)^{\mathcal{A}} \subseteq \mathfrak{su}(2^n)$ of all traceless skew-Hermitian $2^n \times 2^n$ matrices that commute with all elements of $\mathcal{A}$. Then $\mathfrak{su}(2^n)^{\mathcal{A}}$ is closed under addition, commutator, and multiplication by any real scalar, i.e., it is a Lie subalgebra of $\mathfrak{su}(2^n)$.*

In order to explain the precise relation of the stabilizer to the commutant and centralizer, we first formulate a lemma, which will also be useful later.

Lemma C.10. *Suppose that $[B, C] = 0$ for some $B \in \mathcal{P}_n$ and*

$$C = \sum \alpha_j C_j, \qquad \alpha_j \in \mathbb{R},\;\; \alpha_j \neq 0,\;\; C_j \in \mathcal{P}_n,$$

where the C_j are distinct. Then $[B, C_j] = 0$ for all j.

Proof. Assume, to the contrary, that $[B, C_j] \neq 0$ for some index j. Recall that $[B, [B, C_j]] = 4C_j$ whenever $[B, C_j] \neq 0$, by Corollary A.1. Hence,

$$0 = [B, [B, C]] = \sum \alpha_j [B, [B, C_j]] = 4 \sum{}' \alpha_j C_j,$$

where $\sum'$ denotes the sum over all indices j such that $[B, C_j] \neq 0$. This contradicts the fact that all $\alpha_j \neq 0$, as the vectors C_j are linearly independent. □

As a consequence of Lemma C.10, we obtain:

Corollary C.1. *Suppose that $\mathcal{A} \subseteq \mathcal{P}_n$ is a set of Pauli strings. Then its commutant $\mathcal{A}' = \mathrm{span}_{\mathbb{C}}\mathrm{St}(\mathcal{A})$ is the complex linear span of the stabilizer $\mathrm{St}(\mathcal{A})$, while the centralizer $\mathfrak{su}(2^n)^{\mathcal{A}} = \mathrm{span}(\mathrm{St}(\mathcal{A}) \setminus \{I^{\otimes n}\})$ is the real span of $i(\mathrm{St}(\mathcal{A}) \setminus \{I^{\otimes n}\})$.*

When $\mathfrak{s} \subseteq \mathfrak{su}(2^n)$ is a Lie subalgebra, a given Pauli string commutes with all elements of $\mathfrak{s}$ if and only if it commutes with all generators of $\mathfrak{s}$. In other words, we have

$$\mathrm{St}(\langle\mathcal{A}\rangle_{\mathrm{Lie}}) = \mathrm{St}(\mathcal{A}). \tag{C21}$$

Thus, to determine the stabilizers of our Lie subalgebras of $\mathfrak{su}(2^n)$, it suffices to find the stabilizers of their generating sets. In the case $n = 2$, it is easy to find the answer by inspection, which is given as follows:

$$\mathrm{St}(\mathfrak{a}_0) = \{II, IX, XI, XX, YY, YZ, ZY, ZZ\},$$
$$\mathrm{St}(\mathfrak{a}_1) = \{II, IY, XI, XY, YX, YZ, ZX, ZZ\},$$

$$
\begin{aligned}
\mathrm{St}(\mathfrak{a}_2) &= \{II, XY, YX, ZZ\},\\
\mathrm{St}(\mathfrak{a}_3) &= \mathrm{St}(\mathfrak{a}_6) = \{II, XX, YZ, ZY\},\\
\mathrm{St}(\mathfrak{a}_4) &= \mathrm{St}(\mathfrak{a}_7) = \{II, XX, YY, ZZ\},\\
\mathrm{St}(\mathfrak{a}_5) &= \mathrm{St}(\mathfrak{a}_{10}) = \{II, XY, YZ, ZX\},\\
\mathrm{St}(\mathfrak{a}_8) &= \{II, XI, YY, ZY\},\\
\mathrm{St}(\mathfrak{a}_9) &= \{II, XI, YX, ZX\},\\
\mathrm{St}(\mathfrak{a}_{11}) &= \{II, XY\},\\
\mathrm{St}(\mathfrak{a}_{12}) &= \mathrm{St}(\mathfrak{a}_{17}) = \mathrm{St}(\mathfrak{a}_{19}) = \{II, YZ\},\\
\mathrm{St}(\mathfrak{a}_{13}) &= \mathrm{St}(\mathfrak{a}_{20}) = \{II, XX\},\\
\mathrm{St}(\mathfrak{a}_{14}) &= \{II, ZZ\},\\
\mathrm{St}(\mathfrak{a}_{15}) &= \mathrm{St}(\mathfrak{b}_2) = \mathrm{St}(\mathfrak{b}_4) = \{II, XI\},\\
\mathrm{St}(\mathfrak{b}_0) &= \mathrm{St}(\mathfrak{b}_1) = \{II, XX, XI, IX\},\\
\mathrm{St}(\mathfrak{a}_k) &= \mathrm{St}(\mathfrak{b}_3) = \{II\}, \qquad k = 16, 18, 21, 22.
\end{aligned}
$$

Using that, we can find the stabilizers of the subalgebras of $\mathfrak{su}(2^n)$.

Proposition C.3. *For $n \geq 3$, we have the following stabilizers:*

$$
\begin{aligned}
\mathrm{St}(\mathfrak{a}_0(n)) &= \{I, X\}^{\otimes n} \cup \{Y, Z\}^{\otimes n},\\
\mathrm{St}(\mathfrak{a}_2(n)) &= \{P_I, P_{XY}, P_{YX}, P_Z\},\\
\mathrm{St}(\mathfrak{a}_3(n)) &= \mathrm{St}(\mathfrak{a}_6(n)) = \{P_I, P_X, P_{YZ}, P_{ZY}\},\\
\mathrm{St}(\mathfrak{a}_4(n)) &= \mathrm{St}(\mathfrak{a}_7(n)) = \{P_I, P_X, P_Y, P_Z\},\\
\mathrm{St}(\mathfrak{a}_5(n)) &= \mathrm{St}(\mathfrak{a}_{10}(n)) = \{P_I, P_{XYZ}, P_{YZX}, P_{ZXY}\},\\
\mathrm{St}(\mathfrak{a}_8(n)) &= \{P_I, P_Y, X_1, ZY\cdots Y\},\\
\mathrm{St}(\mathfrak{a}_9(n)) &= \{P_I, X_1, Y_1X_2, Z_1X_2\},\\
\mathrm{St}(\mathfrak{a}_{13}(n)) &= \mathrm{St}(\mathfrak{a}_{20}(n)) = \{P_I, P_X\},\\
\mathrm{St}(\mathfrak{a}_{14}(n)) &= \{P_I, P_Z\},\\
\mathrm{St}(\mathfrak{a}_{15}(n)) &= \mathrm{St}(\mathfrak{b}_2(n)) = \mathrm{St}(\mathfrak{b}_4(n)) = \{P_I, X_1\},\\
\mathrm{St}(\mathfrak{b}_0(n)) &= \mathrm{St}(\mathfrak{b}_1(n)) = \{I, X\}^{\otimes n},\\
\mathrm{St}(\mathfrak{a}_k(n)) &= \mathrm{St}(\mathfrak{b}_3(n)) = \{P_I\}, \qquad k = 11, 12, 16\text{–}19, 21, 22.
\end{aligned}
$$

Proof. Recall that, for any subalgebra $\mathfrak{a} \subseteq \mathfrak{su}(4)$ generated by Pauli strings, the subalgebra $\mathfrak{a}(n) \subseteq \mathfrak{su}(2^n)$ is generated by all Pauli strings A_iB_{i+1}, where $AB \in \mathfrak{a}$, $1 \leq i \leq n-1$ (see (B5)). Thus, a Pauli string $P^1 \otimes \cdots \otimes P^n \in \mathcal{P}_n$ is in $\mathrm{St}(\mathfrak{a}(n))$ if and only if $P^i \otimes P^{i+1} \in \mathrm{St}(\mathfrak{a})$ for all $1 \leq i \leq n-1$. Using this observation and the knowledge of all $\mathrm{St}(\mathfrak{a})$, it is straightforward to determine $\mathrm{St}(\mathfrak{a}(n))$.

Let us consider the case of $\mathfrak{a}_2$ as an illustration. Since $\mathrm{St}(\mathfrak{a}_2) = \{II, XY, YX, ZZ\}$, we want to find all Pauli strings, such that for any two consecutive qubits, we have either II, XY, YX, or ZZ. The only possible such strings are $I\cdots I$, $XYXY\cdots$, $YXYX\cdots$, or $Z\cdots Z$. □

The answer for $\mathrm{St}(\mathfrak{a}_1(n))$ is given without proof in Remark C.7 below, as it is more complicated but not needed for the rest of the paper. For future use, we will need the centers of some of the above stabilizers. Let us recall that the *center* $Z(G)$ of a group G consists of all $z \in G$ that commute with every $g \in G$. In particular, a group G is Abelian if and only if $Z(G) = G$.

Lemma C.11. *For $n \geq 3$, we have the following centers:*

$$
Z\big(\mathrm{St}(\mathfrak{a}_2(n))\big) = \begin{cases} \{P_I, P_{XY}, P_{YX}, P_Z\}, & n \text{ even},\\ \{P_I\}, & n \text{ odd}, \end{cases}
$$

$$
Z\big(\mathrm{St}(\mathfrak{a}_3(n))\big) = Z\big(\mathrm{St}(\mathfrak{a}_6(n))\big) = \begin{cases} \{P_I, P_X, P_{YZ}, P_{ZY}\}, & n \text{ even},\\ \{P_I\}, & n \text{ odd}, \end{cases}
$$

$$
Z\big(\mathrm{St}(\mathfrak{a}_4(n))\big) = Z\big(\mathrm{St}(\mathfrak{a}_7(n))\big) = \begin{cases} \{P_I, P_X, P_Y, P_Z\}, & n \text{ even},\\ \{P_I\}, & n \text{ odd}, \end{cases}
$$

$$\begin{aligned}
Z\big(\mathrm{St}(\mathfrak{a}_5(n))\big) = Z\big(\mathrm{St}(\mathfrak{a}_{10}(n))\big) &= \begin{cases} \{P_I, P_{XYZ}, P_{YZX}, P_{ZXY}\}, & n \text{ even}, \\ \{P_I\}, & n \text{ odd}, \end{cases} \\
Z\big(\mathrm{St}(\mathfrak{a}_{13}(n))\big) = Z\big(\mathrm{St}(\mathfrak{a}_{20}(n))\big) &= \{P_I, P_X\}, \\
Z\big(\mathrm{St}(\mathfrak{a}_{14}(n))\big) &= \{P_I, P_Z\}, \\
Z\big(\mathrm{St}(\mathfrak{a}_{15}(n))\big) &= \{P_I, X_1\}, \\
Z\big(\mathrm{St}(\mathfrak{a}_k(n))\big) &= \{P_I\}, \qquad k = 0, 8, 9, 11, 12, 16{-}19, 21, 22.
\end{aligned}$$

Next, we determine the stabilizers in the periodic case.

Proposition C.4. *For $n \geq 3$, we have the following stabilizers:*

$$\begin{aligned}
\mathrm{St}(\mathfrak{a}_0^\circ(n)) &= \{I, X\}^{\otimes n} \cup \{Y, Z\}^{\otimes n}, \\
\mathrm{St}(\mathfrak{a}_2^\circ(n)) &= \begin{cases} \{P_I, P_{XY}, P_{YX}, P_Z\}, & n \text{ even}, \\ \{P_I, P_Z\}, & n \text{ odd}, \end{cases} \\
\mathrm{St}(\mathfrak{a}_3^\circ(n)) = \mathrm{St}(\mathfrak{a}_6^\circ(n)) &= \begin{cases} \{P_I, P_X, P_{YZ}, P_{ZY}\}, & n \text{ even}, \\ \{P_I, P_X\}, & n \text{ odd}, \end{cases} \\
\mathrm{St}(\mathfrak{a}_4^\circ(n)) = \mathrm{St}(\mathfrak{a}_7^\circ(n)) &= \{P_I, P_X, P_Y, P_Z\}, \\
\mathrm{St}(\mathfrak{a}_5^\circ(n)) = \mathrm{St}(\mathfrak{a}_{10}^\circ(n)) &= \begin{cases} \{P_I, P_{XYZ}, P_{YZX}, P_{ZXY}\}, & n \equiv 0 \mod 3, \\ \{P_I\}, & n \equiv \pm 1 \mod 3, \end{cases} \\
\mathrm{St}(\mathfrak{a}_8^\circ(n)) &= \{P_I, P_Y\}, \\
\mathrm{St}(\mathfrak{a}_{13}^\circ(n)) = \mathrm{St}(\mathfrak{a}_{20}^\circ(n)) &= \{P_I, P_X\}, \\
\mathrm{St}(\mathfrak{a}_{14}^\circ(n)) &= \{P_I, P_Z\}, \\
\mathrm{St}(\mathfrak{b}_0^\circ(n)) = \mathrm{St}(\mathfrak{b}_1^\circ(n)) &= \{I, X\}^{\otimes n}, \\
\mathrm{St}(\mathfrak{a}_k^\circ(n)) = \mathrm{St}(\mathfrak{b}_l^\circ(n)) &= \{P_I\}, \qquad k = 9, 11, 12, 15{-}19, 21, 22, \quad l = 2, 3, 4.
\end{aligned}$$

Proof. For any subalgebra $\mathfrak{a} \subseteq \mathfrak{su}(4)$ generated by Pauli strings, comparing the definitions of $\mathfrak{a}(n)$ and $\mathfrak{a}^\circ(n)$ (see (B5), (B6)), we see that $\mathrm{St}(\mathfrak{a}^\circ(n))$ consists of all Pauli strings $P^1 \otimes \cdots \otimes P^n \in \mathrm{St}(\mathfrak{a}(n))$ such that $P^n \otimes P^1 \in \mathrm{St}(\mathfrak{a})$. Thus, we determine $\mathrm{St}(\mathfrak{a}^\circ(n))$ by inspecting all elements of $\mathrm{St}(\mathfrak{a}(n))$. □

Remark C.7. *One can show that $\mathrm{St}(\mathfrak{a}_1(n)) = \mathrm{St}(\mathfrak{a}_1^\circ(n))$, and as a group it is generated by the elements P_Z, Y_1X_2, Y_2X_3, …, $Y_{n-1}X_n$. This means that $\mathrm{St}(\mathfrak{a}_1(n))$ consists of all possible matrix products of these generators. For the center, we have $Z\big(\mathrm{St}(\mathfrak{a}_1(n))\big) = \{P_I, P_Z\}$.*

We also find the stabilizers in the permutation-invariant case.

Proposition C.5. *For $n \geq 3$, we have the following stabilizers:*

$$\begin{aligned}
\mathrm{St}(\mathfrak{a}_0^\pi(n)) &= \{I, X\}^{\otimes n} \cup \{Y, Z\}^{\otimes n}, \\
\mathrm{St}(\mathfrak{a}_2^\pi(n)) = \mathrm{St}(\mathfrak{a}_{14}^\pi(n)) &= \{P_I, P_Z\}, \\
\mathrm{St}(\mathfrak{a}_4^\pi(n)) = \mathrm{St}(\mathfrak{a}_7^\pi(n)) &= \{P_I, P_X, P_Y, P_Z\}, \\
\mathrm{St}(\mathfrak{a}_6^\pi(n)) = \mathrm{St}(\mathfrak{a}_{20}^\pi(n)) &= \{P_I, P_X\}, \\
\mathrm{St}(\mathfrak{b}_0^\pi(n)) = \mathrm{St}(\mathfrak{b}_1^\pi(n)) &= \{I, X\}^{\otimes n}, \\
\mathrm{St}(\mathfrak{a}_{16}^\pi(n)) = \mathrm{St}(\mathfrak{b}_3^\pi(n)) &= \{P_I\}.
\end{aligned}$$

Proof. For any subalgebra $\mathfrak{a} \subseteq \mathfrak{su}(4)$ generated by Pauli strings, from the definition of $\mathfrak{a}^\pi(n)$ (see (B10)), we see that $\mathrm{St}(\mathfrak{a}^\pi(n))$ consists of all Pauli strings $P^1 \otimes \cdots \otimes P^n \in \mathcal{P}_n$ such that $P^i \otimes P^j \in \mathrm{St}(\mathfrak{a})$ for all $i \neq j$. Moreover, as we explained in Sect. B IV, $\mathfrak{a}$ can be assumed itself invariant under the flip of the two qubits; so we only need to consider $\mathfrak{a}_k^\pi(n)$ for $k = 0, 2, 4, 6, 7, 14, 16, 20$ and $\mathfrak{b}_l^\pi(n)$ for $l = 0, 1, 3$. □

We finish this subsection with an important lemma.

Lemma C.12. *The Lie algebras $\mathfrak{a}_k(n)$ have trivial centers for $1 \leq k \leq 22$ and $n \geq 3$.*

Proof. Due to Lemma A.2, $\mathfrak{a}_k(n)$ has a basis $\mathcal{B} \subseteq i\mathcal{P}_n \cap \mathfrak{a}_k(n)$ consisting of Pauli strings times i. Suppose that $\mathfrak{a}_k(n)$ has a central element $c \neq 0$, and write c as a linear combination of basis vectors:

$$c = \sum \alpha_j c_j, \qquad \alpha_j \in \mathbb{R}, \;\; \alpha_j \neq 0, \;\; c_j \in \mathcal{B}.$$

We claim that all c_j in this expression are central too. Indeed, by Lemma C.10, if $[b, c] = 0$ for a basis vector $b \in \mathcal{B}$, then $[b, c_j] = 0$ for all j.

Therefore, without loss of generality, we can take $c \in \mathcal{B}$ to be itself one of the basis vectors. One can verify by inspection that the generators of $\mathfrak{a}_k(n)$ are not central for $1 \leq k \leq 22$ and $n \geq 3$; for example, by checking that $Z\big(\mathrm{St}(\mathfrak{a}_k(n))\big)$ does not contain any of the generators of $\mathfrak{a}_k(n)$. Thus, we can write c in the form

$$c = \alpha \, \mathrm{ad}_{a_1} \mathrm{ad}_{a_2} \cdots \mathrm{ad}_{a_r}(a_{r+1}),$$

for some $r \geq 1$, $\alpha \in \mathbb{R}$, $\alpha \neq 0$, and generators $a_1, \dots, a_{r+1}$. Since all generators $a_j \in i\mathcal{P}_n$, we have $a := \mathrm{ad}_{a_2} \cdots \mathrm{ad}_{a_r}(a_{r+1}) \in i\mathbb{R}\mathcal{P}_n$. Then $c = \alpha[a_1, a] \neq 0$, and from Corollary A.1, we get $-4a = [a_1, [a_1, a]] = \alpha^{-1}[a_1, c] = 0$, which implies that $c = 0$, a contradiction. □

V. Upper bounds for $\mathfrak{a}_k(n)$

In this subsection, we establish upper bounds for the Lie algebras $\mathfrak{a}_k(n)$, i.e., we find certain subalgebras $\mathfrak{g}_k(n)^{\theta_k} \subseteq \mathfrak{su}(2^n)$ that contain $\mathfrak{a}_k(n)$. Then, in the next subsection C VI, we will prove that these bounds are exact, that is $\mathfrak{a}_k(n) = \mathfrak{g}_k(n)^{\theta_k}$. While $\mathfrak{a}_k(n)$ is defined in terms of its generators, $\mathfrak{g}_k(n)^{\theta_k}$ is defined as the set of elements of $\mathfrak{su}(2^n)$ that are fixed under certain automorphisms and involutions. This will allow us, in the following subsection C VII, to identify the Lie algebras $\mathfrak{a}_k(n)$ as direct sums of $\mathfrak{su}$, $\mathfrak{so}$, and $\mathfrak{sp}$ Lie algebras.

We start by recalling that any Pauli string $P \in \mathcal{P}_n$ defines an automorphism of $\mathfrak{su}(2^n)$ by conjugation $a \mapsto PaP$ (recall that $P = P^\dagger = P^{-1}$); see Lemma A.4. We will denote by $\mathfrak{su}(2^n)^P$ the set of *fixed points* under this automorphism, i.e., the set of all $a \in \mathfrak{su}(2^n)$ such that $PaP = a$. The latter is equivalent to $Pa = aP$; hence $\mathfrak{su}(2^n)^P$ is equal to the *centralizer* of P, i.e., the set of all $a \in \mathfrak{su}(2^n)$ that commute with P (see Remark C.6). More generally, for a set Φ of automorphisms of a Lie algebra $\mathfrak{g}$, we will denote by $\mathfrak{g}^\Phi$ the set of fixed points $a \in \mathfrak{g}$ such that $\phi(a) = a$ for all $\phi \in \Phi$.

Given a subalgebra $\mathfrak{s} \subseteq \mathfrak{su}(2^n)$, recall from Sect. C IV that its stabilizer $\mathrm{St}(\mathfrak{s})$ consists of all Pauli strings $P \in \mathcal{P}_n$ such that $[a, P] = 0$ for every $a \in \mathfrak{s}$. On the other hand, the centralizer $\mathfrak{su}(2^n)^{\mathrm{St}(\mathfrak{s})}$ of $\mathrm{St}(\mathfrak{s})$ in $\mathfrak{su}(2^n)$ consists of all $a \in \mathfrak{su}(2^n)$ such that $[a, P] = 0$ for every $P \in \mathrm{St}(\mathfrak{s})$. Hence, by definition,

$$\mathfrak{s} \subseteq \mathfrak{su}(2^n)^{\mathrm{St}(\mathfrak{s})}. \tag{C22}$$

This simple observation will be the key to finding upper bounds for our subalgebras $\mathfrak{a}_k(n)$, because we have already determined their stabilizers in Proposition C.3. Here is another observation, which in some cases will allow us to further reduce the upper bound.

Lemma C.13. *For any subalgebra $\mathfrak{s} \subseteq \mathfrak{su}(2^n)$, we have*

$$\mathrm{St}(\mathfrak{s}) \cap \mathfrak{su}(2^n)^{\mathrm{St}(\mathfrak{s})} = Z\big(\mathrm{St}(\mathfrak{s})\big) \setminus \{I^{\otimes n}\} \subseteq Z\big(\mathfrak{su}(2^n)^{\mathrm{St}(\mathfrak{s})}\big),$$

where $Z(G)$ denotes the center of a group or an algebra G.

Proof. Elements $z \in \mathrm{St}(\mathfrak{s}) \cap \mathfrak{su}(2^n)^{\mathrm{St}(\mathfrak{s})}$ satisfy $[z, a] = 0$ for every $a \in \mathfrak{su}(2^n)^{\mathrm{St}(\mathfrak{s})}$ since $z \in \mathrm{St}(\mathfrak{s})$, and $[z, P] = 0$ for every $P \in \mathrm{St}(\mathfrak{s})$ since $z \in \mathfrak{su}(2^n)^{\mathrm{St}(\mathfrak{s})}$. Hence, such z are central in both $\mathrm{St}(\mathfrak{s})$ and $\mathfrak{su}(2^n)^{\mathrm{St}(\mathfrak{s})}$. However, $I^{\otimes n}$ is excluded, because $I^{\otimes n} \notin \mathfrak{su}(2^n)$. □

As the Lie algebras $\mathfrak{s} = \mathfrak{a}_k(n)$ for $1 \leq k \leq 22$, $n \geq 3$ have trivial centers (Lemma C.12), for them we can reduce the upper bound $\mathfrak{su}(2^n)^{\mathrm{St}(\mathfrak{s})}$ if we quotient by the central elements $Z\big(\mathrm{St}(\mathfrak{s})\big) \setminus \{I^{\otimes n}\}$. Thus, we introduce the notation

$$\mathfrak{g}_k(n) := \mathfrak{su}(2^n)^{\mathrm{St}(\mathfrak{a}_k(n))} / \mathrm{span}\big(Z\big(\mathrm{St}(\mathfrak{a}_k(n))\big) \setminus \{I^{\otimes n}\}\big), \tag{C23}$$

and from the above discussion, we have

$$\mathfrak{a}_k(n) \subseteq \mathfrak{g}_k(n), \qquad 1 \leq k \leq 22, \quad n \geq 3. \tag{C24}$$

Using Proposition C.3 and Lemma C.11, we can write explicitly:

$$\mathfrak{g}_3(n) = \mathfrak{g}_6(n) = \begin{cases} \mathfrak{su}(2^n)^{\{P_X, P_{YZ}, P_{ZY}\}}/\operatorname{span}\{P_X, P_{YZ}, P_{ZY}\}, & n \text{ even}, \\ \mathfrak{su}(2^n)^{\{P_X, P_{YZ}, P_{ZY}\}}, & n \text{ odd}, \end{cases} \tag{C25}$$

$$\mathfrak{g}_5(n) = \mathfrak{g}_{10}(n) = \begin{cases} \mathfrak{su}(2^n)^{\{P_{XYZ}, P_{YZX}, P_{ZXY}\}}/\operatorname{span}\{P_{XYZ}, P_{YZX}, P_{ZXY}\}, & n \text{ even}, \\ \mathfrak{su}(2^n)^{\{P_{XYZ}, P_{YZX}, P_{ZXY}\}}, & n \text{ odd}, \end{cases} \tag{C26}$$

$$\mathfrak{g}_7(n) = \begin{cases} \mathfrak{su}(2^n)^{\{P_X, P_Y, P_Z\}}/\operatorname{span}\{P_X, P_Y, P_Z\}, & n \text{ even}, \\ \mathfrak{su}(2^n)^{\{P_X, P_Y, P_Z\}}, & n \text{ odd}, \end{cases} \tag{C27}$$

$$\mathfrak{g}_9(n) = \mathfrak{su}(2^n)^{\{X_1, Y_1X_2, Z_1X_2\}}, \tag{C28}$$

$$\mathfrak{g}_{11}(n) = \mathfrak{g}_{16}(n) = \mathfrak{su}(2^n), \tag{C29}$$

$$\mathfrak{g}_{13}(n) = \mathfrak{g}_{20}(n) = \mathfrak{su}(2^n)^{P_X}/\operatorname{span}\{P_X\}, \tag{C30}$$

$$\mathfrak{g}_{15}(n) = \mathfrak{su}(2^n)^{X_1}/\operatorname{span}\{X_1\}. \tag{C31}$$

It turns out that in some cases the inclusions (C24) are strict, and we need to reduce the Lie algebras $\mathfrak{g}_k(n)$ further to smaller subalgebras. We do that by finding suitable involutions and then taking their fixed points (see Sect. A III).

Theorem C.1. *We have*

$$\mathfrak{a}_k(n) = \mathfrak{g}_k(n), \qquad k = 6, 7, 10, 13, 15, 20, \quad n \geq 3. \tag{C32}$$

In the remaining cases, there exists an involution θ_k of $\mathfrak{g}_k(n)$, such that

$$\mathfrak{a}_k(n) = \mathfrak{g}_k(n)^{\theta_k}, \qquad k = 3, 5, 9, 11, 16, \quad n \geq 3, \tag{C33}$$

is the set of fixed points under θ_k.

In the remainder of this subsection, we will construct the involution θ_k explicitly, and will check that $\mathfrak{a}_k(n) \subseteq \mathfrak{g}_k(n)^{\theta_k}$. The opposite inclusion will be proved in the next subsection. Then, in Sect. C VII, we will identify the Lie algebras $\mathfrak{g}_k(n)^{\theta_k}$ with those from Theorem IV.1. For $k = 9, 11, 16$, the involution θ_k will have the form (cf. Lemma A.4):

$$\theta(g) = -Qg^TQ \tag{C34}$$

for some given Pauli string $Q \in \mathcal{P}_n$.

Lemma C.14. *For any fixed Pauli string $Q \in \mathcal{P}_n$, (C34) defines an involution of $\mathfrak{su}(2^n)$, which restricts to an involution of $\mathfrak{g}_k(n)$ for all k.*

Proof. We already know from Lemma A.4 that θ is an involution of $\mathfrak{su}(2^n)$, so we only need to check that $\theta(g) \in \mathfrak{g}_k(n)$ for all $g \in \mathfrak{g}_k(n)$. As before, write $\mathfrak{s} = \mathfrak{a}_k(n)$ for short. Consider an element $g \in \mathfrak{su}(2^n)^{\operatorname{St}(\mathfrak{s})}$, which means that $[g, P] = 0$ for all $P \in \operatorname{St}(\mathfrak{s})$. Then

$$\theta(P) = -QP^TQ = \pm P,$$

because $P^T = \pm P$ and $PQ = \pm QP$ for any two Pauli strings $P, Q \in \mathcal{P}_n$ (the signs here are not coordinated). Hence

$$[\theta(g), P] = \pm[\theta(g), \theta(P)] = \pm\theta([g, P]) = 0,$$

which implies that $\theta(g) \in \mathfrak{su}(2^n)^{\operatorname{St}(\mathfrak{s})}$. Furthermore,

$$\theta(P) = \pm P \in \operatorname{span}\big(Z\big(\operatorname{St}(\mathfrak{s})\big) \setminus \{I^{\otimes n}\}\big) \quad \text{for all} \quad P \in Z\big(\operatorname{St}(\mathfrak{s})\big) \setminus \{I^{\otimes n}\}.$$

Therefore, $\theta(g) \in \mathfrak{g}_k(n)$ for $g \in \mathfrak{g}_k(n)$. □

Let us record the following consequence of the proof of Lemma C.14, which will be useful later.

Corollary C.2. *Every element of $\mathfrak{g}_k(n)^{\theta_k}$ is a linear combination of Pauli strings that are themselves in $\mathfrak{g}_k(n)^{\theta_k}$, i.e.,*

$$\mathfrak{g}_k(n)^{\theta_k} = \operatorname{span}_{\mathbb{R}}\big(i\mathcal{P}_n \cap \mathfrak{g}_k(n)^{\theta_k}\big).$$

Proof. We saw in the proof of Lemma C.14 that $\theta_k(P) = \pm P$ for any Pauli string $P \in \mathcal{P}_n$. Similarly, for any given $S \in \mathrm{St}(\mathfrak{a}_k(n))$, we have $SPS = \pm P$. Elements g of $\mathfrak{g}_k(n)^{\theta_k}$ are determined by the conditions

$$SgS = g = \theta_k(g) \qquad \text{for all} \quad S \in \mathrm{St}(\mathfrak{a}_k(n)).$$

Writing $g \in \mathfrak{su}(2^n)$ as i times a real linear combination of Pauli strings, we see that g satisfies these conditions if and only if every summand does. □

Now we go back to the construction of the involutions θ_k.

Lemma C.15. *For $k = 9, 11, 16$, we define $\theta_k(g) = -Q_k g^T Q_k$, where the Pauli strings Q_k are given as follows:*

$$Q_9 = IYZ \cdots Z, \tag{C35}$$

$$Q_{11} = Q_{16} = I \cdots I \quad \Rightarrow \quad \theta_{11}(g) = \theta_{16}(g) = -g^T. \tag{C36}$$

Then $\mathfrak{a}_k(n) \subseteq \mathfrak{g}_k(n)^{\theta_k}$.

Proof. We already know that $\mathfrak{a}_k(n) \subseteq \mathfrak{g}_k(n)$, so we only need to check that $\theta_k(g) = g$ for all $g \in \mathfrak{a}_k(n)$. It is enough to check this only for the generators g of $\mathfrak{a}_k(n)$, because $\theta_k([a,b]) = [\theta_k(a), \theta_k(b)]$.

For $k = 9$, we take $g = X_i Y_{i+1}$ or $g = X_i Z_{i+1}$. In the first case, $g^T = -g$ and $gQ_9 = Q_9 g$; while in the second case, $g^T = g$ and $gQ_9 = -Q_9 g$. Hence, in both cases we have $\theta_9(g) = g$.

For $k = 11$, the generators are $g = X_i Y_{i+1}, Y_i X_{i+1}, Y_i Z_{i+1}$; while for $k = 16$, the generators are $g = X_i Y_{i+1}, Y_i X_{i+1}, Y_i Z_{i+1}, Z_i Y_{i+1}$. All of them satisfy $g^T = -g$. □

The remaining cases $k = 3$ and $k = 5$ are a little more complicated. The trick is to first embed $\mathfrak{a}_3(n)$ and $\mathfrak{a}_5(n)$ as subalgebras of $\mathfrak{a}_7(n)$. Recall from Sect. C II that $\mathfrak{a}_3(n) \subseteq \mathfrak{a}_6(n)$ and $\mathfrak{a}_6(n) \cong \mathfrak{a}_7(n)$ under the automorphism φ_n of $\mathfrak{su}(2^n)$ that swaps (up to a sign) $Y \rightleftharpoons Z$ on all even qubits (see (C8), (C9)). Then $\tilde{\mathfrak{a}}_3(n) := \varphi_n \mathfrak{a}_3(n) \subset \mathfrak{a}_7(n)$. Likewise, we have $\mathfrak{a}_5(n) \subseteq \mathfrak{a}_{10}(n)$ and $\mathfrak{a}_{10}(n) \cong \mathfrak{a}_7(n)$ under the automorphism γ_n of $\mathfrak{su}(2^n)$ that applies on the j-th qubit γ^j, where γ is the cycle $X \mapsto Z \mapsto Y \mapsto X$ (see (C11), (C12)). Then $\tilde{\mathfrak{a}}_5(n) := \gamma_n \mathfrak{a}_5(n) \subset \mathfrak{a}_7(n)$.

We consider the involutions

$$\tilde{\theta}_k(g) = -Q_k g^T Q_k, \qquad k = 3, 5, \tag{C37}$$

where

$$Q_3 = P_{ZIYX} = (Z_1 Y_3 X_4)(Z_5 Y_7 X_8)(Z_9 Y_{11} X_{12}) \cdots, \tag{C38}$$

$$Q_5 = P_{IYZ} = (Y_2 Z_3)(Y_5 Z_6)(Y_8 Z_9)(Y_{11} Z_{12}) \cdots. \tag{C39}$$

Then we define

$$\theta_3 := \varphi_n^{-1} \tilde{\theta}_3 \varphi_n, \qquad \theta_5 := \gamma_n^{-1} \tilde{\theta}_5 \gamma_n. \tag{C40}$$

Lemma C.16. *For $k = 3, 5$, and θ_k defined as above, we have $\mathfrak{a}_k(n) \subseteq \mathfrak{g}_k(n)^{\theta_k}$.*

Proof. As we already know that $\mathfrak{a}_k(n) \subseteq \mathfrak{g}_k(n)$, we only need to check that $\theta_k(g) = g$ for all generators g of $\mathfrak{a}_k(n)$. By conjugation, it is equivalent to check that $\tilde{\theta}_k(g) = g$ for the generators g of $\tilde{\mathfrak{a}}_k(n)$. Applying φ_n to the generators of $\mathfrak{a}_3(n)$, we find that the generators of $\tilde{\mathfrak{a}}_3(n)$ are:

$$X_1X_2, X_2X_3, X_3X_4, X_4X_5, X_5X_6, X_6X_7, X_7X_8, \ldots,$$
$$Y_1Y_2, Z_2Z_3, Y_3Y_4, Z_4Z_5, Y_5Y_6, Z_6Z_7, Y_7Y_8, \ldots.$$

Similarly, applying γ_n to the generators of $\mathfrak{a}_5(n)$, we find the generators of $\tilde{\mathfrak{a}}_5(n)$:

$$Z_1Z_2, Y_2Y_3, X_3X_4, Z_4Z_5, Y_5Y_6, X_6X_7, Z_7Z_8, Y_8Y_9, X_9X_{10}, \ldots,$$
$$X_1X_2, Z_2Z_3, Y_3Y_4, X_4X_5, Z_5Z_6, Y_6Y_7, X_7X_8, Z_8Z_9, Y_9Y_{10}, \ldots.$$

We observe that all generators g above satisfy $g^T = g$ and $gQ_k = -Q_k g$; hence, $\tilde{\theta}_k(g) = g$. □

VI. Lower bounds for $\mathfrak{a}_k(n)$

In this subsection, we prove that the upper bounds $\mathfrak{a}_k(n) \subseteq \mathfrak{g}_k(n)^{\theta_k}$ established in Sect. C V are exact. The proof will rely on the next lemma.

Lemma C.17. *Let $\mathfrak{s}$ be a Lie subalgebra of $\mathfrak{su}(2^n)$. If $\mathrm{ad}_{a_1} \cdots \mathrm{ad}_{a_r}(b) \in \mathfrak{s} \setminus \{0\}$ for some Pauli strings $a_1, \dots, a_r \in i\mathcal{P}_n \cap \mathfrak{s}$ and $b \in i\mathcal{P}_n$, then $b \in \mathfrak{s}$.*

Proof. Using induction on r, it is enough to prove the statement for $r = 1$. In this case, it follows from Corollary A.1: $[a_1, b] \in \mathfrak{s} \setminus \{0\}$ implies that $b = -4[a_1, [a_1, b]] \in \mathfrak{s}$. □

In order to prove that $\mathfrak{a}_k(n) = \mathfrak{g}_k(n)^{\theta_k}$, we want to show that every element $b \in \mathfrak{g}_k(n)^{\theta_k}$ is in $\mathfrak{a}_k(n)$. Since, by Corollary C.2, b is a linear combination of Pauli strings that are themselves in $\mathfrak{g}_k(n)^{\theta_k}$, we can assume without loss of generality that $b \in i\mathcal{P}_n \cap \mathfrak{g}_k(n)^{\theta_k}$. Then the strategy of the proof is to take suitable commutators of b with elements of $i\mathcal{P}_n \cap \mathfrak{a}_k(n)$ to produce a Pauli string $c \in i\mathcal{P}_n \cap \mathfrak{g}_k(n)^{\theta_k}$ that has I in one of its positions. Erasing the I will give an element of $\mathfrak{g}_k(n-1)^{\theta_k}$, which by induction will be in $\mathfrak{a}_k(n-1)$. From here, we will obtain that $c \in \mathfrak{a}_k(n)$, and then we can conclude that $b \in \mathfrak{a}_k(n)$ due to Lemma C.17.

In order to realize the above strategy, we will have to do a detailed case-by-case analysis. We start with the cases $k = 3, 5, 7$, for which we need the following lemmas.

Lemma C.18. *We have vector space decompositions:*

$$\begin{aligned}
\mathfrak{a}_3(4) &= (I \otimes \mathfrak{a}_3(3)) + P_X \cdot (I \otimes \mathfrak{a}_3(3)) + P_{YZ} \cdot (I \otimes \mathfrak{a}_3(3)) + P_{ZY} \cdot (I \otimes \mathfrak{a}_3(3)), \\
\mathfrak{a}_5(6) &= (I \otimes \mathfrak{a}_5(5)) + P_{XYZ} \cdot (I \otimes \mathfrak{a}_5(5)) + P_{YZX} \cdot (I \otimes \mathfrak{a}_5(5)) + P_{ZXY} \cdot (I \otimes \mathfrak{a}_5(5)), \\
\mathfrak{a}_7(4) &= (I \otimes \mathfrak{a}_7(3)) + P_X \cdot (I \otimes \mathfrak{a}_7(3)) + P_Y \cdot (I \otimes \mathfrak{a}_7(3)) + P_Z \cdot (I \otimes \mathfrak{a}_7(3)),
\end{aligned}$$

where $\cdot$ denotes the componentwise matrix product.

Proof. The proof is done by inspection, making use of our code [4]. Here are all Pauli strings in $\mathfrak{a}_3(4)$ (after multiplication by i):

$$\begin{array}{llllllllll}
IIXX, & IIYZ, & IXXI, & IXZZ, & IYIY, & IYXZ, & IYYX, & IYZI, & IZIZ, & IZXY, \\
XIIX, & XIYY, & XXII, & XXZY, & XYIZ, & XYXY, & XZIY, & XZXZ, & XZYX, & XZZI, \\
YIYI, & YIZX, & YXIY, & YXXZ, & YXYX, & YXZI, & YYXI, & YYZZ, & YZII, & YZZY, \\
ZIIY, & ZIXZ, & ZIYX, & ZIZI, & ZXYI, & ZXZX, & ZYXX, & ZYYZ, & ZZIX, & ZZYY.
\end{array}$$

The Pauli strings in $\mathfrak{a}_7(4)$ (after multiplication by i) are:

$$\begin{array}{llllllll}
IIXX, & IIYY, & IIZZ, & IXIX, & IXXI, & IXYZ, & IXZY, & IYIY, \\
IYXZ, & IYYI, & IYZX, & IZIZ, & IZXY, & IZYX, & IZZI, & \\
XIIX, & XIXI, & XIYZ, & XIZY, & XXII, & XXYY, & XXZZ, & XYIZ, \\
XYXY, & XYYX, & XYZI, & XZIY, & XZXZ, & XZYI, & XZZX, & \\
YIIY, & YIXZ, & YIYI, & YIZX, & YXIZ, & YXXY, & YXYX, & YXZI, \\
YYII, & YYXX, & YYZZ, & YZIX, & YZXI, & YZYZ, & YZZY, & \\
ZIIZ, & ZIXY, & ZIYX, & ZIZI, & ZXIY, & ZXXZ, & ZXYI, & ZXZX, \\
ZYIX, & ZYXI, & ZYYZ, & ZYZY, & ZZII, & ZZXX, & ZZYY. &
\end{array}$$

We have: $|\mathfrak{a}_5(5)| = 120$, $|\mathfrak{a}_5(6)| = 480$, and there are 120 elements in $\mathfrak{a}_5(6)$ starting with each of the letters I, X, Y, or Z. The remaining claims were verified using Excel. □

For each pair $(k, n) = (3, 4), (5, 6), (7, 4)$, consider the subalgebra $\mathfrak{s} = \mathfrak{a}_k(n) \subset \mathfrak{su}(2^n)$. Recall from Proposition C.3 that the stabilizer $\mathrm{St}(\mathfrak{s})$ is given by:

$$\begin{aligned}
\mathrm{St}(\mathfrak{a}_3(4)) &= \{P_I, P_X, P_{YZ}, P_{ZY}\}, \\
\mathrm{St}(\mathfrak{a}_5(6)) &= \{P_I, P_{XYZ}, P_{YZX}, P_{ZXY}\}, \\
\mathrm{St}(\mathfrak{a}_7(4)) &= \{P_I, P_X, P_Y, P_Z\},
\end{aligned}$$

and $\mathrm{St}(\mathfrak{s})$ is an Abelian group under the matrix product $\cdot$. We can state Lemma C.18 succinctly as

$$\mathfrak{a}_k(n) = \mathrm{St}(\mathfrak{a}_k(n)) \cdot (I \otimes \mathfrak{a}_k(n-1)), \qquad (k, n) = (3, 4), (5, 6), (7, 4). \tag{C41}$$

Lemma C.19. *Let $\mathfrak{s} = \mathfrak{a}_k(n)$ for $(k, n) = (3, 4), (5, 6), (7, 4)$. Consider any Pauli string $a \in i\mathcal{P}_n \cap \mathfrak{g}_k(n)$ not starting with I in the first qubit. Then there exists a basis vector $b \in \mathfrak{s}$ such that $[a, b] \neq 0$ and $[a, b] \in I \otimes \mathfrak{su}(2^{n-1})$.*

Proof. Let us write all Pauli strings up to a suitable multiple of i that makes them skew-Hermitian. Consider the case where $a = XA$ starts with X; the cases when it starts with Y or Z are similar. If there is $b = XB \in \mathfrak{s}$ such that $[A, B] \neq 0$, then $[a, b] = I[A, B] \neq 0$ and we are done. By Lemma C.18, any $b = XB \in \mathfrak{s}$ can be written in the form $b = C \cdot (ID)$, where $D \in \mathfrak{a}_k(n-1)$ and $C \in \mathrm{St}(\mathfrak{s})$; explicitly, $C = P_X, P_{XYZ}, P_X$ for $\mathfrak{s} = \mathfrak{a}_3(4), \mathfrak{a}_5(6), \mathfrak{a}_7(4)$, respectively. Similarly, as $C \cdot C = P_I$, we can write $a = C \cdot (IE)$ for some $E \in \mathcal{P}_{n-1}$. Suppose that $[a, b] = 0$. Then $[C, a] = [C, b] = 0$ imply that $[E, D] = 0$. Since this is true for all $D \in \mathfrak{a}_k(n-1)$, it follows that $E \in \mathrm{St}(\mathfrak{a}_k(n-1))$, from where $a = C \cdot (IE) \in \mathrm{St}(\mathfrak{s})$. This is a contradiction, because such elements are factored out from $\mathfrak{g}_k(n)$; see (C23) and Lemma C.13. ☐

Lemma C.20. *Consider $(k, m) = (3, 4), (5, 6), (7, 4)$, and let $n \geq m$. Then for any Pauli string $a \in i\mathcal{P}_n \cap \mathfrak{g}_k(n)$, there exist basis vectors $b_1, \dots, b_r \in \mathfrak{a}_k(n)$, $r \geq 0$, such that $\mathrm{ad}_{b_1} \cdots \mathrm{ad}_{b_r}(a) \in (I^{\otimes(n-m+1)} \otimes \mathfrak{su}(2^{m-1})) \setminus \{0\}$ (with $r = 0$ corresponding to a).*

Proof. The proof is by induction on n, the base $n = m$ being Lemma C.19. For the step of the induction, suppose that $n > m$ and the statement holds for $\mathfrak{g}_k(n-1)$. Again, let us write all Pauli strings up to a suitable multiple of i. Take any Pauli string $a \in \mathfrak{g}_k(n)$, and write it as $a = AD$ where A is the substring consisting of the first m Paulis. Then $A \in \mathfrak{su}(2^m)^{\mathrm{St}(\mathfrak{a}_k(m))}$.

If $A \notin \mathrm{St}(\mathfrak{a}_k(m))$, we can use Lemma C.19 to find $B \in \mathfrak{a}_k(m)$ such that $[B, A]$ starts with I. Then $[b, a] = IC$ starts with I for $b = BI \cdots I \in \mathfrak{a}_k(n)$. After that, we can apply the inductive assumption for $C \in \mathfrak{g}_k(n-1)$.

If $A \in \mathrm{St}(\mathfrak{a}_k(m))$, we repeat the same argument for the substring E of a corresponding to positions $2, \dots, m+1$. When $E \notin \mathrm{St}(\mathfrak{a}_k(m))$, we can make it to start with I, which will force the substring $A \notin \mathrm{St}(\mathfrak{a}_k(m))$. If both $A, E \in \mathrm{St}(\mathfrak{a}_k(m))$, putting them together we get that the first $m+1$ positions of a are in $\mathrm{St}(\mathfrak{a}_k(m+1))$. Continuing this way will give us $a \in \mathrm{St}(\mathfrak{a}_k(n))$, which is a contradiction, because such elements are factored out from $\mathfrak{g}_k(n)$ (cf. (C23) and Lemma C.13). ☐

Recall that, in Sect. C II, we constructed certain automorphisms φ_n and γ_n of $\mathfrak{su}(2^n)$ such that the images $\tilde{\mathfrak{a}}_3(n) := \varphi_n \mathfrak{a}_3(n)$ and $\tilde{\mathfrak{a}}_5(n) := \gamma_n \mathfrak{a}_5(n)$ are subalgebras of $\mathfrak{a}_7(n)$. Note that, after these transformations, their stabilizers become equal:

$$\mathrm{St}(\tilde{\mathfrak{a}}_3(n)) = \mathrm{St}(\tilde{\mathfrak{a}}_5(n)) = \mathrm{St}(\mathfrak{a}_7(n)) = \{P_I, P_X, P_Y, P_Z\}. \tag{C42}$$

Hence, we have (recall (C37)–(C40)):

$$\tilde{\mathfrak{a}}_k(n) \subseteq \mathfrak{g}_7(n)^{\tilde{\theta}_k}, \qquad k = 3, 5. \tag{C43}$$

Lemma C.21. *We have $\mathfrak{a}_7(n) = \mathfrak{g}_7(n)$ and equalities in (C43). Consequently, $\mathfrak{a}_k(n) = \mathfrak{g}_k(n)^{\theta_k}$ for $k = 3, 5$.*

Proof. As before, let $(k, m) = (3, 4), (5, 6), (7, 4)$. In order to include the case $k = 7$ in (C43), we let $\tilde{\mathfrak{a}}_7(n) = \mathfrak{a}_7(n)$ and $\tilde{\theta}_7$ be the identity. The statement is true for all $2 \leq n \leq m$ by inspection. For $n \geq m$, we prove it by induction on n. Consider any $a \in \mathfrak{g}_7(n)^{\tilde{\theta}_k}$. By Lemma C.20, we can find $b_1, \dots, b_r \in \tilde{\mathfrak{a}}_k(n)$ such that $\mathrm{ad}_{b_1} \cdots \mathrm{ad}_{b_r}(a) = I \cdots ID$ for some $D \in \mathfrak{su}(2^{m-1}) \setminus \{0\}$. Since $b_i \in \tilde{\mathfrak{a}}_k(n) \subseteq \mathfrak{g}_7(n)^{\tilde{\theta}_k}$, we get that $D \in \mathfrak{g}_7(m)^{\tilde{\theta}_k} = \tilde{\mathfrak{a}}_k(m)$. Therefore, $a \in \tilde{\mathfrak{a}}_k(n)$ due to Lemma C.17. ☐

Remark C.8. *It follows from Lemma C.21 that (C41) holds for all (k, n) such that: $k = 3$, $n \equiv 0 \mod 4$; $k = 5$, $n \equiv 0 \mod 6$; $k = 7$, $n \equiv 0 \mod 2$. As a consequence, for such (k, n), we have $\mathfrak{a}_k(n) \cong \mathfrak{a}_k(n-1)^{\oplus 4}$ as a Lie algebra.*

Now that we are done with the cases $k = 3, 5, 7$, we derive the cases $k = 6, 10$ from $k = 7$ and the isomorphisms $\mathfrak{a}_6(n) \cong \mathfrak{a}_{10}(n) \cong \mathfrak{a}_7(n)$ obtained in Lemmas C.4 and C.5.

Lemma C.22. *We have $\mathfrak{a}_k(n) = \mathfrak{g}_k(n)$ for $k = 6, 10$.*

Proof. Recall from Sect. C II that we have an isomorphism $\varphi_n \colon \mathfrak{a}_6(n) \cong \mathfrak{a}_7(n)$ that up to a sign swaps $Y \rightleftharpoons Z$ on every even qubit (see (C8), (C9)). Under φ_n the stabilizers

$$\mathrm{St}(\mathfrak{a}_6(n)) = \{P_I, P_X, P_{YZ}, P_{ZY}\},$$
$$\mathrm{St}(\mathfrak{a}_7(n)) = \{P_I, P_X, P_Y, P_Z\}$$

are sent to each other; hence $\mathfrak{g}_6(n) \cong \mathfrak{g}_7(n)$. Since $\mathfrak{a}_7(n) = \mathfrak{g}_7(n)$ by Lemma C.21, it follows that $\mathfrak{a}_6(n) = \mathfrak{g}_6(n)$.

Similarly, we have an isomorphism $\gamma_n \colon \mathfrak{a}_{10}(n) \cong \mathfrak{a}_7(n)$ given by applying on the j-th qubit ($j = 1, \dots, n$) the permutation γ^j, where γ is the cycle $X \mapsto Z \mapsto Y \mapsto X$ (see (C11), (C12)). Then γ_n sends

$$\mathrm{St}(\mathfrak{a}_{10}(n)) = \{P_I, P_{XYZ}, P_{YZX}, P_{ZXY}\}$$

to $\mathrm{St}(\mathfrak{a}_7(n))$; hence $\mathfrak{g}_{10}(n) \cong \mathfrak{g}_7(n)$ and $\mathfrak{a}_{10}(n) = \mathfrak{g}_{10}(n)$. □

Now we consider the subalgebra $\mathfrak{a}_9(n)$. In this case, we have the involution $\theta_9(g) = -Q_9 g^T Q_9$, where Q_9 is given by (C35).

Lemma C.23. *We have* $\mathfrak{a}_9(n) = \mathfrak{g}_9(n)^{\theta_9}$.

Proof. The claim is true for $n = 2$ and 3 by comparing the dimensions. Suppose by induction that the statement is true for $\mathfrak{a}_9(n-1)$, and consider a Pauli string $a \in \mathfrak{g}_9(n)^{\theta_9}$ for $n \geq 4$. Again, we will omit the multiples of i that make Pauli strings skew-Hermitian.

If a ends with I, we can write $a = AI$ for some $A \in \mathfrak{g}_9(n-1)^{\theta_9}$ and apply the inductive assumption. Similarly, if $a = AIB$ has an I in the j-th position for some $j \geq 3$, we can delete it and get an element $AB \in \mathfrak{g}_9(n-1)^{\theta_9}$, which by induction is in $\mathfrak{a}_9(n-1)$. Then $a \in \mathfrak{a}_9(n)$, because $\mathfrak{a}_9(4)$ contains $IXIY$ and $IXIZ$, which generate elements of $\mathfrak{a}_9(n)$ with I in the middle.

Suppose that a has no I in positions $3, \dots, n$. If $a = AXXB$ contains XX in positions $j, j+1$, then $[X_j Y_{j+1}, a] = -2iAIZB \in \mathfrak{a}_9(n)$. Since $X_j Y_{j+1} \in \mathfrak{a}_9(n)$, by Lemma C.17, we get that $a \in \mathfrak{a}_9(n)$. So, if $a \notin \mathfrak{a}_9(n)$ contains an X, then on the left of it must have a Y or Z. Then we can use $[XZ, XY] = -2iIX$, $[XY, YY] = 2iZI$, $[XY, ZY] = -2iYI$ when a contains a Y, and $[XY, XZ] = 2iIX$, $[XZ, YZ] = 2iZI$, $[XZ, ZZ] = -2iYI$ when a contains a Z. □

Finally, let us briefly discuss the remaining easier cases, $k = 11, 13, 15, 16, 20$. Recall from (C5), (C6) that $\mathfrak{a}_{11}(n) = \mathfrak{a}_{16}(n)$ for $n \geq 4$ and $\mathfrak{a}_{13}(n) = \mathfrak{a}_{20}(n)$ for $n \geq 3$. Moreover, $\mathfrak{g}_{11}(n) = \mathfrak{g}_{16}(n)$ and $\mathfrak{g}_{13}(n) = \mathfrak{g}_{20}(n)$, because they have equal stabilizers by Proposition C.3. Thus, we are left to consider only $k = 13, 15, 16$.

Lemma C.24. *We have* $\mathfrak{a}_{13}(n) = \mathfrak{g}_{13}(n) = \mathfrak{su}(2^n)^{P_X}/\mathrm{span}\{P_X\}$ *for* $n \geq 3$.

Proof. We know that $\mathrm{St}(\mathfrak{a}_{13}(n)) = \{P_I, P_X\}$ and $\mathfrak{a}_{13}(n) \subseteq \mathfrak{g}_{13}(n)$. The proof of the opposite inclusion is similar to the proof of Lemma C.23. Consider a Pauli string $a \in \mathfrak{g}_{13}(n)$ for $n \geq 4$. If $a = AIB$ has an I in the j-th position for some $1 \leq j \leq n$, we can delete it and get an element $AB \in \mathfrak{g}_{13}(n-1)$, which by induction is in $\mathfrak{a}_{13}(n-1)$. Then $a \in \mathfrak{a}_{13}(n)$, because $\mathfrak{a}_{13}(3)$ contains XIX, YIY, YIZ, and these generate elements of $\mathfrak{a}_{13}(n)$ with I in the middle. If a has no I's, we can use commutators with the generators of $\mathfrak{a}_{13}(n)$ to produce one. Then again we can apply Lemma C.17. □

Lemma C.25. *We have* $\mathfrak{a}_{16}(n) = \mathfrak{su}(2^n)^{\theta_{16}} = \mathfrak{so}(2^n)$, *where* $\theta_{16}(g) = -g^T$.

Proof. The same as the proof of Lemma C.24, using that $AIB \in \mathfrak{a}_{16}(3)$ for every generator AB of $\mathfrak{a}_{16}$. Indeed, one checks that $\mathfrak{a}_{16} = \langle XY, YX, YZ, ZY \rangle_{\mathrm{Lie}}$ and $XIY, YIX, YIZ, ZIY \in \mathfrak{a}_{16}(3)$; see Sect. B V. □

Lemma C.26. *We have* $\mathfrak{a}_{15}(n) = \mathfrak{g}_{15}(n) = \mathfrak{su}(2^n)^{X_1}/\mathrm{span}\{X_1\}$.

Proof. Note that $\mathfrak{su}(2^n)^{X_1}$ is the span of all Pauli strings $\neq I^{\otimes n}$ that start with I or X. As in the proof of Lemma C.24, pick any Pauli string $a \in \mathfrak{g}_{15}(n)$ for $n \geq 3$. If $a = AIB$ has an I in the j-th position for some $2 \leq j \leq n$, we can delete it and get an element $AB \in \mathfrak{g}_{15}(n-1)$, which by induction is in $\mathfrak{a}_{15}(n-1)$. The rest of the proof is the same, using that $\mathfrak{a}_{15} = \langle XX, XY, XZ \rangle_{\mathrm{Lie}}$ and $XIX, XIY, XIZ \in \mathfrak{a}_{15}(3)$; see Sect. B V. □

Combining the results of Sect. C V and C VI completes the proof of Theorem C.1.

VII. Identifying the Lie algebras $\mathfrak{g}_k(n)^{\theta_k}$

In this subsection, we finish the proof of Theorem IV.1, by identifying the Lie algebras $\mathfrak{g}_k(n)^{\theta_k}$ from Theorem C.1 with the Lie algebras appearing in the right-hand sides in Theorem IV.1. As in Theorem C.1, we only consider the cases $k = 3, 5, 6, 7, 9, 10, 11, 13, 15, 16, 20$. Moreover, due to the isomorphisms $\mathfrak{a}_6(n) \cong \mathfrak{a}_7(n) \cong \mathfrak{a}_{10}(n)$ and the equalities $\mathfrak{a}_{11}(n) = \mathfrak{a}_{16}(n)$ and $\mathfrak{a}_{13}(n) = \mathfrak{a}_{20}(n)$ (see Lemmas C.2, C.4, C.5), we can omit the cases $k = 6, 10, 11, 20$.

The case $k = 16$ is obvious, because $\mathfrak{g}_{16}(n) = \mathfrak{su}(2^n)$ and $\theta_{16}(g) = -g^T$, leading to $\mathfrak{a}_{16}(n) = \mathfrak{so}(2^n)$. Two other easy cases, $k = 15$ and $k = 13$, are treated in the next lemma.

Lemma C.27. *We have:*

$$\mathfrak{a}_{15}(n) = \mathfrak{g}_{15}(n) = \mathfrak{su}(2^n)^{X_1}/\mathrm{span}\{X_1\} \cong \mathfrak{su}(2^{n-1}) \oplus \mathfrak{su}(2^{n-1}),$$
$$\mathfrak{a}_{13}(n) = \mathfrak{g}_{13}(n) = \mathfrak{su}(2^n)^{P_X}/\mathrm{span}\{P_X\} \cong \mathfrak{su}(2^{n-1}) \oplus \mathfrak{su}(2^{n-1}).$$

Proof. Note that

$$\mathfrak{su}(2^n)^{X_1}/\mathrm{span}\{X_1\} \cong \mathrm{span}_{\mathbb{R}}\{I, X\} \otimes \mathfrak{su}(2^{n-1})$$

has a basis consisting of all Pauli strings $\neq I^{\otimes n}, X_1$ that start with I or X. Consider the projections $P_\pm$ onto the eigenspaces of X, given by $P_\pm := (I \pm X)/2$. They satisfy the identities:

$$P_\pm \cdot P_\pm = P_\pm, \qquad P_+ \cdot P_- = 0, \qquad P_+ + P_- = I.$$

Then the map

$$(a, b) \mapsto P_+ \otimes a + P_- \otimes b$$

is a Lie algebra isomorphism from $\mathfrak{su}(2^{n-1}) \oplus \mathfrak{su}(2^{n-1})$ to $\mathrm{span}_{\mathbb{R}}\{I, X\} \otimes \mathfrak{su}(2^{n-1})$. This proves the claim about $\mathfrak{a}_{15}(n)$.

For the case $\mathfrak{a}_{13}(n)$, we can replace X_1 with P_X because there exists a unitary transformation U such that $P_X = UX_1U^\dagger$. For example, we can take

$$U = e^{-i\frac{\pi}{4}Y_1} e^{i\frac{\pi}{4}Y \otimes X^{\otimes(n-1)}};$$

then using (A6) we check that indeed $UX_1U^\dagger = X \otimes X^{\otimes(n-1)} = P_X$. The automorphism $a \mapsto UaU^\dagger$ of $\mathfrak{su}(2^n)$ sends $\mathfrak{su}(2^n)^{X_1}$ onto $\mathfrak{su}(2^n)^{P_X}$, and $\mathfrak{a}_{15}(n)$ onto $\mathfrak{a}_{13}(n)$. Therefore, $\mathfrak{a}_{13}(n) \cong \mathfrak{a}_{15}(n)$. □

We are left with the cases $k = 3, 5, 7, 9$, and we consider $k = 9$ next.

Lemma C.28. *We have* $\mathfrak{a}_9(n) = \mathfrak{g}_9(n)^{\theta_9} \cong \mathfrak{sp}(2^{n-2})$.

Proof. Recall that $\mathfrak{g}_9(n) = \mathfrak{su}(2^n)^{\{X_1, Y_1X_2, Z_1X_2\}}$. Since $\mathrm{span}\{X_1, Y_1X_2, Z_1X_2\} \cong \mathfrak{su}(2)$, we can find a unitary transformation that takes this Lie algebra to $\mathrm{span}\{X_1, Y_1, Z_1\}$. Explicitly, similarly to the proof of Lemma C.27, let $U = e^{i\frac{\pi}{4}X_1X_2}$. Then using (A6), one easily checks that

$$UX_1U^\dagger = X_1, \qquad UY_1X_2U^\dagger = -Z_1, \qquad UZ_1X_2U^\dagger = Y_1.$$

Therefore, the map $a \mapsto UaU^\dagger$ restricts to a Lie algebra isomorphism from $\mathfrak{g}_9(n)$ to

$$\mathfrak{su}(2^n)^{\{X_1, Y_1, Z_1\}} = I \otimes \mathfrak{su}(2^{n-1}) \cong \mathfrak{su}(2^{n-1}).$$

According to Lemmas A.3 and A.4, under the transformation $a \mapsto UaU^\dagger$, the fixed-point subalgebra $\mathfrak{g}_9(n)^{\theta_9}$ is sent to the fixed points of the following involution:

$$a \mapsto -(UQ_9U^T)a^T(UQ_9U^T)^\dagger.$$

Recalling that $Q_9 = Y_2Z_3 \cdots Z_n$ (see (C35)), we find from $U^T = U$ and $e^{i\frac{\pi}{4}X} Y e^{i\frac{\pi}{4}X} = Y$ that

$$UQ_9U^T = Q_9.$$

Hence, the image of $\mathfrak{g}_9(n)^{\theta_9}$ under $a \mapsto UaU^\dagger$ consists of all $b \in I \otimes \mathfrak{su}(2^{n-1})$ that are fixed by θ_9. Writing $b = I \otimes c$ with $c \in \mathfrak{su}(2^{n-1})$, the condition $b = \theta_9(b)$ is equivalent to $c = -Qc^TQ$, where $Q = YZ \cdots Z \in \mathcal{P}_{n-1}$. Since $Q^T = -Q$, this determines the Lie algebra $\mathfrak{sp}(2^{n-2})$, due to Corollary A.3. □

Next we consider the case $k = 7$.

Lemma C.29. *We have* $\mathfrak{a}_7(n) = \mathfrak{g}_7(n) \cong \begin{cases} \mathfrak{su}(2^{n-1}), & n \text{ odd}, \\ \mathfrak{su}(2^{n-2})^{\oplus 4}, & n \geq 4 \text{ even}. \end{cases}$

Proof. Recall that $\mathrm{St}(\mathfrak{a}_7(n)) = \{P_I, P_X, P_Y, P_Z\}$. Since $P_X \cdot P_Y = i^n P_Z$, elements that commute with P_X and P_Y will commute with P_Z as well. Hence, $\mathfrak{su}(2^n)^{\mathrm{St}(\mathfrak{a}_7(n))} = \mathfrak{su}(2^n)^{\{P_X,P_Y\}}$. Recall also that $[P_X, P_Y] = 0$ if and only if n is even; in that case, $\mathfrak{su}(2^n)^{\{P_X,P_Y\}}$ has a center spanned by P_X, P_Y, P_Z and we need to quotient by it to obtain $\mathfrak{g}_7(n)$ (cf. (C27)).

In order to determine the fixed points under P_X and P_Y, we transform them as in the proof of Lemma C.27. Consider the unitary operator

$$U = \begin{cases} e^{i\frac{\pi}{4} Z\otimes Y^{\otimes(n-1)}} e^{i\frac{\pi}{4} Y\otimes X^{\otimes(n-1)}}, & n \text{ odd}, \\ e^{i\frac{\pi}{4} X_2} e^{i\frac{\pi}{4} I\otimes X\otimes Z^{\otimes(n-2)}} e^{i\frac{\pi}{4} Y\otimes X^{\otimes(n-1)}}, & n \text{ even}. \end{cases} \tag{C44}$$

Using (A6), one checks that

$$UP_XU^\dagger = Z_1, \qquad UP_YU^\dagger = X_1 \qquad \text{for } n \text{ odd}, \tag{C45}$$

$$UP_XU^\dagger = Z_1, \qquad UP_YU^\dagger = (-1)^{(n+2)/2} Z_2 \qquad \text{for } n \text{ even}. \tag{C46}$$

Indeed, we have

$$e^{i\frac{\pi}{4} Y\otimes X^{\otimes(n-1)}} P_X e^{-i\frac{\pi}{4} Y\otimes X^{\otimes(n-1)}} = i(Y \otimes X^{\otimes(n-1)}) \cdot P_X = Z_1.$$

Since the other factors of U commute with Z_1, we obtain that $UP_XU^\dagger = Z_1$. The calculation of $UP_YU^\dagger$ is similar. When n is odd, $Y \otimes X^{\otimes(n-1)}$ commutes with P_Y, and we get from (A6):

$$UP_YU^\dagger = e^{i\frac{\pi}{4} Z\otimes Y^{\otimes(n-1)}} P_Y e^{-i\frac{\pi}{4} Z\otimes Y^{\otimes(n-1)}} = i(Z \otimes Y^{\otimes(n-1)}) \cdot P_Y = X_1.$$

When n is even, after applying (A6) three times, we obtain:

$$\begin{aligned} UP_YU^\dagger &= i^3 X_2 \cdot (I \otimes X \otimes Z^{\otimes(n-2)}) \cdot (Y \otimes X^{\otimes(n-1)}) \cdot P_Y \\ &= i^{n+2} X_2 \cdot (I \otimes X \otimes Z^{\otimes(n-2)}) \cdot (I \otimes Z^{\otimes(n-1)}) \\ &= i^{n+1} X_2 \cdot Y_2 = i^{n+2} Z_2. \end{aligned}$$

This proves (C45) and (C46).

It follows from (C45) that, for n odd, the map $a \mapsto UaU^\dagger$ gives a Lie algebra isomorphism

$$\mathfrak{g}_7(n) = \mathfrak{su}(2^n)^{\{P_X,P_Y\}} \to \mathfrak{su}(2^n)^{\{X_1,Z_1\}} = I \otimes \mathfrak{su}(2^{n-1}) \cong \mathfrak{su}(2^{n-1}).$$

Now suppose that n is even. Then, by (C46), the map $a \mapsto UaU^\dagger$ gives an isomorphism

$$\mathfrak{su}(2^n)^{\{P_X,P_Y\}} \to \mathfrak{su}(2^n)^{\{Z_1,Z_2\}} = \big(\mathrm{span}_{\mathbb{R}}\{I,Z\} \otimes \mathrm{span}_{\mathbb{R}}\{I,Z\} \otimes \mathfrak{su}(2^{n-2})\big) \oplus \mathrm{span}\{Z_1, Z_2, Z_1Z_2\}.$$

After we quotient by the center $\mathrm{span}\{Z_1, Z_2, Z_1Z_2\}$, we obtain

$$\mathfrak{g}_7(n) = \mathfrak{su}(2^n)^{\{P_X,P_Y\}}/\mathrm{span}\{P_X, P_Y, P_Z\} \cong \mathrm{span}_{\mathbb{R}}\{I,Z\} \otimes \mathrm{span}_{\mathbb{R}}\{I,Z\} \otimes \mathfrak{su}(2^{n-2}).$$

Again as in the proof of Lemma C.27, let $P_\pm := (I \pm Z)/2$, and consider the four projections

$$P_1 := P_+ \otimes P_+, \qquad P_2 := P_+ \otimes P_-, \qquad P_3 := P_- \otimes P_+, \qquad P_4 := P_- \otimes P_-,$$

which satisfy

$$P_i \cdot P_i = P_i, \qquad P_i \cdot P_j = 0 \quad (i \neq j), \qquad \sum_{i=1}^{4} P_i = I \otimes I.$$

Then the linear map

$$(a_1, a_2, a_3, a_4) \mapsto \sum_{j=1}^{4} P_j \otimes a_j \tag{C47}$$

is an isomorphism from $\mathfrak{su}(2^{n-2})^{\oplus 4}$ to $\mathrm{span}_{\mathbb{R}}\{I,Z\} \otimes \mathrm{span}_{\mathbb{R}}\{I,Z\} \otimes \mathfrak{su}(2^{n-2})$. □

In the remaining two cases $k = 3, 5$, as before we embed $\mathfrak{a}_3(n)$ and $\mathfrak{a}_5(n)$ as subalgebras of $\mathfrak{a}_7(n)$. We continue to use the notation from Sect. C VI and, as in Lemma C.29, we consider separately the cases when n is odd or even.

Lemma C.30. *We have* $\mathfrak{a}_3(n) \cong \tilde{\mathfrak{a}}_3(n) = \mathfrak{g}_7(n)^{\tilde{\theta}_3} \cong \begin{cases} \mathfrak{so}(2^{n-1}), & n \equiv \pm 1 \bmod 8, \\ \mathfrak{sp}(2^{n-2}), & n \equiv \pm 3 \bmod 8. \end{cases}$

Proof. We apply the transformation $a \mapsto UaU^\dagger$ from the proof of Lemma C.29 that gives a Lie algebra isomorphism $\mathfrak{g}_7(n) \to I \otimes \mathfrak{su}(2^{n-1}) \cong \mathfrak{su}(2^{n-1})$, where U is defined by (C44) for odd n. Then, by Lemmas A.3, A.4, the fixed points of $\tilde{\theta}_k$ (see (C37)) are sent to the fixed points of the involution

$$g \mapsto -(UQ_kU^T)g^T(UQ_kU^T)^\dagger, \qquad k = 3, 5. \tag{C48}$$

Recall that $Q_3 = P_{ZIYX}$ is given by (C38), and compute

$$\tilde{Q}_3 := UQ_3U^T = e^{i\frac{\pi}{4}Z\otimes Y^{\otimes(n-1)}} e^{i\frac{\pi}{4}Y\otimes X^{\otimes(n-1)}} P_{ZIYX} e^{-i\frac{\pi}{4}Y\otimes X^{\otimes(n-1)}} e^{i\frac{\pi}{4}Z\otimes Y^{\otimes(n-1)}}.$$

Note that, when $n \equiv 1 \bmod 4$, P_{ZIYX} anticommutes with $Y \otimes X^{\otimes(n-1)}$. By (A6), we have:

$$\begin{aligned} &e^{i\frac{\pi}{4}Y\otimes X^{\otimes(n-1)}} P_{ZIYX} e^{-i\frac{\pi}{4}Y\otimes X^{\otimes(n-1)}} \\ &\qquad = i(Y_1X_2X_3X_4\cdots X_{n-1}X_n)\cdot(Z_1Y_3X_4Z_5Y_7X_8\cdots X_{n-1}Z_n) \\ &\qquad = -X_1(X_2Z_3Y_5)(X_6Z_7Y_9)\cdots(X_{n-3}Z_{n-2}Y_n). \end{aligned}$$

As this anticommutes with $e^{i\frac{\pi}{4}Z\otimes Y^{\otimes(n-1)}}$, we obtain

$$\tilde{Q}_3 = -X_1(X_2Z_3Y_5)(X_6Z_7Y_9)\cdots(X_{n-3}Z_{n-2}Y_n), \qquad n \equiv 1 \bmod 4.$$

Hence, restricted to elements $g = I \otimes c$ with $c \in \mathfrak{su}(2^{n-1})$, the involution (C48) becomes:

$$c \mapsto -P_{XZIY}c^TP_{XZIY}, \qquad \text{for} \quad n \equiv 1 \bmod 4.$$

For the fixed-point subalgebra, we obtain from Corollary A.3:

$$(P_{XZIY})^T = \begin{cases} P_{XZIY}, & n \equiv 1 \bmod 8, \\ -P_{XZIY}, & n \equiv 5 \bmod 8 \end{cases} \quad \Rightarrow \quad \tilde{\mathfrak{a}}_3(n) \cong \begin{cases} \mathfrak{so}(2^{n-1}), & n \equiv 1 \bmod 8, \\ \mathfrak{sp}(2^{n-2}), & n \equiv 5 \bmod 8. \end{cases}$$

Alternatively, when $n \equiv 3 \bmod 4$, P_{ZIYX} commutes with both $Y \otimes X^{\otimes(n-1)}$ and $Z \otimes Y^{\otimes(n-1)}$. Hence, in this case,

$$\begin{aligned} \tilde{Q}_3 &= e^{i\frac{\pi}{4}Z\otimes Y^{\otimes(n-1)}} P_{ZIYX} e^{i\frac{\pi}{4}Z\otimes Y^{\otimes(n-1)}} \\ &= i(Z_1Y_2Y_3Y_4\cdots Y_{n-1}Y_n)\cdot(Z_1Y_3X_4Z_5Y_7X_8\cdots X_{n-3}Z_{n-2}Y_n) \\ &= iY_2(Z_4X_5Y_6)(Z_8X_9Y_{10})\cdots(Z_{n-3}X_{n-2}Y_{n-1}). \end{aligned}$$

Thus, restricted to elements $g = I \otimes c$ with $c \in \mathfrak{su}(2^{n-1})$, the involution (C48) simplifies to:

$$c \mapsto -P_{YIZX}c^TP_{YIZX}, \qquad \text{for} \quad n \equiv 3 \bmod 4.$$

Corollary A.3 gives for the fixed-point subalgebra:

$$(P_{YIZX})^T = \begin{cases} P_{YIZX}, & n \equiv 7 \bmod 8, \\ -P_{YIZX}, & n \equiv 3 \bmod 8 \end{cases} \quad \Rightarrow \quad \tilde{\mathfrak{a}}_3(n) \cong \begin{cases} \mathfrak{so}(2^{n-1}), & n \equiv 7 \bmod 8, \\ \mathfrak{sp}(2^{n-2}), & n \equiv 3 \bmod 8. \end{cases}$$

This completes the proof of the lemma. □

Lemma C.31. *We have* $\mathfrak{a}_5(n) \cong \tilde{\mathfrak{a}}_5(n) = \mathfrak{g}_7(n)^{\tilde{\theta}_5} \cong \begin{cases} \mathfrak{so}(2^{n-1}), & n \equiv \pm 1 \bmod 6, \\ \mathfrak{sp}(2^{n-2}), & n \equiv 3 \bmod 6. \end{cases}$

Proof. The proof is analogous to that of Lemma C.30. Recall that U and $Q_5 = P_{IYZ}$ are given by (C44), (C39), and compute

$$\tilde{Q}_5 := UQ_5U^T = e^{i\frac{\pi}{4}Z\otimes Y^{\otimes(n-1)}} e^{i\frac{\pi}{4}Y\otimes X^{\otimes(n-1)}} P_{IYZ} e^{-i\frac{\pi}{4}Y\otimes X^{\otimes(n-1)}} e^{i\frac{\pi}{4}Z\otimes Y^{\otimes(n-1)}}.$$

When $n \equiv 3 \bmod 6$, $P_{IYZ} = Y_2Z_3Y_5Z_6Y_8Z_9\cdots Y_{n-1}Z_n$ commutes with $Y \otimes X^{\otimes(n-1)}$ and anticommutes with $Z \otimes Y^{\otimes(n-1)}$. Hence, by (A5), $\tilde{Q}_5 = P_{IYZ}$. Restricted to elements $g = I \otimes c$ with $c \in \mathfrak{su}(2^{n-1})$, the involution (C48) simplifies to:

$$c \mapsto -P_{YZI}c^TP_{YZI}, \qquad \text{for} \quad n \equiv 3 \bmod 6.$$

Since $(P_{YZI})^T = -P_{YZI}$, the fixed-point subalgebra is isomorphic to $\mathfrak{sp}(2^{n-2})$, by Corollary A.3.

For $n \equiv 1 \bmod 6$, $P_{IYZ} = Y_2Z_3Y_5Z_6\cdots Y_{n-2}Z_{n-1}$ commutes with both $Y \otimes X^{\otimes(n-1)}$ and $Z \otimes Y^{\otimes(n-1)}$. Hence, in this case,

$$\begin{aligned}\tilde{Q}_5 &= i(Z_1Y_2Y_3Y_4\cdots Y_{n-1}Y_n)\cdot(Y_2Z_3Y_5Z_6\cdots Y_{n-2}Z_{n-1})\\ &= i^{(n+2)/3}Z_1(X_3Y_4)(X_6Y_7)\cdots(X_{n-1}Y_n).\end{aligned}$$

The involution induced by (C48) on $c \in \mathfrak{su}(2^{n-1})$ is given by

$$c \mapsto -P_{IXY}c^TP_{IXY}, \qquad \text{for} \quad n \equiv 1 \bmod 6,$$

and the fixed-point subalgebra is isomorphic to $\mathfrak{so}(2^{n-1})$, because $(P_{IXY})^T = P_{IXY}$.

Finally, for $n \equiv 5 \bmod 6$, using (A6), we find

$$\begin{aligned}&e^{i\frac{\pi}{4}Y\otimes X^{\otimes(n-1)}} P_{IYZ} e^{-i\frac{\pi}{4}Y\otimes X^{\otimes(n-1)}}\\ &\qquad = i(Y_1X_2X_3X_4X_5\cdots X_n)(Y_2Z_3Y_5\cdots Z_{n-2}Y_n)\\ &\qquad = -Y_1Z_2Y_3X_4Z_5\cdots Y_{n-2}X_{n-1}Z_n.\end{aligned}$$

Then applying (A5), we get

$$\begin{aligned}\tilde{Q}_5 &= -i(Z_1Y_2Y_3Y_4Y_5\cdots Y_n)\cdot(Y_1Z_2Y_3X_4Z_5\cdots Y_{n-2}X_{n-1}Z_n)\\ &= -iX_1X_2(Z_4X_5)\cdots(Z_{n-1}X_n).\end{aligned}$$

This induces the involution on $\mathfrak{su}(2^{n-1})$ given by

$$c \mapsto -P_{XIZ}c^TP_{XIZ}, \qquad \text{for} \quad n \equiv 5 \bmod 6,$$

and the fixed-point subalgebra is isomorphic again to $\mathfrak{so}(2^{n-1})$. ◻

Lemma C.32. *We have* $\mathfrak{a}_3(n) \cong \tilde{\mathfrak{a}}_3(n) = \mathfrak{g}_7(n)^{\tilde{\theta}_3} \cong \begin{cases}\mathfrak{so}(2^{n-2})^{\oplus 4}, & n \equiv 0 \bmod 8,\\ \mathfrak{su}(2^{n-2})^{\oplus 2}, & n \equiv \pm 2 \bmod 8,\\ \mathfrak{sp}(2^{n-3})^{\oplus 4}, & n \equiv 4 \bmod 8.\end{cases}$

Proof. As in the proof of Lemma C.30, we need to compute

$$\tilde{Q}_3 := UQ_3U^T = e^{i\frac{\pi}{4}X_2} e^{i\frac{\pi}{4}I\otimes X\otimes Z^{\otimes(n-2)}} e^{i\frac{\pi}{4}Y\otimes X^{\otimes(n-1)}} P_{ZIYX} e^{-i\frac{\pi}{4}Y\otimes X^{\otimes(n-1)}} e^{i\frac{\pi}{4}I\otimes X\otimes Z^{\otimes(n-2)}} e^{i\frac{\pi}{4}X_2}.$$

Using (A5), (A6), we find for $n \equiv 0 \bmod 4$:

$$\tilde{Q}_3 = -Z_1(X_3Y_4Z_6)(X_7Y_8Z_{10})\cdots X_{n-1}Y_n.$$

Via the isomorphism $\mathfrak{g}_7(n) \cong \mathfrak{su}(2^{n-2})^{\oplus 4}$ from the proof of Lemma C.29 (see (C47)), the involution induced from (C48) on each copy of $\mathfrak{su}(2^{n-2})$ is given by

$$a_j \mapsto -P_{XYIZ}a_j^TP_{XYIZ}, \qquad \text{for} \quad 1 \le j \le 4, \quad n \equiv 0 \bmod 4.$$

For the fixed-point subalgebra, we get from Corollary A.3:

$$(P_{XYIZ})^T = \begin{cases}P_{XYIZ}, & n \equiv 0 \bmod 8,\\ -P_{XYIZ}, & n \equiv 4 \bmod 8\end{cases} \quad \Rightarrow \quad \tilde{\mathfrak{a}}_3(n) \cong \begin{cases}\mathfrak{so}(2^{n-2})^{\oplus 4}, & n \equiv 0 \bmod 8,\\ \mathfrak{sp}(2^{n-3})^{\oplus 4}, & n \equiv 4 \bmod 8.\end{cases}$$

Using (A5), (A6), we find for $n \equiv 0 \bmod 4$:

$$\tilde{Q}_3 = X_1X_2(Z_4X_5Y_6)(Z_8X_9Y_{10})\cdots(Z_{n-2}X_{n-1}Y_n).$$

Consider the unitary operator

$$V = \begin{cases} e^{i\frac{\pi}{4}Z_4X_5Y_6\cdots Z_{n-2}X_{n-1}Y_n}, & n \equiv 2 \bmod 8, \\ e^{i\frac{\pi}{4}Z_2Z_4X_5Y_6\cdots Z_{n-2}X_{n-1}Y_n}, & n \equiv 6 \bmod 8, \end{cases}$$

and perform the transformation $a \mapsto VaV^\dagger$ on $\mathfrak{g}_7(n)$. Since V commutes with Z_1 and Z_2, this transformation preserves the decomposition $\mathfrak{g}_7(n) \cong \mathfrak{su}(2^{n-2})^{\oplus 4}$ given by (C47). For $n \equiv 2 \bmod 8$, we have $V^T = V$ and V commutes with $\tilde{Q}_3$. Thus, $\tilde{Q}_3$ gets transformed to

$$V\tilde{Q}_3V^T = V^2\tilde{Q}_3 = i(Z_4X_5Y_6\cdots Z_{n-2}X_{n-1}Y_n)\cdot\tilde{Q}_3 = iX_1X_2.$$

The involution induced on $\mathfrak{g}_7(n)$ is given by

$$a \mapsto -X_1X_2a^TX_1X_2.$$

Writing a as in (C47), we note that

$$X_1X_2 \cdot P_1 \cdot X_1X_2 = P_4, \qquad X_1X_2 \cdot P_2 \cdot X_1X_2 = P_3.$$

Hence, $a = (a_1, a_2, a_3, a_4)$ is a fixed point of the above involution if and only if $a_4 = -a_1^T$, $a_3 = -a_2^T$. Sending such a to (a_1, a_2) gives an isomorphism from the fixed-point subalgebra to $\mathfrak{su}(2^{n-2})^{\oplus 2}$.

When $n \equiv 6 \bmod 8$, we have $V^T = V^{-1}$ and V anticommutes with $\tilde{Q}_3$. Thus, $\tilde{Q}_3$ gets transformed to

$$V\tilde{Q}_3V^T = V\tilde{Q}_3V^{-1} = V^2\tilde{Q}_3 = i(Z_2Z_4X_5Y_6\cdots Z_{n-2}X_{n-1}Y_n)\cdot\tilde{Q}_3 = -X_1Y_2.$$

The rest of the proof is similar to the case $n \equiv 2 \bmod 8$ above. □

Lemma C.33. *We have* $\mathfrak{a}_5(n) \cong \tilde{\mathfrak{a}}_5(n) = \mathfrak{g}_7(n)^{\tilde{\theta}_5} \cong \begin{cases} \mathfrak{so}(2^{n-2})^{\oplus 4}, & n \equiv 0 \bmod 6, \\ \mathfrak{su}(2^{n-2})^{\oplus 2}, & n \equiv \pm 2 \bmod 6. \end{cases}$

Proof. The proof is very similar to Lemma C.32, so we only indicate the differences. We compute

$$\tilde{Q}_5 := UQ_5U^T = e^{i\frac{\pi}{4}X_2}e^{i\frac{\pi}{4}I\otimes X\otimes Z^{\otimes(n-2)}}e^{i\frac{\pi}{4}Y\otimes X^{\otimes(n-1)}}P_{IYZ}e^{-i\frac{\pi}{4}Y\otimes X^{\otimes(n-1)}}e^{i\frac{\pi}{4}I\otimes X\otimes Z^{\otimes(n-2)}}e^{i\frac{\pi}{4}X_2},$$

and find that

$$\tilde{Q}_5 = \begin{cases} i^{-n/3}Z_2(Z_4X_5)(Z_7X_8)\cdots X_{n-1}, & n \equiv 0 \bmod 6, \\ -Y_1Z_2(Y_3X_4Z_5)(Y_6X_7Z_8)\cdots Z_n, & n \equiv 2 \bmod 6, \\ P_{IYZ} = (Y_2Z_3)(Y_5Z_6)(Y_8Z_9)\cdots Z_{n-1}, & n \equiv 4 \bmod 6. \end{cases}$$

For $n \equiv 0 \bmod 6$, the involution induced from (C48) on each copy of $\mathfrak{su}(2^{n-2})$ from the decomposition (C47) is given by

$$a_j \mapsto -P_{IZX}a_j^TP_{IZX}, \qquad \text{for} \quad 1 \le j \le 4, \quad n \equiv 0 \bmod 6.$$

For $n \equiv \pm 2 \bmod 6$, we use the transformation $a \mapsto VaV^\dagger$, where

$$V = \begin{cases} e^{i\frac{\pi}{4}Z_2Y_3X_4Z_5Y_6X_7Z_8\cdots Z_n}, & n \equiv 2 \bmod 6, \\ e^{i\frac{\pi}{4}Z_3Y_5Z_6Y_8Z_9\cdots Z_{n-1}}, & n \equiv 4 \bmod 6, \end{cases}$$

which allows us to replace $\tilde{Q}_5$ with $V\tilde{Q}_5V^T$. This gives the involutions $a \mapsto -Y_1a^TY_1$ and $a \mapsto -Y_2a^TY_2$ for $n \equiv 2$ and $n \equiv 4 \bmod 6$, respectively. □

VIII. Periodic boundary conditions

Recall that the subalgebras $\mathfrak{a}_k^\circ(n), \mathfrak{b}_l^\circ(n) \subseteq \mathfrak{su}(2^n)$ $(0 \le k \le 22,\ 0 \le l \le 4)$ are defined by (B6). In this subsection, we prove Theorem IV.2, which we reproduce here for convenience:

$$\begin{aligned}
&\mathfrak{a}_0^\circ(n) \cong \mathfrak{u}(1)^{\oplus n},\\
&\mathfrak{a}_1^\circ(n) \cong \mathfrak{so}(n)^{\oplus 2},\\
&\mathfrak{a}_2^\circ(n) \cong \mathfrak{so}(n)^{\oplus 4},\\
&\mathfrak{a}_3^\circ(n) = \begin{cases} \mathfrak{a}_{13}(n), & n \text{ odd},\\ \mathfrak{a}_3(n), & n \equiv 0 \mod 4,\\ \mathfrak{a}_6(n), & n \equiv 2 \mod 4,\end{cases}\\
&\mathfrak{a}_4^\circ(n) \cong \begin{cases} \mathfrak{so}(2n), & n \text{ odd},\\ \mathfrak{so}(n)^{\oplus 4}, & n \text{ even},\end{cases}\\
&\mathfrak{a}_5^\circ(n) = \begin{cases} \mathfrak{a}_{16}(n), & n \equiv \pm 1 \mod 3,\\ \mathfrak{a}_5(n), & n \equiv 0 \mod 3,\end{cases}\\
&\mathfrak{a}_6^\circ(n) = \begin{cases} \mathfrak{a}_{13}(n), & n \text{ odd},\\ \mathfrak{a}_6(n), & n \text{ even},\end{cases}\\
&\mathfrak{a}_k^\circ(n) = \mathfrak{a}_k(n), \quad k = 7, 13, 16, 20,\\
&\mathfrak{a}_8^\circ(n) \cong \mathfrak{so}(2n)^{\oplus 2},\\
&\mathfrak{a}_9^\circ(n) = \mathfrak{b}_2^\circ(n) \cong \mathfrak{so}(2^n), \quad n \ge 4,\\
&\mathfrak{a}_{10}^\circ(n) = \begin{cases} \mathfrak{su}(2^n), & n \equiv \pm 1 \mod 3,\\ \mathfrak{a}_{10}(n), & n \equiv 0 \mod 3,\end{cases}\\
&\mathfrak{a}_{11}^\circ(n) = \mathfrak{so}(2^n), \quad n \ge 4,\\
&\mathfrak{a}_k^\circ(n) = \mathfrak{b}_4^\circ(n) = \mathfrak{su}(2^n), \quad k = 12, 15, 17, 18, 19, 21, 22,\\
&\mathfrak{a}_{14}^\circ(n) \cong \mathfrak{so}(2n)^{\oplus 2},\\
&\mathfrak{b}_0^\circ(n) = \mathfrak{b}_0(n) \cong \mathfrak{u}(1)^{\oplus n},\\
&\mathfrak{b}_1^\circ(n) \cong \mathfrak{u}(1)^{\oplus 2n},\\
&\mathfrak{b}_3^\circ(n) = \mathfrak{b}_3(n) \cong \mathfrak{su}(2)^{\oplus n}.
\end{aligned}$$

We start the **proof** by observing that due to (B3), (B4) and from $\dim \mathfrak{a}_{12}^\circ(3) = \dim \mathfrak{a}_{17}^\circ(3) = 63$, we have:

$$\mathfrak{a}_k^\circ(n) = \mathfrak{su}(2^n), \qquad k = 12, 17, 18, 19, 21, 22, \quad n \ge 3.$$

Moreover,

$$\mathfrak{a}_{15}^\circ(n) = \mathfrak{su}(2^n), \qquad n \ge 3,$$

because $\mathfrak{a}_{15}(n)$ contains (i times) all Pauli strings that start with X or I, except $I^{\otimes n}$. Then, applying the cyclic shift τ_n defined in (B7), we can generate all Pauli strings $\neq I^{\otimes n}$.

We also note that

$$\mathfrak{a}_k^\circ(n) = \mathfrak{a}_k(n), \qquad k = 7, 13, 16, 20, \quad n \ge 3; \qquad \mathfrak{a}_{11}^\circ(n) = \mathfrak{a}_{11}(n), \qquad n \ge 4,$$

due to (C5), (C6) and Lemmas C.21, C.24, C.25, because in this case $\tau_n \mathfrak{a}_k(n) \subseteq \mathfrak{a}_k(n)$.

In Sect. C III, using frustration graphs, we have determined the Lie algebras $\mathfrak{a}_k^\circ(n)$ for $k = 1, 2, 4, 8, 14$ (see Lemmas C.7, C.8, C.9). It is also obvious that

$$\begin{aligned}
&\mathfrak{b}_0^\circ(n) = \mathfrak{b}_0(n), \qquad && \mathfrak{b}_2^\circ(n) = \mathfrak{a}_9^\circ(n),\\
&\mathfrak{b}_3^\circ(n) = \mathfrak{b}_3(n), && \mathfrak{b}_4^\circ(n) = \mathfrak{a}_{15}^\circ(n),\\
&\mathfrak{b}_1^\circ(n) = \operatorname{span}\{X_i, X_1X_n, X_jX_{j+1}\}_{1 \le i \le n,\ 1 \le j \le n-1} \cong \mathfrak{u}(1)^{\oplus 2n},\\
&\mathfrak{a}_0^\circ(n) = \operatorname{span}\{X_1X_n, X_jX_{j+1}\}_{1 \le j \le n-1} \cong \mathfrak{u}(1)^{\oplus n}.
\end{aligned}$$

We discuss the remaining cases $\mathfrak{a}_k^\circ(n)$ $(k = 3, 5, 6, 9, 10)$ in a sequence of lemmas.

Lemma C.34. *We have* $\mathfrak{a}_{10}^{\circ}(n) = \begin{cases} \mathfrak{su}(2^n), & n \equiv \pm 1 \mod 3, \\ \mathfrak{a}_{10}(n), & n \equiv 0 \mod 3. \end{cases}$

Proof. Recall from Theorem C.1 that $\mathfrak{a}_{10}(n) = \mathfrak{g}_{10}(n)$ where $\mathfrak{g}_{10}(n)$ is given by (C26). When $n \equiv 0$ mod 3, we have:

$$\tau_n P_{XYZ} = P_{YZX}, \qquad \tau_n P_{YZX} = P_{ZXY}, \qquad \tau_n P_{ZXY} = P_{XYZ},$$

which imply that $\tau_n \mathfrak{a}_{10}(n) \subseteq \mathfrak{a}_{10}(n)$, and hence $\mathfrak{a}_{10}^{\circ}(n) = \mathfrak{a}_{10}(n)$.

On the other hand, for $n \equiv 1$ mod 3, we have:

$$\begin{aligned} \tau_n^{-1} P_{XYZ} &= X \otimes P_{XYZ} = XXYZXYZ \cdots XYZ, \\ \tau_n^{-1} P_{YZX} &= Y \otimes P_{YZX} = YYZXYZX \cdots YZX, \\ \tau_n^{-1} P_{ZXY} &= Z \otimes P_{ZXY} = ZZXYZXY \cdots ZXY. \end{aligned}$$

In particular, their centralizer contains the elements

$$X_1X_2, Y_1Y_2, Z_1Z_2 \in \tau_n^{-1}\mathfrak{a}_{10}(n) \subset \mathfrak{a}_{10}^{\circ}(n).$$

From these elements and

$$X_1Y_2, Y_1Z_2, Z_1X_2 \in \mathfrak{a}_{10}(n) \subset \mathfrak{a}_{10}^{\circ}(n),$$

we can generate all 2-qubit gates: $\mathfrak{su}(4) \otimes I^{\otimes(n-2)} \subset \mathfrak{a}_{10}^{\circ}(n)$. Therefore, $\mathfrak{a}_{10}^{\circ}(n) = \mathfrak{su}(2^n)$.

The case $n \equiv -1$ mod 3 is similar. □

Lemma C.35. *We have* $\mathfrak{a}_{5}^{\circ}(n) = \begin{cases} \mathfrak{a}_{16}(n), & n \equiv \pm 1 \mod 3, \\ \mathfrak{a}_{5}(n), & n \equiv 0 \mod 3. \end{cases}$

Proof. Recall the automorphism γ_n of $\mathfrak{su}(2^n)$ defined by (C11), (C12). Then, by Lemma C.21, $\gamma_n \mathfrak{a}_5(n) = \mathfrak{g}_7(n)^{\tilde{\theta}_5}$, where $\tilde{\theta}_5$ is given by (C37), (C39), and $\mathfrak{g}_7(n)$ is given by (C27). From this, we get

$$\gamma_n \tau_n \mathfrak{a}_5(n) = (\gamma_n \tau_n \gamma_n^{-1}) \gamma_n \mathfrak{a}_5(n) = (\gamma_n \tau_n \gamma_n^{-1}) \mathfrak{g}_7(n)^{\tilde{\theta}_5}.$$

When $n \equiv 0$ mod 3, we have

$$(\gamma_n \tau_n \gamma_n^{-1})(P_X) = P_Y, \qquad (\gamma_n \tau_n \gamma_n^{-1})(P_Y) = P_Z, \qquad (\gamma_n \tau_n \gamma_n^{-1})(P_Z) = P_X,$$

which imply that $(\gamma_n \tau_n \gamma_n^{-1}) \mathfrak{g}_7(n) \subseteq \mathfrak{g}_7(n)$. Next, we compute (cf. (C39)):

$$\begin{aligned} (\gamma_n \tau_n \gamma_n^{-1}) Q_5 &= (\gamma_n \tau_n \gamma_n^{-1})(P_{IYZ}) \\ &= Z_1 X_2 Z_4 X_5 \cdots Z_{n-2} X_{n-1} \\ &= i^{n/3} P_Z \cdot Q_5 = i^{-n/3} Q_5 \cdot P_Z. \end{aligned}$$

From this, we deduce that $\tilde{\theta}_5$ commutes with $\gamma_n \tau_n \gamma_n^{-1}$. Indeed, as it commutes with the trace, we find for $g \in \mathfrak{g}_7(n)$:

$$(\gamma_n \tau_n \gamma_n^{-1}) \tilde{\theta}_5(g) = -(\gamma_n \tau_n \gamma_n^{-1} Q_5) h^T (\gamma_n \tau_n \gamma_n^{-1} Q_5) = -Q_5 \cdot P_Z h^T P_Z \cdot Q_5 = -Q h^T Q = \tilde{\theta}_5(h),$$

where we set $h := (\gamma_n \tau_n \gamma_n^{-1}) g$ and use that $h, h^T \in \mathfrak{g}_7(n)$. Therefore, $\gamma_n \tau_n \mathfrak{a}_5(n) \subseteq \gamma_n \mathfrak{a}_5(n)$, and hence $\mathfrak{a}_5^{\circ}(n) = \mathfrak{a}_5(n)$ for $n \equiv 0$ mod 3.

Suppose now that $n \equiv 1$ mod 3. Observe that $\mathfrak{a}_5^{\circ}(n) \subseteq \mathfrak{so}(2^n) = \mathfrak{a}_{16}(n)$ for all $n \geq 2$, because all generators of $\mathfrak{a}_5^{\circ}(n)$ have an odd number of Y's. On the other hand, we have

$$X_2 X_n \in \mathfrak{g}_7(n)^{\tilde{\theta}_5} = \gamma_n \mathfrak{a}_5(n) \;\Rightarrow\; \gamma_n^{-1}(X_2 X_n) = Z_2 Y_n \in \mathfrak{a}_5(n) \;\Rightarrow\; \tau_n^{-1}(Z_2 Y_n) = Y_1 Z_3 \in \mathfrak{a}_5^{\circ}(n).$$

Since $Y_1 X_3 \in \mathfrak{a}_5(3)$ (see Sect. B V), we get that $Y_1 X_3 \in \mathfrak{a}_5^{\circ}(n)$. Hence, $[Y_1 Z_3, Y_1 X_3] = 2i Y_3 \in \mathfrak{a}_5^{\circ}(n)$, and cyclic shifts give $Y_1, Y_2 \in \mathfrak{a}_5^{\circ}(n)$. Together with $\mathfrak{a}_5 = \langle XY, YZ \rangle_{\mathrm{Lie}}$, the elements YI, IY can generate $\mathfrak{a}_{16} = \langle XY, YX, YZ, ZY \rangle_{\mathrm{Lie}}$. Therefore, $\mathfrak{a}_5^{\circ}(n) \supseteq \mathfrak{a}_{16}(n)$, which proves that $\mathfrak{a}_5^{\circ}(n) = \mathfrak{a}_{16}(n)$.

Similarly, in the case $n \equiv -1$ mod 3, we have:

$$\begin{aligned} X_1 X_n \in \mathfrak{g}_7(n)^{\tilde{\theta}_5} = \gamma_n \mathfrak{a}_5(n) \;&\Rightarrow\; \gamma_n^{-1}(X_1 X_n) = Y_1 Z_n \in \mathfrak{a}_5(n) \;\Rightarrow\; \tau_n^{-1}(Y_1 Z_n) = Z_1 Y_2 \in \mathfrak{a}_5^{\circ}(n), \\ Z_1 Z_n \in \mathfrak{g}_7(n)^{\tilde{\theta}_5} = \gamma_n \mathfrak{a}_5(n) \;&\Rightarrow\; \gamma_n^{-1}(Z_1 Z_n) = X_1 Y_n \in \mathfrak{a}_5(n) \;\Rightarrow\; \tau_n^{-1}(X_1 Y_n) = Y_1 X_2 \in \mathfrak{a}_5^{\circ}(n). \end{aligned}$$

Hence, $\mathfrak{a}_5^{\circ}(n)$ contains $\mathfrak{a}_{16}(n)$, so it must be equal to it. □

Lemma C.36. *We have* $\mathfrak{a}_9^\circ(n) \cong \mathfrak{so}(2^n)$ *for* $n \geq 4$.

Proof. First, recall from Lemma C.23 that $\mathfrak{a}_9(n) = \mathfrak{g}_9(n)^{\theta_9}$, where $\mathfrak{g}_9(n)$ is given by (C28), $\theta_9(g) = -Q_9 g^T Q_9$, and $Q_9 = IYZ\cdots Z$ is given by (C35). For example, $g = Y_3X_4 \in \mathfrak{a}_9(n)$, as $g^T = -g$ and g commutes with X_1, Y_1X_2, Z_1X_2 and Q_9 (or one can check directly that $IIYX \in \mathfrak{a}_9(4)$). Similarly, we check that $Z_3X_4 \in \mathfrak{a}_9(n)$.

Now let us relabel $X \rightleftharpoons Y$, so that $\mathfrak{a}_9 = \langle YX, YZ\rangle_{\text{Lie}}$. Then $\mathfrak{a}_9^\circ(n) \subseteq \mathfrak{so}(2^n) = \mathfrak{a}_{16}(n)$, because all generators of $\mathfrak{a}_9^\circ(n)$ contain an odd number of Y's. From above after relabeling, we have $X_3Y_4, Z_3Y_4 \in \mathfrak{a}_9(n)$, which after a cyclic shift gives $X_1Y_2, Z_1Y_2 \in \mathfrak{a}_9^\circ(n)$. Since $\mathfrak{a}_{16} = \langle XY, YX, YZ, ZY\rangle_{\text{Lie}}$, we obtain that $\mathfrak{a}_9^\circ(n) \supseteq \mathfrak{a}_{16}(n)$. □

Lemma C.37. *We have:*

$$\mathfrak{a}_6^\circ(n) = \mathfrak{a}_6(n), \quad n \text{ even}, \qquad \mathfrak{a}_3^\circ(n) = \mathfrak{a}_6^\circ(n) = \mathfrak{a}_{13}(n), \quad n \text{ odd},$$
$$\mathfrak{a}_3^\circ(n) = \mathfrak{a}_6(n), \quad n \equiv 2 \mod 4, \qquad \mathfrak{a}_3^\circ(n) = \mathfrak{a}_3(n), \quad n \equiv 0 \mod 4.$$

Proof. First of all, note that $\mathfrak{a}_3^\circ(n) \subseteq \mathfrak{a}_6^\circ(n)$ for all n, because $\mathfrak{a}_3 \subset \mathfrak{a}_6$. By Lemma C.22, we have for even n:

$$\mathfrak{a}_6(n) = \mathfrak{g}_6(n) = \mathfrak{su}(2^n)^{\{P_X, P_{YZ}, P_{ZY}\}}/\text{span}\{P_X, P_{YZ}, P_{ZY}\}.$$

In this case,

$$\tau_n(P_X) = P_X, \qquad \tau_n(P_{YZ}) = P_{ZY}, \qquad \tau_n(P_{ZY}) = P_{YZ},$$

which implies that $\tau_n\mathfrak{a}_6(n) \subseteq \mathfrak{a}_6(n)$, and hence $\mathfrak{a}_6^\circ(n) = \mathfrak{a}_6(n)$.

Recall the automorphism φ_n of $\mathfrak{su}(2^n)$ that up to a sign swaps Y and Z on all even qubits; see (C8), (C9). By Lemma C.21, we have $\varphi_n\mathfrak{a}_3(n) = \mathfrak{g}_7(n)^{\tilde\theta_3}$, where $\tilde\theta_3$ is given by (C37), (C38), and $\mathfrak{g}_7(n)$ is given by (C27). Hence,

$$\varphi_n\tau_n\mathfrak{a}_3(n) = (\varphi_n\tau_n\varphi_n^{-1})\varphi_n\mathfrak{a}_3(n) = (\varphi_n\tau_n\varphi_n^{-1})\mathfrak{g}_7(n)^{\tilde\theta_3}.$$

When n is even, we have:

$$(\varphi_n\tau_n\varphi_n^{-1})(P_X) = P_X, \qquad (\varphi_n\tau_n\varphi_n^{-1})(P_Y) = P_Z, \qquad (\varphi_n\tau_n\varphi_n^{-1})(P_Z) = P_Y,$$

which implies that $(\varphi_n\tau_n\varphi_n^{-1})\mathfrak{g}_7(n) \subseteq \mathfrak{g}_7(n)$. For $n \equiv 0 \mod 4$, we find

$$(\varphi_n\tau_n\varphi_n^{-1})Q_3 = (\varphi_n\tau_n\varphi_n^{-1})(P_{ZIYX}) = P_{IZXY} = P_Z \cdot Q_3 = Q_3 \cdot P_Z.$$

Then, as in the proof of Lemma C.35, we conclude that in this case $\mathfrak{a}_3^\circ(n) = \mathfrak{a}_3(n)$.

Next, in the case $n \equiv 2 \mod 4$, one checks that

$$Y_1Y_n \in \mathfrak{g}_7(n)^{\tilde\theta_3} = \varphi_n\mathfrak{a}_3(n) \;\Rightarrow\; \varphi_n^{-1}(Y_1Y_n) = Y_1Z_n \in \mathfrak{a}_3(n) \;\Rightarrow\; \tau_n^{-1}(Y_1Z_n) = Z_1Y_2 \in \mathfrak{a}_3^\circ(n).$$

Hence, $\mathfrak{a}_3^\circ(n)$ contains all generators of $\mathfrak{a}_6(n)$, proving that $\mathfrak{a}_3^\circ(n) = \mathfrak{a}_6(n)$.

Finally, consider the case where n is odd. Recall that, by Lemma C.24,

$$\mathfrak{a}_3^\circ(n) \subseteq \mathfrak{a}_6^\circ(n) \subseteq \mathfrak{a}_{13}(n) = \mathfrak{su}(2^n)^{P_X}/\text{span}\{P_X\}.$$

In order to prove that these are equalities, it is enough to show that $\mathfrak{a}_3^\circ(n)$ contains the generators of $\mathfrak{a}_{13}(n)$. When $n \equiv 1 \mod 4$, we have

$$Z_1Z_{n-1} \in \mathfrak{g}_7(n)^{\tilde\theta_3} = \varphi_n\mathfrak{a}_3(n) \;\Rightarrow\; \varphi_n^{-1}(Z_1Z_{n-1}) = -Z_1Y_{n-1} \in \mathfrak{a}_3(n) \;\Rightarrow\; \tau_n^{-2}(Z_1Y_{n-1}) = Y_1Z_3 \in \mathfrak{a}_3^\circ(n).$$

Since $ZIZ \in \mathfrak{a}_3(3)$, we get that $Z_1Z_3 \in \mathfrak{a}_3^\circ(n)$ and hence $X_1 = -\frac{i}{2}[Y_1Z_3, Z_1Z_3] \in \mathfrak{a}_3^\circ(n)$. Similarly, when $n \equiv 3 \mod 4$, we have

$$Z_1Z_n \in \mathfrak{g}_7(n)^{\tilde\theta_3} = \varphi_n\mathfrak{a}_3(n) \;\Rightarrow\; \varphi_n^{-1}(Z_1Z_n) = Z_1Z_n \in \mathfrak{a}_3(n) \;\Rightarrow\; \tau_n^{-1}(Z_1Z_n) = Z_1Z_2 \in \mathfrak{a}_3^\circ(n).$$

Then from $Y_1Z_2 \in \mathfrak{a}_3^\circ(n)$, we get again that $X_1 \in \mathfrak{a}_3^\circ(n)$. Therefore, all $X_i \in \mathfrak{a}_3^\circ(n)$, and we can generate $\mathfrak{a}_{13}(n)$ from them and the generators X_iX_{i+1}, Y_iZ_{i+1} of $\mathfrak{a}_3(n)$. □

The above lemmas complete the proof of Theorem IV.2.

IX. Permutation-invariant subalgebras

In this subsection, we classify all permutation-invariant subalgebras of $\mathfrak{su}(2^n)$ that are generated by single Paulis and products of two Paulis, thus proving Theorem IV.3. Recall that, starting from a subalgebra $\mathfrak{a} \subseteq \mathfrak{su}(4)$ generated by Pauli strings, we define the subalgebra $\mathfrak{a}^\pi(n) \subseteq \mathfrak{su}(2^n)$ given by (B10). Moreover, in Sect. B IV, we explained that $\mathfrak{a}$ can be assumed itself invariant under the flip of the two qubits; so we only need to consider $\mathfrak{a}_k^\pi(n)$ for $k = 0, 2, 4, 6, 7, 14, 16, 20$ and $\mathfrak{b}_l^\pi(n)$ for $l = 0, 1, 3$. The complete list of such Lie algebras is presented in Theorem IV.3 and reproduced here as follows:

$$\begin{aligned}
\mathfrak{a}_k^\pi(n) &= \mathfrak{a}_k(n), \qquad k = 7, 16, 20, 22,\\
\mathfrak{a}_0^\pi(n) &\cong \mathfrak{u}(1)^{\oplus n(n-1)/2},\\
\mathfrak{a}_2^\pi(n) &= \mathfrak{so}(2^n)^{P_Z} \cong \mathfrak{so}(2^{n-1})^{\oplus 2},\\
\mathfrak{a}_4^\pi(n) &= \mathfrak{a}_7(n),\\
\mathfrak{a}_{14}^\pi(n) &\cong \mathfrak{a}_6^\pi(n) = \mathfrak{a}_{20}(n),\\
\mathfrak{b}_l^\pi(n) &= \mathfrak{b}_l(n), \qquad l = 0, 3,\\
\mathfrak{b}_1^\pi(n) &\cong \mathfrak{u}(1)^{\oplus n(n+1)/2}.
\end{aligned}$$

To start the **proof** of the theorem, we first observe that the following subalgebras of $\mathfrak{su}(2^n)$ are permutation invariant, due to their explicit descriptions (cf. Theorem C.1):

$$\begin{aligned}
\mathfrak{a}_7(n) &= \begin{cases} \mathfrak{su}(2^n)^{\{P_X, P_Y, P_Z\}}/\mathrm{span}\{P_X, P_Y, P_Z\}, & n \text{ even},\\ \mathfrak{su}(2^n)^{\{P_X, P_Y, P_Z\}}, & n \text{ odd}, \end{cases}\\
\mathfrak{a}_{16}(n) &= \mathfrak{so}(2^n),\\
\mathfrak{a}_{20}(n) &= \mathfrak{su}(2^n)^{P_X}/\mathrm{span}\{P_X\},\\
\mathfrak{a}_{22}(n) &= \mathfrak{su}(2^n),\\
\mathfrak{b}_0(n) &= \mathrm{span}\{X_i\}_{1\le i\le n},\\
\mathfrak{b}_3(n) &= \mathrm{span}\{X_i, Y_i, Z_i\}_{1\le i\le n}.
\end{aligned}$$

It is also easy to see that

$$\begin{aligned}
\mathfrak{a}_0^\pi(n) &= \mathrm{span}\{X_i X_j\}_{1\le i<j\le n},\\
\mathfrak{b}_1^\pi(n) &= \mathrm{span}\{X_k, X_i X_j\}_{1\le i<j\le n,\ 1\le k\le n}.
\end{aligned}$$

Thus, we are left to determine $\mathfrak{a}_k^\pi(n)$ for $k = 2, 4, 6, 14$. These cases are treated in the next three lemmas.

Lemma C.38. *We have $\mathfrak{a}_2^\pi(n) = \mathfrak{so}(2^n)^{P_Z}$ for all $n \ge 2$.*

Proof. Note that all generators $X_i Y_j$ $(i \ne j)$ of $\mathfrak{a}_2^\pi(n)$ commute with P_Z and are skew-symmetric, i.e., satisfy $a^T = -a$. Hence, $\mathfrak{a}_2^\pi(n) \subseteq \mathfrak{so}(2^n)^{P_Z}$. For the opposite inclusion, we use the same strategy as in the proof of Lemma C.24. Pick an arbitrary Pauli string $a \in \mathfrak{so}(2^n)^{P_Z}$ not containing any I's; then we want to find a Pauli string $b \in \mathfrak{a}_2^\pi(n)$ such that $[a, b] \ne 0$ and $[a, b]$ has an I in some position. Note that a has an odd number of X's and an odd number of Y's. In particular, after a permutation, a must start with XYZ, XXY, or XYY. Then we let $b = X_1 Y_3$, $X_1 Z_2 Y_3$, or $X_1 Z_2 Y_3$, respectively. Here $b \in \mathfrak{a}_2^\pi(n)$ because $XZY \in \mathfrak{a}_2(3)$; cf. Sect. B V. □

Lemma C.39. *We have $\mathfrak{a}_{14}^\pi(n) \cong \mathfrak{a}_6^\pi(n) = \mathfrak{a}_{20}(n)$ for $n \ge 3$.*

Proof. Let us relabel $X \rightleftharpoons Z$ in $\mathfrak{a}_{14}$. Then $\mathfrak{a}_{14} \subset \mathfrak{a}_{20}$, which implies $\mathfrak{a}_{14}^\pi(n) \subseteq \mathfrak{a}_{20}^\pi(n) = \mathfrak{a}_{20}(n)$ for all n. Similarly, from $\mathfrak{a}_6 \subset \mathfrak{a}_{20}$, we get $\mathfrak{a}_6^\pi(n) \subseteq \mathfrak{a}_{20}(n)$. To finish the proof of the lemma, it is enough to show that $\mathfrak{a}_6^\pi(3) = \mathfrak{a}_{14}^\pi(3) = \mathfrak{a}_{20}(3)$, because $\mathfrak{a}_{20}(n)$ is generated from $\mathfrak{a}_{20}(3)$ using a process similar to (B1). The claim now follows from $\mathfrak{a}_k^\circ(3) \subseteq \mathfrak{a}_k^\pi(3)$ and

$$\dim \mathfrak{a}_6^\circ(3) = \dim \mathfrak{a}_{14}^\circ(3) = \dim \mathfrak{a}_{20}(3) = 30;$$

see Sect. B V. □

Lemma C.40. *We have $\mathfrak{a}_4^\pi(n) = \mathfrak{a}_7(n)$ for $n \ge 3$.*

Proof. Since $\mathfrak{a}_4 \subset \mathfrak{a}_7$, we have $\mathfrak{a}_4^\pi(n) \subseteq \mathfrak{a}_7^\pi(n) = \mathfrak{a}_7(n)$ for all $n \geq 3$. To prove the opposite inclusion, it is enough to show that $\mathfrak{a}_4^\pi(3) = \mathfrak{a}_7(3)$, because $\mathfrak{a}_7(n)$ is generated from $\mathfrak{a}_7(3)$ using a process similar to (B1). From $IXX, YZX \in \mathfrak{a}_4(3)$, we get $ZYX \in \mathfrak{a}_4^\pi(3)$ and $[IXX, ZYX] = 2iZZI \in \mathfrak{a}_4^\pi(3)$. Then, by permutation invariance, also $IZZ \in \mathfrak{a}_4^\pi(3)$. Hence, $\mathfrak{a}_4^\pi(3)$ contains all generators of $\mathfrak{a}_7(3)$, so it must be equal to it. □

The only thing left to finish the proof of Theorem IV.3 is to show that $\mathfrak{so}(2^n)^{P_Z} \cong \mathfrak{so}(2^{n-1})^{\oplus 2}$. This follows from the isomorphism $\mathfrak{su}(2^n)^{P_Z}/\text{span}\{P_Z\} \cong \mathfrak{su}(2^{n-1})^{\oplus 2}$ (see Lemma C.27), which is compatible with taking matrix transpose.

---

[1] R. Zeier and T. Schulte-Herbrüggen, Symmetry principles in quantum systems theory, Journal of mathematical physics **52**, 113510 (2011).
[2] D. d'Alessandro, *Introduction to quantum control and dynamics*, 2nd ed. (Chapman and hall/CRC, 2021).
[3] A. W. Knapp, *Lie Groups Beyond an Introduction*, Vol. 140 (Springer Science & Business Media, 2013).
[4] R. Wiersema, Classification of dynamical Lie algebras for translation-invariant 2-local spin systems in one dimension (2023), https://github.com/therooler/lie_classification.
[5] R. J. Baxter, *Exactly solved models in statistical mechanics* (Elsevier, 1982).
[6] F. Franchini *et al.*, *An introduction to integrable techniques for one-dimensional quantum systems*, Vol. 940 (Springer, 2017).
[7] A. Chapman and S. T. Flammia, Characterization of solvable spin models via graph invariants, Quantum **4**, 278 (2020).
[8] Y. Atia and D. Aharonov, Fast-forwarding of hamiltonians and exponentially precise measurements, Nature communications **8**, 1 (2017).
[9] S. Gu, R. D. Somma, and B. Şahinoğlu, Fast-forwarding quantum evolution, Quantum **5**, 577 (2021).
[10] L. Bassman Oftelie, R. Van Beeumen, E. Younis, E. Smith, C. Iancu, and W. A. de Jong, Constant-depth circuits for dynamic simulations of materials on quantum computers, Materials Theory **6**, 13 (2022).
[11] E. Kökcü, T. Steckmann, Y. Wang, J. K. Freericks, E. F. Dumitrescu, and A. F. Kemper, Fixed depth hamiltonian simulation via cartan decomposition, Phys. Rev. Lett. **129**, 070501 (2022).
[12] E. Kökcü, D. Camps, L. Bassman, J. K. Freericks, W. A. de Jong, R. Van Beeumen, and A. F. Kemper, Algebraic compression of quantum circuits for hamiltonian evolution, Physical Review A **105**, 032420 (2022).
[13] D. Camps, E. Kökcü, L. Bassman, W. A. de Jong, A. F. Kemper, and R. V. Beeumen, An algebraic quantum circuit compression algorithm for hamiltonian simulation, SIAM Journal on Matrix Analysis and Applications **43**, 1084 (2022).
[14] E. Kökcü, D. Camps, L. B. Oftelie, W. A. de Jong, R. Van Beeumen, and A. Kemper, Algebraic compression of free fermionic quantum circuits: Particle creation, arbitrary lattices and controlled evolution, arXiv preprint arXiv:2303.09538 10.48550/arXiv.2303.09538 (2023).
[15] C. Procesi, *Lie groups: an approach through invariants and representations*, Vol. 115 (Springer, 2007).
